# Genome-wide association study identified novel loci and gene-environment interaction for refractive error in children

**DOI:** 10.1038/s41525-025-00504-5

**Published:** 2025-05-23

**Authors:** Yuyao Wang, Yuzhou Zhang, Haoyu Chen, Xiu Juan Zhang, Riping Zhang, Tsz Kin Ng, Jenson A. Tham, Ka Wai Kam, Pancy O. S. Tam, Alvin L. Young, Yingying Wei, Mingzhi Zhang, Chi Pui Pang, Clement C. Tham, Jason C. Yam, Li Jia Chen

**Affiliations:** 1 Department of Ophthalmology and Visual Sciences, The Chinese University of Hong Kong, Hong Kong, China; 2 Joint Shantou International Eye Center of Shantou University and the Chinese University of Hong Kong, Guangdong Shantou, China; 3 Department of Ophthalmology, LKS Faculty of Medicine, The University of Hong Kong, Hong Kong, China; 4 Queen Mary Hospital, Pok Fu Lam, Hong Kong, China; 5 Department of Ophthalmology and Visual Sciences, Prince of Wales Hospital, Hong Kong, China; 6 Department of Statistics, The Chinese University of Hong Kong, Hong Kong, China; 7 Hong Kong Hub of Paediatric Excellence, The Chinese University of Hong Kong, Hong Kong, China; 8 Hong Kong Eye Hospital, Hong Kong, China

**Keywords:** Risk factors, Genetics

## Abstract

To identify novel genetic loci for children refractive error, we performed a meta-analysis of two genome-wide association studies (GWASs) of spherical equivalent (SE) in 1,237 children from the population-based Hong Kong Children Eye Study (HKCES) and the Low Concentration Atropine for Myopia Progression (LAMP) study. Replication was conducted in 4,093 Chinese children and 1,814 Chinese adults. Four lead-SNPs (*MIR4275* rs292034, *TENM3* rs17074027, *LOC101928911* rs6925312 and *FAM135B* rs4609227) showed genome-wide significant association (*P* ≤ 5.0 × 10^−8^) with SE. *TENM3* had been associated with myopia in adults before, whilst the other three loci, *MIR4275*, *LOC101928911* and *FAM135B*, were novel. Significant interaction between genetic risk scores (GRS) and near work on SE was also detected (β_interaction_ = 0.14, *P*_interaction_ = 0.0003). This study identified novel genetic loci for children refractive error and suggested myopia intervention can be individualized based on the genetic risk of children.

## Introduction

Myopia is a mismatch between ocular optical power and axial length (AL) of the eye, leading to blurred distant images without proper optical correction. High myopia (HM), usually defined by a spherical equivalent (SE) of less than -6 diopter (D), confers high risk to sight-threatening complications, such as retinal detachment, myopic macular degeneration, and glaucoma^1^. It is predicted that half and one-tenth of the global population will be suffering from myopia and HM, respectively, by 2050^2^, and the prevalence is notably higher in East Asian populations, with up to 80–90% of high school graduates being myopic and nearly 20% of them highly myopic^3^. Therefore, it is imperative to identify individuals who are genetically predisposed to myopia to provide them with personalized interventions.

Myopia is a multifactorial disorder resulting from the interaction of multiple environmental (e.g., excessive near work and insufficient outdoor activities) and genetic risk factors. To date, over 400 genetic loci have been identified for refractive error in genome-wide association study (GWAS) of adults^4,5^. However, many single-nucleotide polymorphisms (SNPs) associated with myopia in adults cannot be replicated in child cohorts, and some SNPs showed heterogeneous, or even opposite, effects in children^6,7^. Therefore, there is still a need of identifying genes and SNPs that are associated with refractive error, especially myopia, in children, a stage of life when most of myopia starts to develop.

Certain environmental factors, such as educational pressure and lifestyles, may contribute to the disparities of myopia prevalence among populations^8^. Interestingly, Europeans with higher levels of education have a significantly lower prevalence of myopia compared to East Asians^9,10^, suggesting a potential genetic predisposition influenced by ethnicity. Moreover, the gene-environment interaction (G×E) in myopia displays a complex pattern. For instance, interactions between genes and education may only become evident at higher levels of education or within specific populations^11,12^. Similarly, some genes may interact predominantly with near work, while others are more influenced by outdoor activities^6,13^. Given the above discrepancies in the genetic components of myopia between environmental factors, and among different ethnicities^7,14^, we conducted a GWAS in children, with an aim to identify new genetic loci and their interaction with environmental risk factors for myopia development early in life. We also compared the newly identified myopia loci between children and adults, with a view to confirming the specificity of certain genes in children.

## Results

### Genetic association between SNPs and refractive error in children

**Fig. 1:**
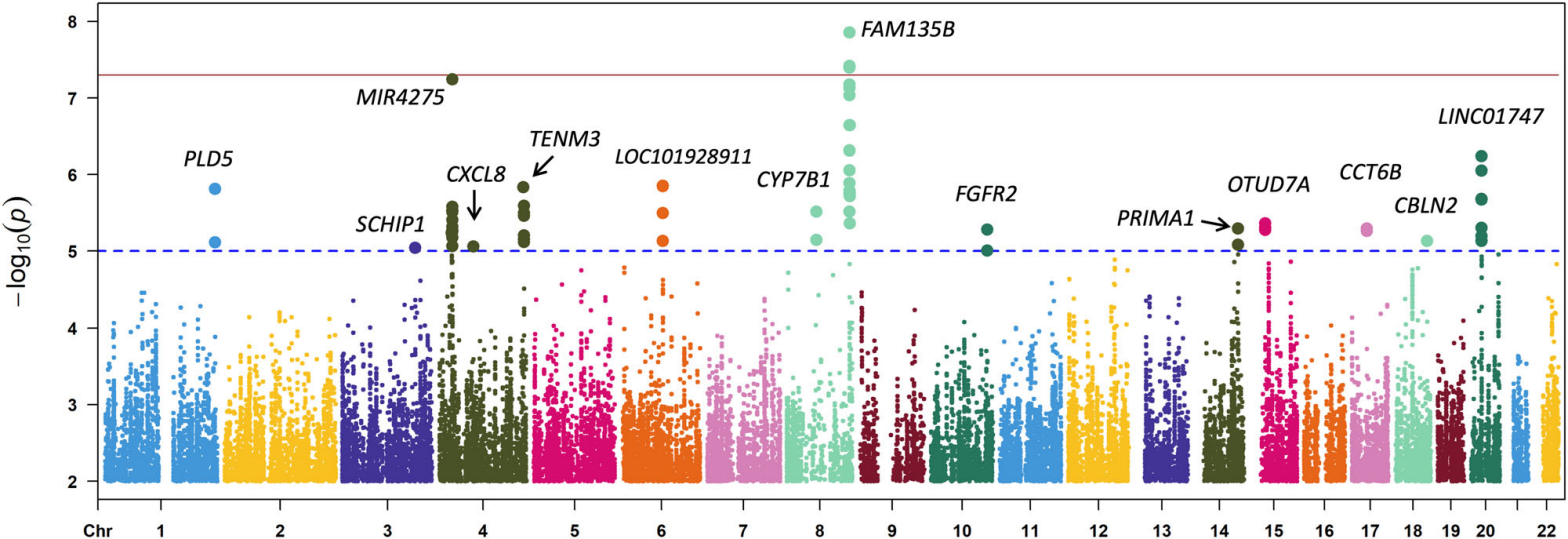
Meta-analysis results for genome-wide association to spherical equivalent in discovery stage. The y axis is –log10 *P* values for association with spherical equivalent, the x axis is chromosomes and base pair positions based on human genome build 37. The horizontal red and blue lines indicate genome-wide significance (*P* ≤ 5.0 × 10^−8^) and candidate significance (*P* ≤ 1.0 × 10^−5^), respectively. The figure was generated using R package (v. 3.4.2).

In the discovery phase, we conducted a meta-GWAS on SE using HKCES-1 (*N* = 864) and LAMP (*N* = 373). The λ was 1.003, suggesting minimal population stratification (Fig. S1). Fourteen loci passed the threshold of suggestive association (*P* ≤ 1.0 × 10^−5^, Table S1), with rs4609227 on chromosome 8q24.23 (913 kb downstream of *FAM135B*, Fig. S2a) achieving genome-wide significance (β = -1.01, *P* = 1.40 × 10^−8^; Fig. 1 and Table 1).

**Table 1:** SNPs of genome-wide significant association with spherical equivalent in children

Chr	SNP	Nearest gene		Discovery phase								Replication phase								
			EA^a^	HKCES-1 (*N* = 864)			LAMP (*N* = 373)			Meta-GWAS (*N* = 1237)		HKCES-2 (*N* = 2066)			SMS (*N* = 2027)			All cohorts (*N* = 5330)		
				EAF	β	P	EAF	β	*P*	β	*P*	EAF	β	*P*	EAF	β	*P*	β	*P*	Q test^b^
4	rs292034	*MIR4275*	G	0.013	-1.47	6.62 × 10^−7^	0.011	-1.34	3.69 × 10^−2^	-1.45	5.72 × 10^−8^	0.011	-0.63	1.40 × 10^−3^	0.013	-0.54	0.031	-0.81	1.42 × 10^−9^	0.05
4	rs17074027	*TENM3*	G	0.058	-0.63	7.66 × 10^−6^	0.06	-0.49	7.89 × 10^−2^	-0.60	1.46 × 10^−6^	0.076	-0.31	6.62 × 10^−5^	0.073	-0.30	6.20 × 10^−3^	-0.36	8.41 × 10^−11^	0.19
6	rs6925312	*LOC101928911*	G	0.77	-0.32	1.42 × 10^−5^	0.75	-0.33	0.039	-0.32	1.41 × 10^−6^	0.78	-0.16	4.03 × 10^−4^	0.79	-0.19	0.01	-0.21	5.50 × 10^−10^	0.27
8	rs4609227	*FAM135B*	C	0.025	-1.02	2.53 × 10^−7^	0.025	-0.96	2.35×10^−2^	-1.01	1.40 × 10^−8^	0.025	-0.63	2.37 × 10^−7^	0.037	-0.02	0.91	-0.50	9.44 × 10^−10^	0.025

*Chr* chromosome, *SNP* single nucleotide polymorphism, *BP* base pair position, *EA* effect allele, *EAF* effect allele frequency.

^a^EA refers to the allele negatively associated with spherical equivalent.

^b^Q test is used for heterogeneity test, Q < 0.01 indicates statistically significant heterogeneities.

In replication, 14 lead SNPs from the discovery phase were investigated in the independent HKCES-2 (*N* = 2066) and SMS (*N* = 2027) cohorts. Meta-analysis of the combined datasets (*N* = 5330) confirmed significant association of *FAM135B* rs4609227 with SE (β = -0.50, *P* = 9.44 × 10^−10^; Table 1). Another 3 SNPs also achieved genome-wide significance: *MIR4275* rs292034 in 4p15.1 (β = -0.81, *P* = 1.42 × 10^−9^), *TENM3* rs17074027 in 4q34.3 (β = -0.36, *P* = 8.41 × 10^−11^), and *LOC101928911* rs6925312 in 6q15 (β = -0.21, *P* = 5.50 × 10^−10^; Table 1 and Fig. S2b-S2d). The effect allele frequencies (EAF) of these SNPs were similar (Table S2). Notably, *FAM135B* rs4609227 also showed a borderline association with AL (*P* = 5.03 × 10^−8^), while the other three SNPs showed nominal associations (*P* ≤ 1 × 10^−5^; Table S3).

We further examined the associations of these 4 SNPs with myopia severity, comparing emmetropia vs. hyperopia, myopia vs. non-myopia (NM), mild myopia vs. NM, and moderate to high myopia (HM) vs. NM across all cohorts except LAMP (which did not include hyperopia and emmetropia cases). The odds ratios (ORs) were consistently greater than 1.0 (Table S4-S7).

In adult cohorts (Table S8 and Fig. S3), *TENM3* rs17074027 (β = -0.50, *P* = 0.0083) and *FAM135B* rs4609227 (β = -0.87, *P* = 0.0051) were consistently correlated with SE, while *MIR4275* rs292034 and *LOC101928911* rs6925312 were not, suggesting genetic differences in refractive error between children and adults. However, no statistically significant SNP×Age effect was detected for any of the four loci (Table S9-S10).

When comparing our findings with previously identified SNPs associated with myopia in European adults, we found that only a few SNPs showed consistent effects in our child cohorts (Table S11, Fig. S4). Furthermore, the frequency distribution of the four significant SNPs differed notably from those in the 1000 Genome Project across various ethnicities (Table S12), highlighting the potential impact of population-specific genetic variations on the observed associations.

### The effects of GRS and environmental factors on myopia

**Fig. 2:**
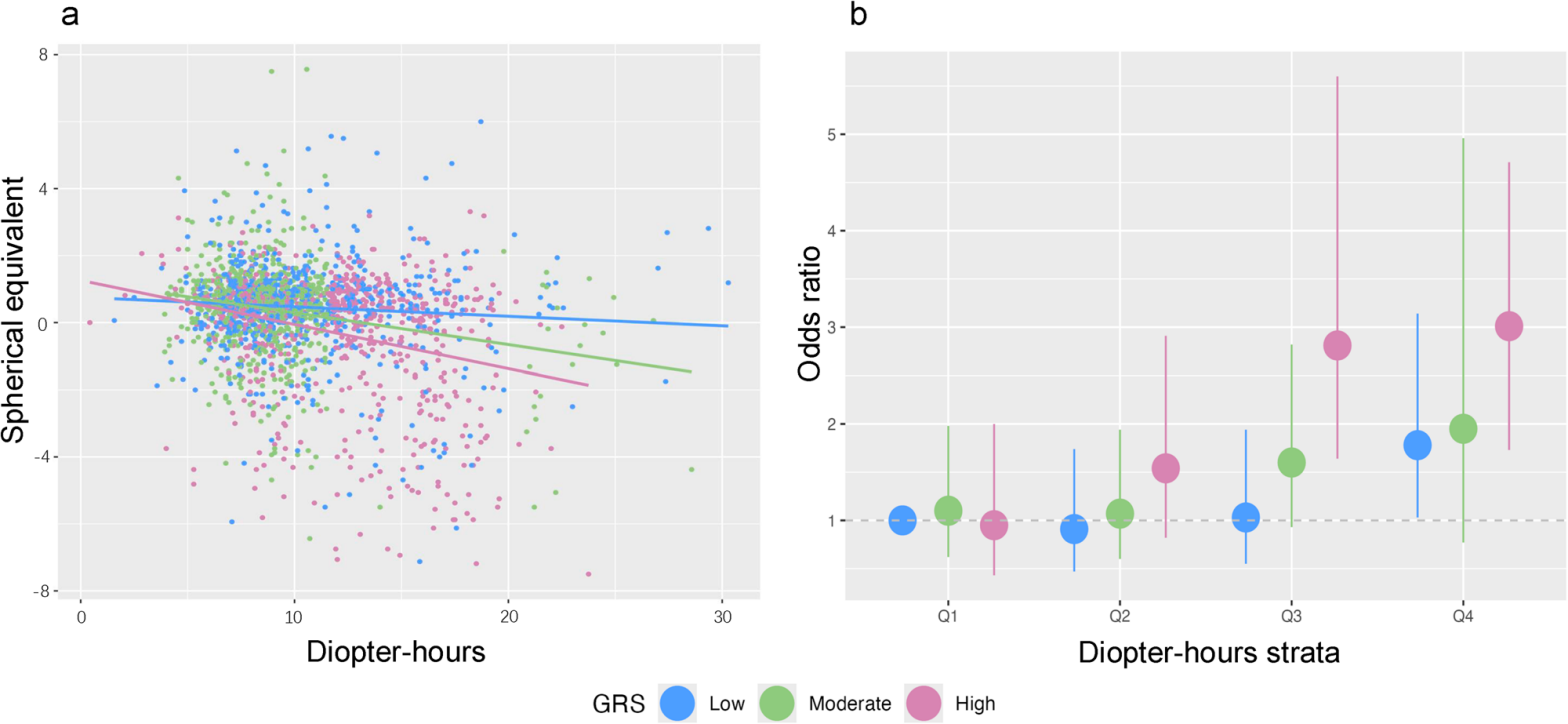
The effects of GRS and diopter-hours on myopia. **a** Interactions between GRS and diopter-hours on SE. The scatter plot represents estimated individuals’ daily diopter-hours and their corresponding SE. Each line represents the linear regression between diopter hours and SE for each stratum of GRS. **b** The odds ratio of myopia across strata of diopter-hours and GRS. Each point represents an odds ratio estimate and the vertical lines indicating confidence intervals. The blue, green and pink labels indicate low, moderate, and high GRS strata, respectively. The dashed horizontal line at 1.0 represents the null effect. GRS, genetic risk score; SE, spherical equivalent. The figure was generated using R package (v. 3.4.2).

We explored the interaction between GRS, defined by the 4 newly-identified SNPs and near work (measured by diopter-hours) and outdoor time in HKCES-2. Higher GRS and more near work time correlated with greater myopia severity (β = 1.02, *P* = 2.14 × 10^−15^ and β = -0.032, *P* = 3.49 × 10^−5^; Fig. 2a and Table S13). Notably, there was a significant interaction between GRS and near work on SE (β_interaction_ = 0.14, P_interaction_ = 0.0003; Fig. 2a and Table S13). This result was further supported by the trend test for GRS and diopter-hours strata in relation to myopia: as diopter hours increased, the risk of myopia also increased with higher GRS (Fig. 2b and Table S14). Compared to children with the lowest GRS and Q1 diopter-hour, those with the lowest GRS but exposed to >12 diopter-hours (Q4) had 1.78-fold risk of developing myopia (*P* = 0.04). However, this threshold decreased to 9 diopter-hours for those with the highest GRS (OR = 2.81, *P* = 0.00044; Fig. 2b and Table S14). Children with the highest GRS and >12 diopter-hours/day had a 3.01-fold of increased risk of myopia (*P* = 4.7 × 10^−5^; Fig. 2b and Table S14).

Being consistent with a previous study^6^, outdoor time was positively associated with SE (β = 0.23, *P* = 1.76 × 10^−6^), but no interaction with GRS was found (Table S13).

### Functional prediction

Functions of the 4 newly-identified SNPs for SE were predicted to affect epigenetic patterns and/or transcription factor (Table S15). In the GO analysis, we included the adjacent genes of the non-coding loci *MIR4275* (*PCDH7*, 1.9 Mbp downstream), *LOC101928911* (*AKIRIN2*, 82.6 Kbp upstream and *SPACA1*, 136 Kbp downstream), along with *TENM3* (*DCTD*, 87Kbp downstream) and *FAM135B*. Four significant GO terms were identified (Fig. S5 and Table S16), linking *TENM3* and *PCDH7* to processes like cell adhesion (q = 0.00073) and plasma membrane molecules (q = 0.015). *TENM3*, *DCTD* and *AKIRIN2* were associated with identical protein binding (q = 0.017), while *SPACA1* and *PCDH7* with secretory granule membrane (q = 0.024). These genes are highly expressed in posterior segment ocular tissues (Fig. S6). These results suggested that the SNPs may affect myopia development in children by regulating gene expression and cell behaviors in ocular posterior segment.

Although no significant gene-based associations were identified (Fig. S7), the causal posterior probabilities for rs292034, rs17074027, rs6925312 and rs4609227 were the highest among the linked SNPs (Table S17). Only *MIR4275* rs292034 showed a significant eQTL association, linking the region to the expression of *RP11-123O22.1*, a long non-coding RNA gene mainly expressed in brain tissues (Table S18).

## Discussion

In this study, we identified 4 loci significantly associated with SE and nominally with AL in children. Among them, *TENM3* had been associated with myopia and refractive error in adults^4^, while the other 3 loci, *MIR4275*, *LOC101928911* and *FAM135B*, were novel. Children with higher GRS defined by the lead SNPs in these 4 loci experienced a more pronounced decrease in SE compared to those with lower GRS given an equivalent amount of near work; and the severity of myopia increased with both near work and GRS levels. Children with high GRS spending >12 diopter-hours daily had a 3.01-fold risk of being myopic when compared to children with low GRS and spending <7 diopter-hours/day.

Few studies have explored age-related gene function in myopia. Some loci showed peak effects at specific ages, illustrating the complexity of gene action^7^. In a cohort of 1,160 children and adolescents (ages 2–17), *IGF1* rs5742714 was linked to myopia in children under 6, while *FGF10* rs339501 was associated with moderate and mild myopia in children aged 6–12^15^. In our study, 4 SNPs were associated with SE in children, with *TENM3* rs17074027 and *FAM135B* rs4609227 also showing stronger effects in adults. No SNP×Age interaction was found, likely due to the narrow age range in the HKCES (6–9 years) and LAMP (4–12 years, with 93% aged 6–10) cohorts. Compared to the largest GWAS of European adults^4^, only a few SNPs were nominally associated with refractive error in our child cohorts, with 20.9% showed consistent effects (Fig. S4). This may reflect population allele frequency differences and nonlinear age-related SE change in children. This raises the question of whether the loci involved in early-onset myopia also affect final myopia status, as seen in atropine treatment, where short-term use did alter final refractive error over 10–20 years^16^. If genes for early myopia onset do not influence final refractive status, distinct mechanisms may underlie childhood myopia development and progression. Future interventions may need to target different genes or pathways, which remain to be explored.

This study identified significant interactions of myopia GRS with diopter-hours, but not with outdoor time, aligning with the study of Fan et al.^6^. In adults, G×E analysis showed stronger genetic effects with higher education levels, with ethnic-specific interactions^11,17^. For example, *AREG*, *GABRR1* and *PDE10A* interacted with education in Asians but not in Europeans^12^. A few studies also reported loci interacted with outdoor time and other environmental factors^13,18^. Further studies are needed to elucidate the genetic and environmental contributions to early-onset myopia and long-term refractive stability.

The functions of *MIR4275* rs292034, *LOC101928911* rs6925312 and *FAM135B* rs4609227 are not well understood due to a lack of eQTL data, despite their statistical association with educational attainment^19,20^. Noncoding RNAs play roles in chromatin organization and gene expression by modulating histone modifications and interacting with transcription factors^21^. *MIR4275* has been linked to astigmatism, possibly via *PCDH7*^22^. In our study, *MIR4275* rs292034 was associated with both spherical power and corneal astigmatism (Table S19-20), suggesting shared genetic factors. *FAM135B*, involved in the AKT/mTOR signaling pathway, affects scleral extracellular matrix (ECM) remodeling and mainly expressed in the posterior segment of the eye (Fig. S6f). *TENM3*, a member of the teneurin family, is primarily expressed in the central nervous system and functions as cell adhesion molecules^23^. Mutations in *TENM3* have been linked to congenital ocular diseases, such as ocular coloboma, microphthalmia, congenital cataract^24,25^, and oval cornea^25^. In European adults, *TENM3* rs35446926 linked to myopia and refractive error (*P* = 5.6 × 10^−11^)^4^. In our study, In our study, *TENM3* rs17074027 was significantly associated with SE in children and had a stronger effect in adults. Interestingly, *TENM4*, a paralog of *TENM3*, was associated with early onset of HM^26^. These findings suggested that *TENM3* influences myopia development from an early age and the effect continues into adulthood.

This is the first GWAS targeting myopia in children, using cycloplegic refraction to minimize accommodation bias. Studying children with less environmental exposure helps identify genetic contributors to myopia. Rapid eye growth in childhood reveals gene-environment interactions. Combining two independent cohorts (population-based and RCT-based) for a meta-GWAS increased sample diversity and result robustness. Significant SNP associations in both HKCES and LAMP cohorts suggest these genetic markers are robustly linked to myopia. However, only lead SNPs from four loci were detected, which may not cover each genetic locus sufficiently. Additionally, the study was geographically confined to southern Chinese subjects from Hong Kong and Shantou, necessitating further replication in diverse populations.

Despite limitations, this study provided important clinical implications, showing that myopia risk increases with GRS levels during intensive near work. Children with high GRS should limit near work to under 9 diopter-hours/day. All children should avoid exceeding 12 diopter-hours of near work daily to reduce myopia risk.

## Methods

### General study design and subjects

A 3-stage GWAS was adopted to identify and validate new gene loci for myopia (Fig. S8). In Stage 1, a meta-GWAS analysis was conducted on two independent children GWAS datasets: the Hong Kong Children Eye Study (HKCES-1) and Low Concentration Atropine for Myopia Progression (LAMP) Study, using baseline SE data collected before any treatment^27,28^. In stage 2, SNPs passing the threshold (*P* ≤ 1 × 10^−5^) were replicated in two additional children cohorts: HKCES-2 and Shantou Myopia Study (SMS)^29,30^, with a total of 5,330 children. In Stage 3, SNPs reaching genome-wide significance (*P* ≤ 5 × 10^−8^) were validated in two independent adult cohorts. Adult-1 is a population-based cohort recruited from 2015 to 2018^31^, and Adult-2 is a myopia cohort recruited from 2016 to 2017. Detailed description and demographics of each cohort are provided in Table S21-S22. All studies conducted in Hong Kong were approved by the Ethics Committee of the Chinese University of Hong Kong. The LAMP Study was registered with the Centre for Clinical Research and Biostatistics Clinical Trials Registry at the Chinese University of Hong Kong (CUHK_CCT00383). The SMS study was approved by the Human Medical Ethics Committee of the Joint Shantou International Eye Center (ID: EC20200120(1)-P15). All procedures were conducted conforming to the tenets of the Declaration of Helsinki. Informed consent was obtained from all individual participants or their legal guardian included in the study.

### Ocular examinations

Cycloplegic autorefraction of each HKCES and LAMP Study subject was performed using an autorefractor (Nidek ARK-510A) following a detailed cycloplegia regimen. This regimen included two cycles of eye drops: cyclopentolate 1% (Cyclogyl, Alcon-Couvreur, Rijksweg, Belgium) and tropicamide 1% (Santen, Osaka, Japan), administered to both eyes 10 min apart. If the pupillary light reflex was still present, a third cycle was given 30 min after the second^27^. Ocular AL was measured using a Zeiss IOL Master (Carl Zeiss Meditec Inc, Dublin, CA), based on noncontact partial coherence interferometry^28,32^. Children in the SMS underwent a similar cycloplegia procedure with cyclopentolate 1% (Cyclogel; Alcon Laboratories, Fort Worth, TX) and tropicamide 1% (Mydriacyl; Alcon Laboratories), followed by autorefraction using an RK-F1 Refractometer/Keratometer (Canon, Inc., Tochigi, Japan)^33^. Adult refraction measurements followed the same procedure as in Hong Kong, but without cycloplegia ^31^.

### Environmental factors

The detailed methods of validated questionnaires, calculation of outdoor exposure and near-work time had been conducted in the HKCES^34^. In brief, diopter-hour is used as proxy for near-work time, calculated using the following equation: [(hours spent studying + hours spent reading for pleasure) × 3] + [(hours spent playing video games or working on the computer at home) × 2] + [(hours spent watching television) × 1]. Outdoor time is the total time spent on leisure activities and sports^34^. The diopter-hour was categorized into four strata: Q1 ( < 7 diopter-hours/day), Q2 (7–9 diopter-hours/day), Q3 (9–12 diopter-hours/day), and Q4 ( > 12 diopter-hours/day).

### DNA extraction, genotyping, imputation, quality controls and genetic risk score

Genomic DNA of subjects for the two discovery GWAS cohorts and two adult cohorts were extracted from venous blood, whilst DNA of the HKCES-2 and SMS children were extracted from buccal swab or blood samples. The details of DNA extraction have been reported elsewhere^27,30,31^. Genotyping of the discovery samples was performed using the Illumina Infinium Asian Screening Array (Illumina Inc., San Diego, California, USA). Participants were excluded if the genotyping success rate was <95%. SNPs with a call rate of <98%, deviating from Hardy-Weinberg equilibrium (*P* < 10^−6^), and/or minor allele frequency (MAF) of <0.01 were excluded. Genotype imputation was conducted in the Michigan imputation server (https:// imputationserver. sph.umich. edu/index. html#!pages/home) using the reference panel of 1000 Genomes Project Phase 3 V5 (GRCh37/hg19), Eagle V.2.4 (phasing), and the EAS population. The imputation quality score r^2^ was ≥ 0.8, and total genotyping rate was 1. After quality control, 5,108,499 SNPs were eligible for the meta-GWAS analysis. Genotyping of the replication samples was conducted using TaqMan SNP Genotyping Assays (Applied Biosystems, Foster City, CA) on a Light Cycler 480 Real-Time PCR System (Roche Diagnostics, Basel, Switzerland).

### Statistical Analysis

Data analysis of the two discovery GWAS datasets was carried out under an additive model in Plink V.1.9. The first 5 and first 7 principal components in HKCES and LMAP study, respectively, were adjusted in linear regression to control population stratification according to the results of Tracy-Widom test (Table S23)^35^, along with age and sex or additional age^2^ adjustment considering the nonlinear refractive error development during childhood^36^. To calculate the genomic inflation factor (λ), we first computed the chi-squared statistic for each SNP and determined the median of these values. λ was then calculated by dividing this median by the expected median from a chi-squared distribution with 1 degree of freedom. Inverse Variance Weighted (IVW) method was applied to meta-analyze the two GWAS datasets and later the replication datasets; a fixed-effect model was applied if the Cochran’s Q statistic showed a *P* ≥ 0.01; otherwise, a random-effect model ^37^.

Multivariable logistic model was used to evaluate the association between significant SNPs and different myopia severities. Genetic risk score (GRS) was calculated as the sum of the risk alleles weighted by their effect sizes, using the formula:

$${\rm{GRS}}=\mathop{\sum }\limits_{{\rm{i}}=1}^{{\rm{n}}}{\rm{\beta }}{\rm{i}}\times {\rm{SNPi}}1$$

where SNPi is the genotype (0, 1 or 2 for the number of risk alleles), βi is the effect size. The histogram in Fig. S9 shows the distribution of GRS. Multivariable linear regression was used to assess the marginal effects of GRS on SE and the interaction between GRS and diopter-hours on SE. Multivariable logistic regression was applied to examine the association of GRS strata and diopter-hours with myopia status. A trend test was performed to assess the significance of the GRS effects across diopter-hour strata. All models were adjusted for age, sex and other relevant confounders when appliable in the R package (v. 3.4.2).

### Functional Annotation

Regional association plots for target regions were generated using LocusZoom version 1.4 (https://genome.sph.umich.edu/wiki/LocusZoom_Standalone)^38^. SNPnexus (https://www.snp-nexus.org/v4/) was used for functional annotation^39^, and Human Eye Transcriptome Atlas (https://www.eye-transcriptome.com/index.php) for gene expresson^40^. Gene Ontology (GO) analysis was performed using ConsensusPathDB (http://cpdb.molgen.mpg.de/), with candidate genes selected from the nearby coding gene(s) within 1000Kbp of the lead SNP^41^. *P* < 0.01 was applied for enriched GO terms, and the false discovery rate (FDR) was used for multiple comparisons^42^. A q value of < 0.05 was considered significant^42^. ClueGo plugin in Cytoscape software (ver. 3.9.1) was used for visualizing the enriched GO maps with q < 0.05. Gene-based test and expression quantitative trait locus (eQTL) analysis were performed using FUMA^10^. *P* < 2.60 × 10^−6^ was considered significant for gene-based test after Bonferroni correction for 19198 protein-coding genes. For eQTL analysis, *P* < 0.05 after FDR correction was considered significant. The CAVIAR tool was used to identify a credible set of SNPs within a 500Kbp window and with *r*^2^ > 0.2 of the lead SNPs in this study ^43^.

## Supplementary information

Supplementary Information

## Data Availability

The complete GWAS summary data can be found in the GWAS Catalog (accession number GCST90566418). Raw genotype data are available for collaborative research under restricted conditions to ensure participant privacy. For data access inquiries, please contact the corresponding authors, J.C.Y. (yamcheuksing@cuhk.edu.hk) and L.J.C. (lijia_chen@cuhk.edu.hk).

## Code availability

Analyses were carried out using open-source published pieces of software which are referenced in the manuscript.
