# Supplementary material for: Genome-wide association study identified novel loci and gene-environment interaction for refractive error in children
Source: NPJ Genom Med. 2025 May 23;10:44. doi: 10.1038/s41525-025-00504-5 (PMC12102314; doi:10.1038/s41525-025-00504-5)
Supplement: Supplementary file 1 — Supplementary Information [file 41525_2025_504_MOESM1_ESM.pdf]

| List of supplementary materials | Title |
|---|---|
| Supplementary Figure 1 | Fig. S1. Quantile-quantile plot of the association results of HKCES-1 and LAMP in the discovery stage |
| Supplementary Figure 2 | Fig. S2. Regional association plots of the 4 loci associated with spherical equivalent. |
| Supplementary Figure 3 | Fig. S3. Forest plot comparing associations between SNPs and spherical equivalent in children versus in adults |
| Supplementary Figure 4 | Fig. S4. Comparison of significant myopia loci between genome-wide association studies of European adults and our Chinese children |
| Supplementary Figure 5 | Fig. S5. Enrichment map showing the Gene Ontology (GO) terms identified in this study |
| Supplementary Figure 6 | Fig. S6. Expression levels of genes nearby genome-wide significant SNPs predicted in ocular tissues by Human Eye Transcriptome Atlas |
| Supplementary Figure 7 | Fig. S7. Manhattan plot of gene-based test |
| Supplementary Figure 8 | Fig. S8. Flowchart of the 3-stage study design |
| Supplementary Figure 9 | Fig. S9. Distribution of genetic risk scores |
| Supplementary Table 1 | Table S1. Results of the meta-GWAS of HKCES-1 and LAMP in the discovery stage |
| Supplementary Table 2 | Table S2. Results of the meta-GWAS of HKCES-1 and LAMP in the discovery stage |
| Supplementary Table 3 | Table S3. Associations between axial length and 4 SNPs identified in this study |
| Supplementary Table 4 | Table S4. Association between rs292034 and different myopia severities |
| Supplementary Table 5 | Table S5. Association between rs17074027 and different myopia severities |
| Supplementary Table 6 | Table S6. Association between rs6925312 and different myopia severities |
| Supplementary Table 7 | Table S7. Association between rs4609227 and different myopia severities |
| Supplementary Table 8 | Table S8. Associations between spherical equivalent and 4 SNPs in adults |
| Supplementary Table 9 | Table S9. SNP×Age interaction analysis of 4 significant SNPs from GWAS of HKCES-1 cohort |
| Supplementary Table 10 | Table S10. SNP×Age interaction analysis of 4 significant SNPs in GWAS of LAMP cohort |
| Supplementary Table 11 | Table S11. Comparison of SNPs previously associated with myopia in European adults and their effects in our children cohorts |
| Supplementary Table 12 | Table S12. Frequency distribution in our study and the 1000Genome Project |
| Supplementary Table 13 | Table S13. Interactions between genetic risk score strata of spherical equivalent and diopter-hours and outdoor time |
| Supplementary Table 14 | Table S14. Associations of strata of genetic risk score and diopter-hours in relation to myopia status |
| Supplementary Table 15 | Table S15. Functional prediciton of 4 genome-wide significance SNPs |
| Supplementary Table 16 | Table S16. Gene Ontology Terms Significantly Enriched among Candidate Genes |
| Supplementary Table 17 | Table S17. Credible set of SNPs identified using CAVIAR |
| Supplementary Table 18 | Table S18. Significant eQTL results |
| Supplementary Table 19 | Table S19. Association between MIR4275 rs292034 and spherical power in children |
| Supplementary Table 20 | Table S20. Association between MIR4275 rs292034 and corneal astigmatism in children |
| Supplementary Table 21 | Table S21. Study cohorts description |
| Supplementary Table 22 | Table S22. Demographics of study cohorts |
| Supplementary Table 23 | Table S23. The results of Tracy-Widom test in HKCES-1 and LAMP Study for principal component adjustment |

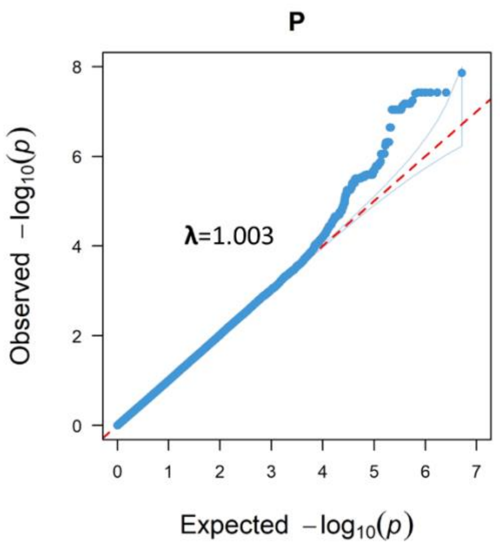


**Fig. S1. Quantile-quantile plot of the association results of HKCES-1 and LAMP in the discovery stage**. The red dashed line represents the null distribution. The observed -log10 (p-values) (blue dots) are plotted against the expected -log10 (p-values) under the null hypothesis of no association. The genomic inflation factor (λ) is 1.003, indicating little evidence of population stratification. HKCES, Hong Kong Children Eye Study; LAMP, Low Concentration Atropine for Myopia Progression Study. The figure was generated using R package (v. 3.4.2).

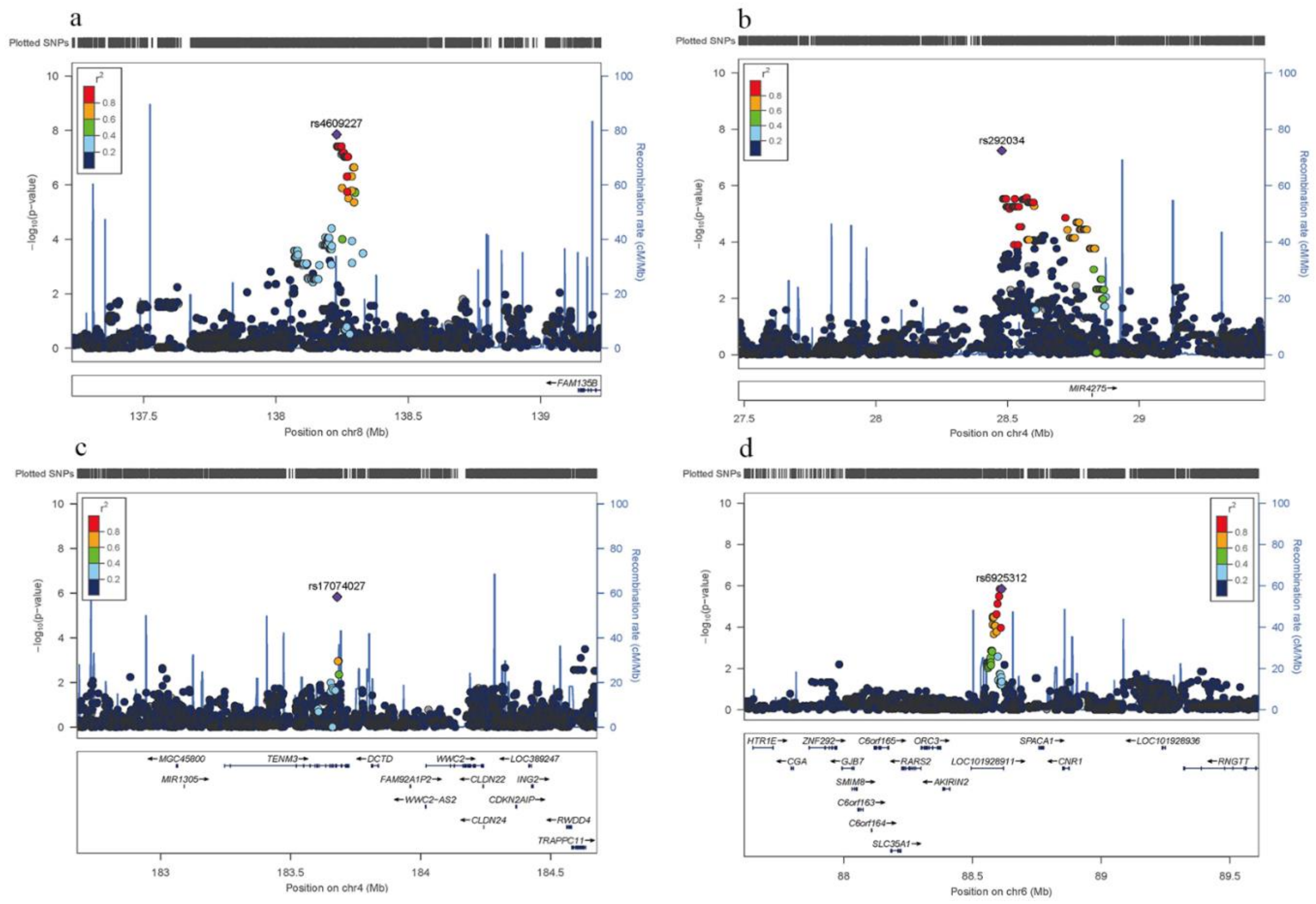


**Fig. S2. Regional association plots of the 4 loci associated with spherical equivalent**. Plots are shown for association in the Hong Kong children genome-wide association study discovery set at (a) *FAM135B* on 8q24.23; (b) *MIR4275* on 4p15.1; (c) *TENM3* on 4q34.3; (d) *LOC101928911* on 6q15. Genetic linkage disequilibrium (LD) in each region is estimated by $r^2$. The figure was generated using LocusZoom version 1.4 (https://genome.sph.umich.edu/wiki/LocusZoom_Standalone).

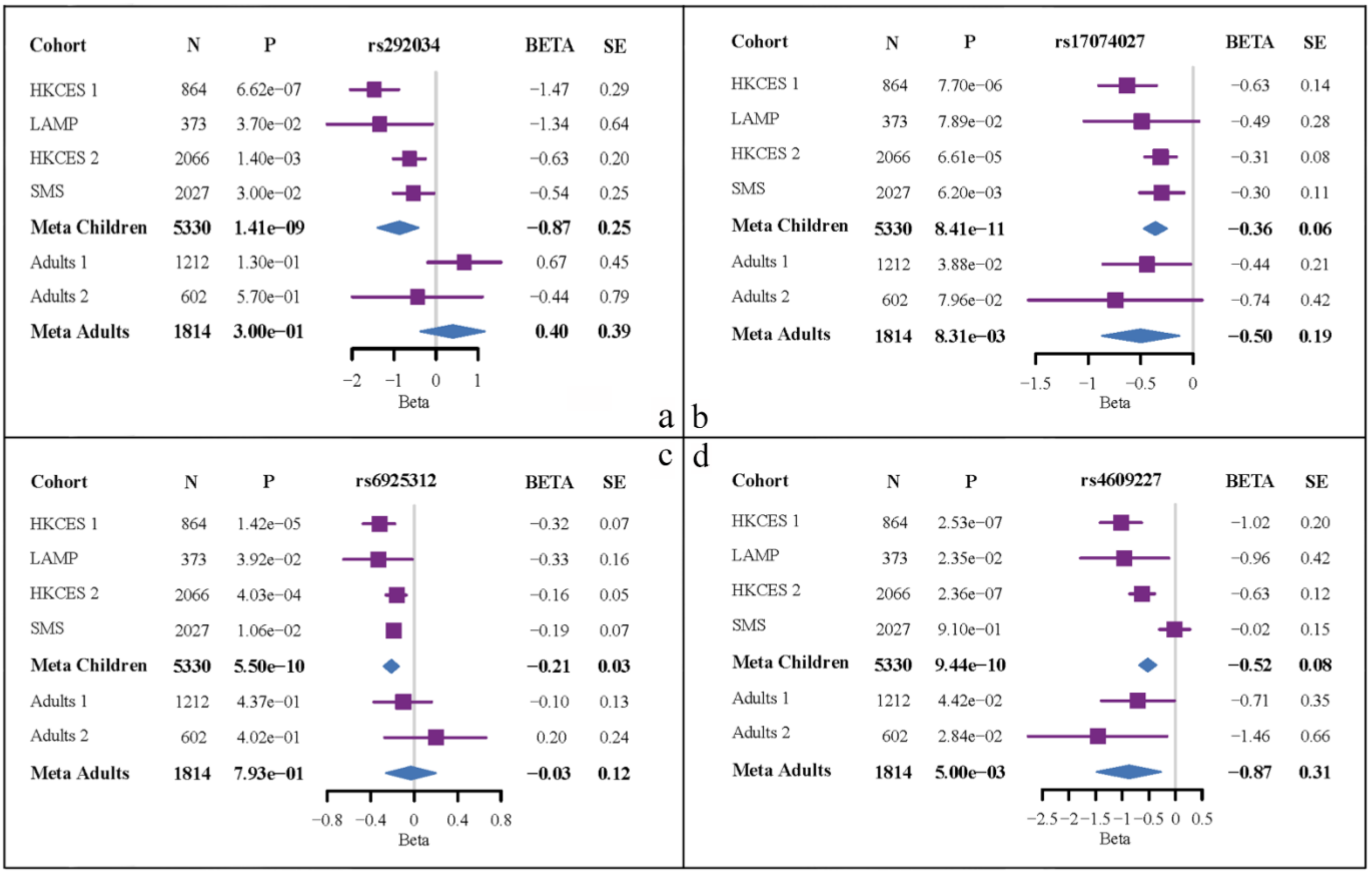


**Fig. S3. Forest plot comparing associations between SNPs and spherical equivalent in children versus in adults**. Each sub-figure represents a different SNP: (a) rs292034, (b) rs17074027, (c) rs6925312, and (d) rs4609227. The effect sizes (Beta) and standard errors (SE) are shown for each cohort. Meta-analysis was conducted using the Inverse Variance Weighted (IVW) fixed-effect model to combine the results from the different studies, with the summary effects presented for both children and adults. The horizontal lines indicate 95% confidence intervals. The figure was generated using R package (v. 3.4.2).

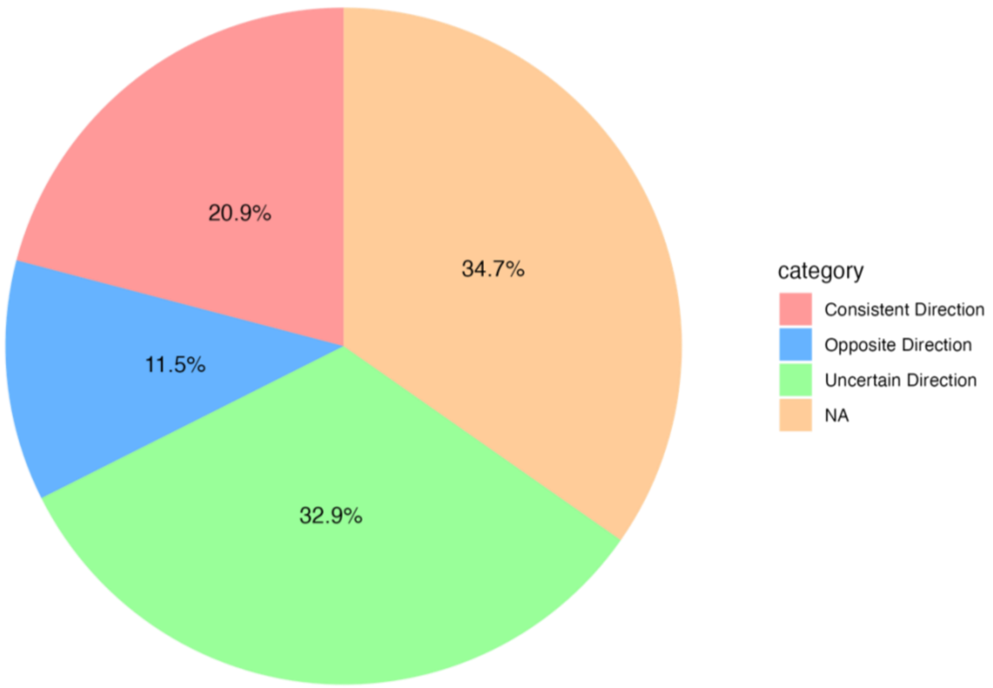


**Fig. S4. Comparison of significant myopia loci between genome-wide association studies of European adults and our Chinese children.** The orange part represents the percentage of SNPs that are not present in the GWAS of Chinese children; the red, blue, and green sections represent the percentages of SNPs showing consistent, opposite, and uncertain directions between the GWAS of European adults and Chinese children, respectively. The total number of 435 significant autosomal loci were from the study of Hysi et al. (Meta-analysis of 542,934 subjects of European ancestry identifies new genes and mechanisms predisposing to refractive error and myopia. *Nat Genet*. 2020 Apr;52(4):401-407). GWAS, genome-wide association studies; SNP, single nucleotide polymorphisms. The figure was generated using R package (v. 3.4.2).

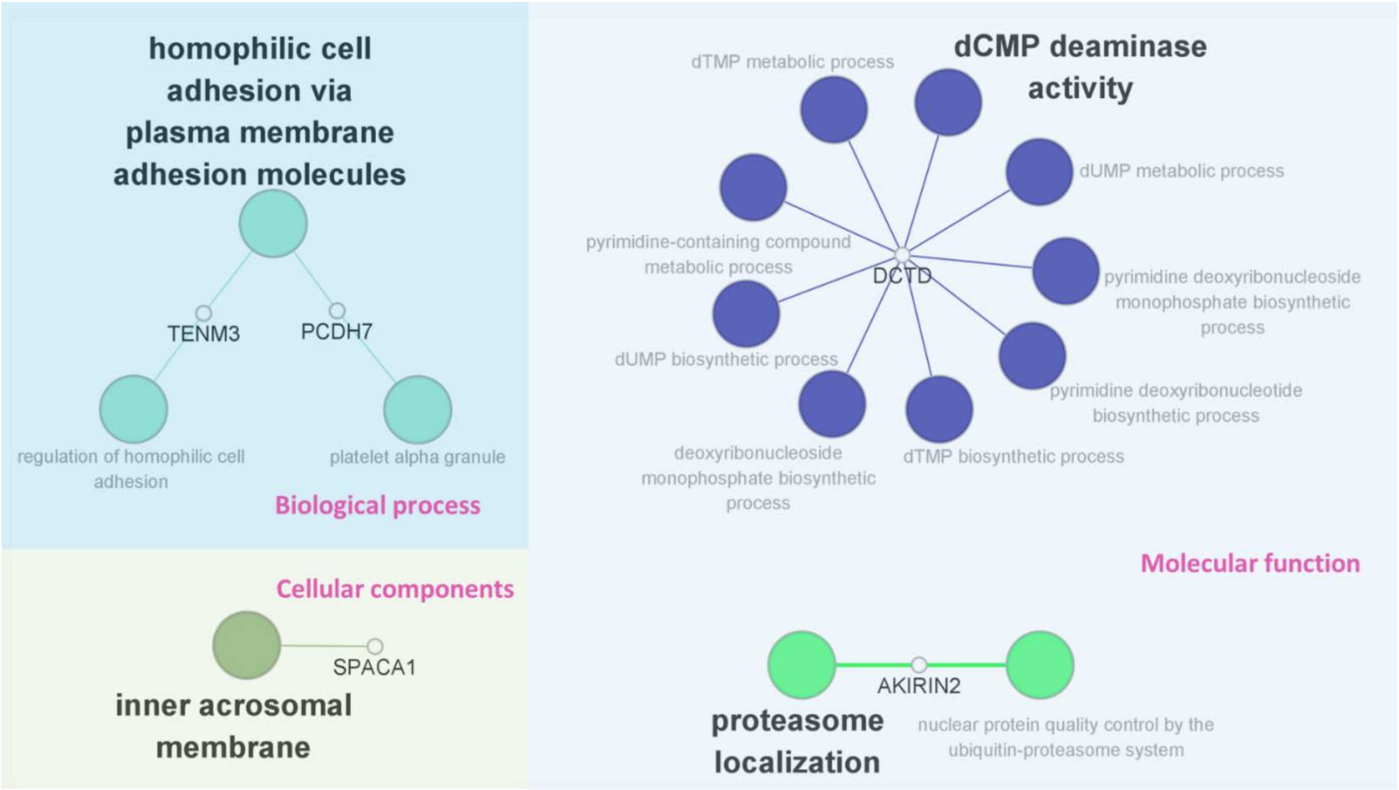


**Fig. S5. Enrichment map showing the Gene Ontology (GO) terms identified in this study**. Nodes represent GO terms. Important GO terms were shown in black bold. The map is categorized into three sections: biological processes, cellular components, and molecular functions. The figure was generated using ClueGo plugin in Cytoscape software (v. 3.9.1).

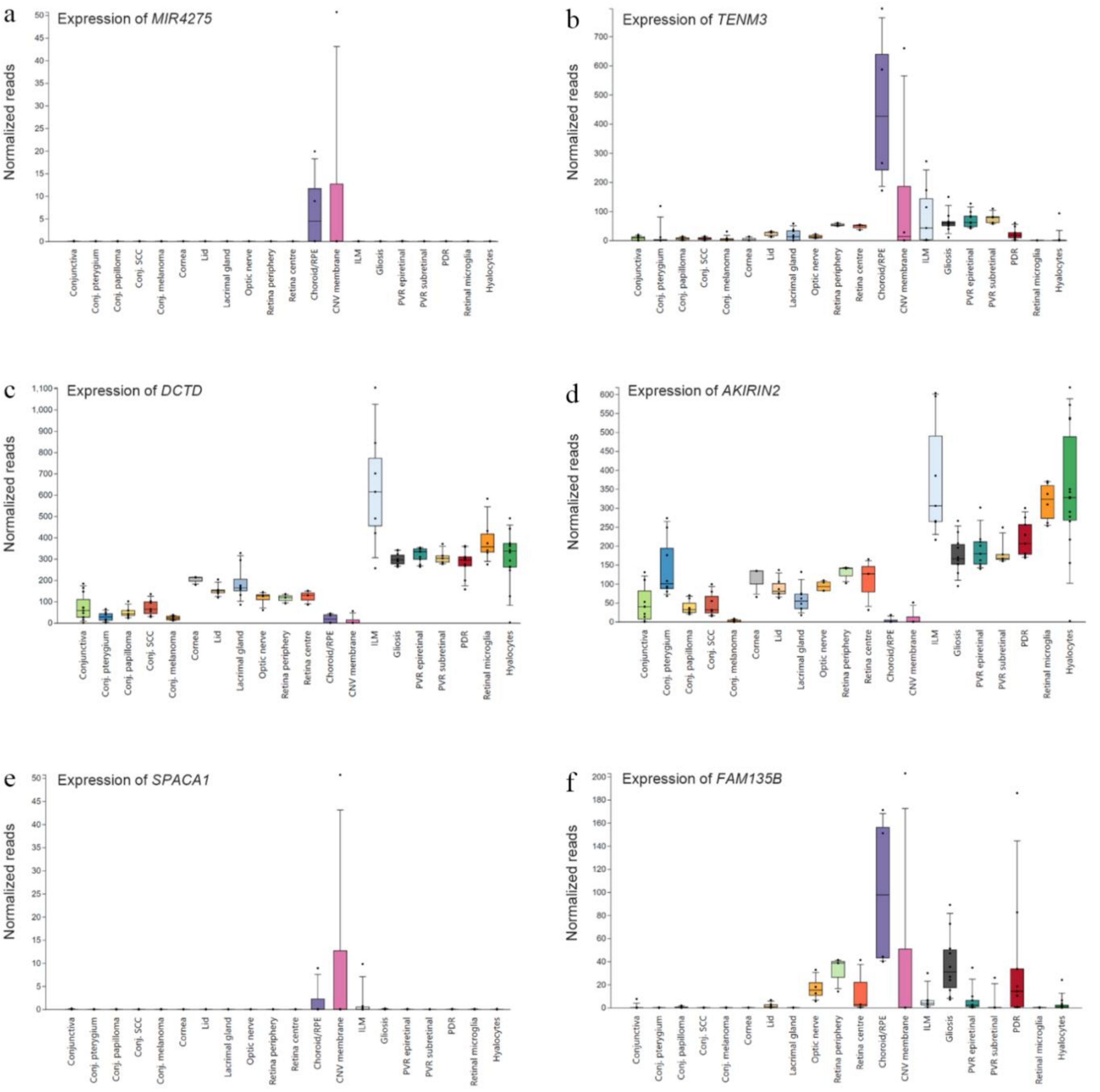


**Fig. S6. Expression levels of genes nearby genome-wide significant SNPs predicted in ocular tissues by Human Eye Transcriptome Atlas**. Normalized read counts are shown for (a) *MIR4275*, (b) *TENM3*, (c) *DCTD*, (d) *AKIRIN2*, (e) *SPACA1*, and (f) *FAM135B*. Each box plot represents the distribution of normalized reads, indicating the relative expression levels of the genes in different samples. Notable variations in expression can be observed in specific tissue or cell types for each gene. RPE, retinal pigment epithelium, ILM, internal limiting membrane; PVR, proliferative vitreoretinopathy; PDR, proliferative diabetic retinopathy. The figure was generated using Human Eye Transcriptome Atlas website (https://www.eye-transcriptome.com/index.php).

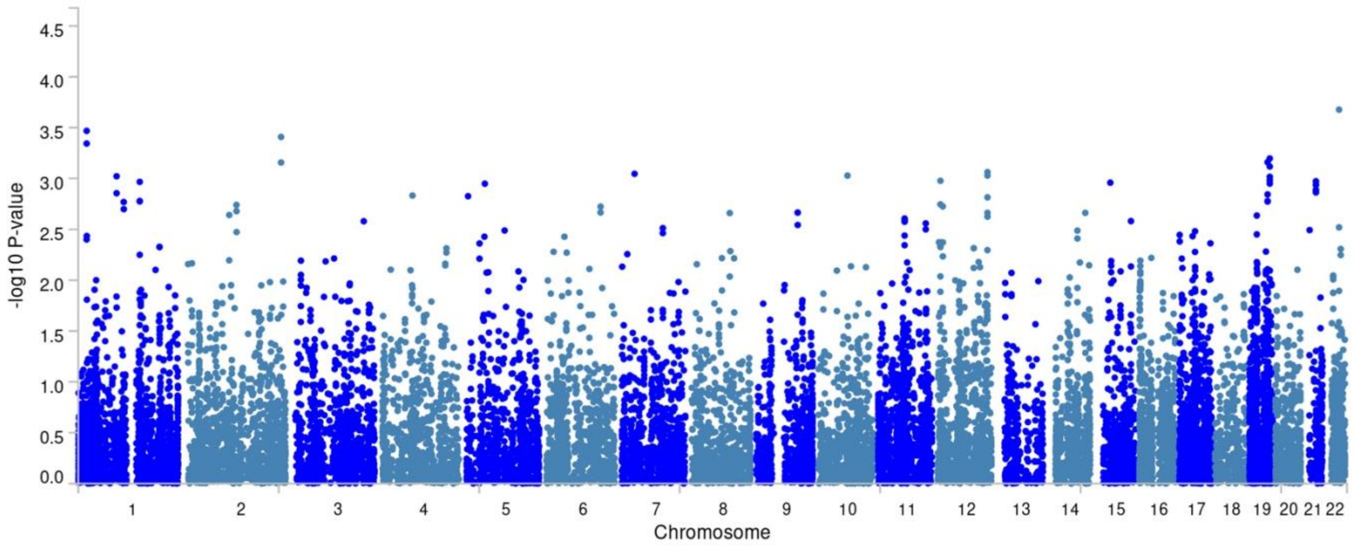


**Fig. S7. Manhattan plot of gene-based test**. The y axis is for the -log10 P values for association with spherical equivalent, the x axis is for chromosomes and base pair positions based on human genome build 37. P $<2.60\times10^{-6}$ defines statistical significance. No genes reached the threshold for statistical significance in this analysis. The figure was generated using FUMA website (https://fuma.ctglab.nl/).

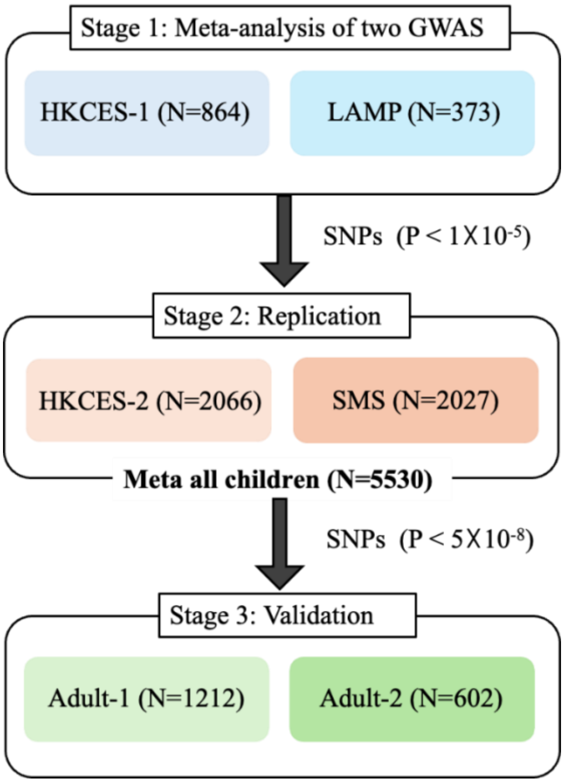


**Fig. S8. Flowchart of the 3-stage study design.** Stage 1 is a meta-analysis of two GWAS datasets from independent child cohorts: HKCES-1(N=864) and LAMP (N=373). Stage 2 is the replication of SNPs passing the preset significance threshold ($P \leq 1\times10^{-5}$) in another independent child cohorts: HKCES-2 (N=2066) and SMS (N=2027). A meta-analysis was then performed combining all child cohorts (total N=5530). Stage 3 is validation of SNPs reaching genome-wide significance ($P \leq 5\times10^{-8}$) in two independent adult cohorts: Adult-1 (N=1212) and Adult-2 (N=602). GWAS, genome-wide association studies; HKCES, Hong Kong Children Eye Study; LAMP, Low Concentration Atropine for Myopia Progression Study; SMS, Shantou Myopia Study. The figure was generated using Microsoft PowerPoint (v. 16.82).

.

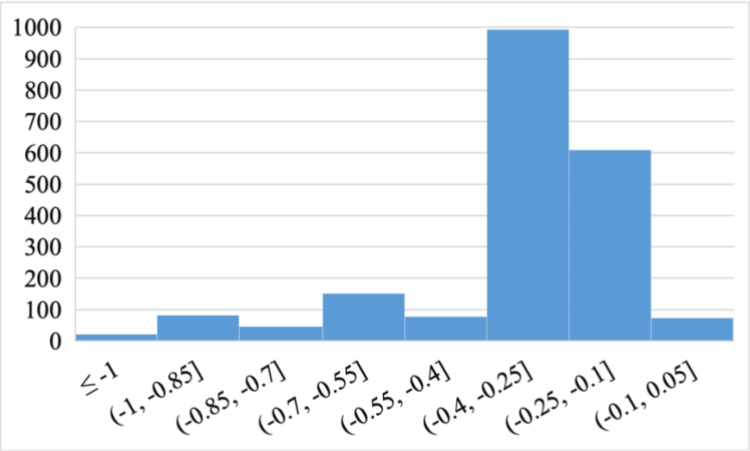


**Fig. S9. Distribution of genetic risk scores**. The x-axis represents the GRS score intervals, and the y-axis indicates the number of individuals within each interval. GRS, genetic risk score. The figure was generated using Microsoft Excel (v. 16.82).

**Table S1. Results of the meta-GWAS of HKCES-1 and LAMP in the discovery stage**

| | | | | | HKCES-1 | | | LAMP | | | Meta-GWAS with age adjusted | | | | | | | Meta-GWAS with age2 adjusted | | | | | | |
|---|---|---|---|---|---|---|---|---|---|---|---|---|---|---|---|---|---|---|---|---|---|---|---|---|
| CHR | BP | SNP | EA | NEA | EAF | BETA | P | EAF | BETA | P | EAF | BETA[a] | P[a] | BETA.R.[b] | P.R.[b] | Q test | $I^2$ (%) | BETA[a] | P[a] | BETA.R.[b] | P.R.[b] | Q test | $I^2$ (%) | Nearby loci |
| 1 | 242620371 | rs75623525 | A | G | 0.064 | -0.49 | 1.84E-04 | 0.062 | -0.89 | 1.16E-03 | 0.063 | -0.56 | 1.54E-06 | -0.62 | 8.71E-04 | 0.19 | 42.74 | -0.57 | 1.36E-06 | -0.62 | 1.18E-03 | 0.17 | 46.38 | *PLD5* |
| 3 | 159168596 | rs201346584 | C | T | 0.18 | -0.29 | 5.97E-04 | 0.15 | -0.56 | 2.00E-03 | 0.17 | -0.34 | 9.09E-06 | -0.38 | 3.13E-03 | 0.17 | 46.81 | -0.34 | 8.45E-06 | -0.38 | 4.24E-03 | 0.15 | 51.04 | *SCHIP1* |
| 4 | 28478119 | rs292034 | G | T | 0.013 | -1.47 | 6.62E-07 | 0.011 | -1.34 | 3.69E-02 | 0.012 | -1.45 | 5.72E-08 | -1.45 | 5.72E-08 | 0.85 | 0 | -1.44 | 5.91E-08 | -1.44 | 5.908E-08 | 0.83 | 0 | *MIR4275* |
| 4 | 74640294 | rs78130161 | T | C | 0.023 | -0.85 | 5.81E-05 | 0.028 | -0.75 | 6.27E-02 | 0.025 | -0.83 | 8.71E-06 | -0.83 | 8.71E-06 | 0.83 | 0 | -0.83 | 9.03E-06 | -0.83 | 0.000009032 | 0.81 | 0 | *CXCL8-CXCL6* |
| 4 | 183678700 | rs17074027 | G | T | 0.058 | -0.63 | 7.66E-06 | 0.06 | -0.49 | 7.89E-02 | 0.059 | -0.6 | 1.46E-06 | -0.6 | 1.46E-06 | 0.66 | 0 | -0.6 | 1.25E-06 | -0.6 | 0.000001249 | 0.69 | 0 | *TENM3* |
| 6 | 88612769 | rs6925312 | G | A | 0.77 | -0.32 | 1.42E-05 | 0.75 | -0.33 | 3.92E-02 | 0.76 | -0.32 | 1.41E-06 | -0.32 | 1.41E-06 | 1 | 0 | -0.32 | 1.52E-06 | -0.32 | 0.000001519 | 0.97 | 0 | *LOC101928911* |
| 8 | 65501179 | rs149726960 | T | C | 0.018 | -1.11 | 1.77E-06 | 0.02 | -0.37 | 4.37E-01 | 0.019 | -0.96 | 3.05E-06 | -0.85 | 1.55E-02 | 0.16 | 49.66 | -0.97 | 2.77E-06 | -0.85 | 0.01535 | 0.16 | 50 | *CYP7B1* |
| 8 | 138229001 | rs4609227 | C | G | 0.025 | -1.02 | 2.53E-07 | 0.025 | -0.96 | 2.35E-02 | 0.025 | -1.01 | 1.40E-08 | -1.01 | 1.40E-08 | 0.89 | 0 | -1 | 1.58E-08 | -1 | 1.584E-08 | 0.87 | 0 | *FAM135B* |
| 10 | 123023728 | rs117198263 | T | C | 0.039 | -0.72 | 1.68E-05 | 0.034 | -0.55 | 1.39E-01 | 0.037 | -0.69 | 5.22E-06 | -0.69 | 5.22E-06 | 0.67 | 0 | -0.7 | 4.83E-06 | -0.7 | 0.000004833 | 0.66 | 0 | *FGFR2* |
| 14 | 94279274 | rs75782951 | T | A | 0.033 | 0.77 | 9.67E-06 | 0.027 | 0.48 | 2.42E-01 | 0.031 | 0.72 | 5.07E-06 | 0.72 | 5.07E-06 | 0.53 | 0 | 0.73 | 4.82E-06 | 0.73 | 0.000004817 | 0.55 | 0 | *PRIMA1* |
| 15 | 32193644 | rs6494040 | T | C | 0.29 | -0.26 | 2.23E-04 | 0.31 | -0.4 | 4.45E-03 | 0.30 | -0.29 | 4.34E-06 | -0.29 | 4.34E-06 | 0.36 | 0 | -0.28 | 5.12E-06 | -0.28 | 0.000005124 | 0.4 | 0 | *OTUD7A* |
| 17 | 33297065 | rs1351554 | T | C | 0.11 | -0.36 | 2.79E-04 | 0.14 | -0.53 | 4.48E-03 | 0.12 | -0.39 | 5.05E-06 | -0.39 | 5.05E-06 | 0.41 | 0 | -0.4 | 4.63E-06 | -0.4 | 0.000004625 | 0.41 | 0 | *CCT6B* |
| 18 | 69708238 | rs150547673 | G | A | 0.043 | -0.55 | 4.14E-04 | 0.05 | -0.92 | 3.51E-03 | 0.045 | -0.63 | 7.38E-06 | -0.64 | 3.71E-05 | 0.29 | 9.59 | -0.62 | 9.51E-06 | -0.62 | 0.00001341 | 0.31 | 1.91 | *CBLN2* |
| 20 | 22678410 | rs2209794 | C | T | 0.16 | 0.29 | 6.47E-04 | 0.16 | 0.79 | 1.22E-05 | 0.16 | 0.39 | 5.77E-07 | 0.52 | 3.80E-02 | 0.011 | 84.4 | 0.38 | 6.51E-07 | 0.51 | 0.03719 | 0.012 | 84.05 | *LINC01747* |

[a] Results were obtained using fixed-effct model; [b] results were obtained using random-effect model.

EA, effect allele; NEA, non-effect allele; EAF, effect allele frequency. $I^2$ (%), Heterogeneity.

**Table S2. Results of the meta-GWAS of HKCES-1 and LAMP in the discovery stage**

| SNP | EA | EAF in HKCES 1 | EAF in HKCES 2 | EAF in LAMP | Original_P[a] | Bonferroni_P[a] | Original_P[b] | Bonferroni_P[b] |
|---|---|---|---|---|---|---|---|---|
| rs292034 | T | 0.013 | 0.011 | 0.011 | 0.54 | 1 | 1 | 1 |
| rs17074027 | T | 0.058 | 0.076 | 0.06 | 0.02 | 0.06 | 1 | 1 |
| rs6925312 | A | 0.78 | 0.78 | 0.76 | 0.24 | 0.97 | 1 | 1 |
| rs4609227 | G | 0.025 | 0.025 | 0.025 | 0.99 | 1 | 1 | 1 |

The chi-square test is used to test the effect allele frequency between two cohorts; bonferroni test was used for multiple comparison.

[a]Comparisons between HKCES 1 and HKCES 2; [b]comparisons between HKCES 1 and LAMP.

EA, effect allele; EAF, effect allele frequency.

**Table S3. Associations between axial length and 4 SNPs identified in this study**

| CHR | SNP | BP[a] | Nearby Loci | EA | NEA | Satge 1 | | | | | | | | Stage 2 | | | Stage 3 | |
|---|---|---|---|---|---|---|---|---|---|---|---|---|---|---|---|---|---|---|
| | | | | | | HKCES-1 (N=864) | | | LAMP (N=373) | | | Meta-GWAS (N=1237) | | HKCES-2 (N=2066) | | | All cohorts (N=3303) | |
| | | | | | | EAF | β | P | EAF | β | P | β[b] | P[b] | EAF | β | P | β[c] | P[c] |
| 4 | rs292034 | 28478119 | *MIR4275* | G | T | 0.013 | 0.68 | $3.31\times10^{-4}$ | 0.011 | 0.32 | 0.28 | 0.58 | $2.88\times10^{-4}$ | 0.011 | 0.35 | $4.76\times10^{-3}$ | 0.43 | $8.37\times10^{-6}$ |
| 4 | rs17074027 | 183678700 | *TENM3* | G | T | 0.058 | 0.28 | $1.32\times10^{-3}$ | 0.06 | 0.27 | 0.05 | 0.14 | $1.24\times10^{-3}$ | 0.076 | 0.13 | $4.52\times10^{-3}$ | 0.18 | $9.59\times10^{-6}$ |
| 6 | rs6925312 | 88612769 | *LOC101928911* | G | A | 0.78 | 0.16 | $5.32\times10^{-4}$ | 0.76 | 0.1 | 0.23 | 0.15 | $3.09\times10^{-3}$ | 0.78 | 0.09 | $1.07\times10^{-3}$ | 0.11 | $1.89\times10^{-6}$ |
| 8 | rs4609227 | 138229001 | *FAM135B* | C | G | 0.025 | 0.55 | $8.49\times10^{-6}$ | 0.025 | 0.076 | 0.71 | 0.31 | $5.15\times10^{-6}$ | 0.025 | 0.29 | $1.49\times10^{-4}$ | 0.34 | $5.03\times10^{-8}$ |

CHR, chromosome; SNP, single nucleotide polymorphism; BP, base pair position; EA, effect allele; EAF, effect allele frequency; NEA, non-effect allele.

[a] Position is based on NCBI human genome build 37.

[b] Meta-analysis of AL used HKCES-1 and LAMP.

[c] Meta-analysis of AL used HKCES-1, LAMP and HKCES-2.

**Table S4. Association between rs292034 and different myopia severities**

| Cohort | EAF | | | | | | EMM vs. Hyperopia | | Myopia vs. NM | | Mild myopia vs. NM | | MH myopia vs. NM | |
|---|---|---|---|---|---|---|---|---|---|---|---|---|---|---|
| | Hyperopia | EMM | Myo | NM | Mild myopia | MH myopia | OR (95%CI) | P | OR (95%CI) | P | OR (95%CI) | P | OR (95%CI) | P |
| HKCES-1 | 0.0067 | 0.012 | 0.032 | 0.0087 | 0.021 | 0.093 | 1.72 (0.55, 5.42) | 0.35 | 3.42 (1.42, 8.22) | $\mathbf{6.01\times10^{-3}}$ | 2.39 (0.85, 6.68) | 0.10 | 9.33 (2.77, 31.46) | $\mathbf{3.16\times10^{-4}}$ |
| HKCES-2 | 0.0052 | 0.016 | 0.014 | 0.0088 | 0.012 | 0.032 | 3.34 (1.59, 7.02) | $\mathbf{1.50\times10^{-3}}$ | 1.72 (0.83, 3.57) | 0.15 | 1.52 (0.69, 3.36) | 0.30 | 4.41 (0.98, 19.88) | 0.05 |
| Shantou | 0.031 | 0.0067 | 0.013 | 0.0084 | 0.011 | 0.019 | 0.22 (0.020, 2.38) | 0.21 | 1.36 (0.48, 3.85) | 0.57 | 1.20 (0.41, 3.51) | 0.74 | 1.97 (0.64, 6.09) | 0.24 |
| Meta-all | | | | | | | 2.33 (1.28, 4.27) | $\mathbf{5.94\times10^{-3}}$ | 2.03 (1.24, 3.32) | $\mathbf{5.06\times10^{-3}}$ | 1.62 (0.94, 2.79) | 0.081 | 4.13 (2.0, 8.51) | $\mathbf{1.30\times10^{-4}}$ |

EAF, effect allele frequency; Hyperopia (SE ≥ 0.5D); EMM, emmetropia (-0.5D <SE <0.5D); MH myopia, moderate and high myopia (SE ≤ -3.0D); NM: no myopia (Hyperopia + EMM). The sample sizes for Hyperopia, EMM, Myo, NM, Mild myopia and MH myopia in HKCES-1 are 446, 246, 172, 692, 145 and 27; in HKCES-2 are 1140, 565, 361, 1705, 326 and 35; in Shantou are 17, 224, 1786, 241, 1257 and 529.

**Table S5. Association between rs17074027 and different myopia severities**

| Cohort | EAF | | | | | | EMM vs. Hyperopia | | Myopia vs. NM | | Mild myopia vs. NM | | MH myopia vs. NM | |
|---|---|---|---|---|---|---|---|---|---|---|---|---|---|---|
| | Hyperopia | EMM | Myo | NM | Mild myopia | MH myopia | OR (95%CI) | P | OR (95%CI) | P | OR (95%CI) | P | OR (95%CI) | P |
| HKCES-1 | 0.047 | 0.045 | 0.1 | 0.046 | 0.093 | 0.17 | 0.91 (0.53, 1.57) | 0.73 | 2.37 (1.49, 3.77) | **$2.81\times10^{-4}$** | 2.10 (1.26, 3.47) | **$4.16\times10^{-3}$** | 4.23 (1.77, 10.10) | **$1.15\times10^{-3}$** |
| HKCES-2 | 0.063 | 0.081 | 0.088 | 0.069 | 0.074 | 0.22 | 1.29 (0.97, 1.71) | 0.077 | 1.27 (0.94, 1.71) | 0.12 | 1.05 (0.75, 1.46) | 0.77 | 3.88 (2.01, 7.50) | **$5.47\times10^{-5}$** |
| Shantou | 0.029 | 0.051 | 0.076 | 0.05 | 0.075 | 0.079 | 1.84 (0.23, 14.69) | 0.56 | 1.66 (1.07, 2.57) | 0.025 | 1.59 (1.01, 2.51) | 0.043 | 1.82 (1.12, 3.00) | 0.016 |
| Meta-all | | | | | | | 1.20 (0.94, 1.55) | 0.14 | 1.55 (1.25, 1.94) | **$7.99\times10^{-5}$** | 1.37 (1.08, 1.74) | **$9.55\times10^{-3}$** | 2.62 (1.84, 3.77) | **$1.24\times10^{-7}$** |

EAF, effect allele frequency; Hyperopia (SE ≥ +0.5D); EMM, emmetropia (-0.5D <SE <0.5D); MH myopia, moderate and high myopia (SE ≤ -3.0D); NM: no myopia (Hyperopia + EMM). The sample sizes for Hyperopia, EMM, Myo, NM, Mild myopia and MH myopia in HKCES-1 are 446, 246, 172, 692, 145 and 27; in HKCES-2 are 1140, 565, 361, 1705, 326 and 35; in Shantou are 17, 224, 1786, 241, 1257 and 529.

**Table S6. Association between rs6925312 and different myopia severities**

| Cohort | EAF | | | | | | EMM vs. Hyperopia | | Myopia vs. NM | | Mild myopia vs. NM | | MH myopia vs. NM | |
|---|---|---|---|---|---|---|---|---|---|---|---|---|---|---|
| | Hyperopia | EMM | Myo | NM | Mild myopia | MH myopia | OR (95%CI) | P | OR (95%CI) | P | OR (95%CI) | P | OR (95%CI) | P |
| HKCES-1 | 0.74 | 0.77 | 0.83 | 0.75 | 0.81 | 0.93 | 1.23 (0.95, 1.62) | 0.12 | 1.57 (1.14, 2.16) | **$5.66{\times}10^{-3}$** | 1.39 (1, 1.93) | 0.053 | 4.64 (1.61, 13.33) | **$4.37{\times}10^{-3}$** |
| HKCES-2 | 0.23 | 0.22 | 0.17 | 0.23 | 0.17 | 0.13 | 1.05 (0.88, 1.25) | 0.56 | 1.44 (1.16, 1.78) | **$7.80{\times}10^{-4}$** | 1.40 (1.13, 1.75) | **$2.60{\times}10^{-3}$** | 1.88 (0.94, 3.77) | 0.076 |
| Shantou | 0.82 | 0.75 | 0.79 | 0.76 | 0.79 | 0.81 | 0.74 (0.30, 1.84) | 0.52 | 1.20 (0.96, 1.51) | 0.11 | 1.17(0.93, 1.48) | 0.18 | 1.32 (1.01, 1.73) | 0.043 |
| Meta-all | | | | | | | 1.09 (0.94, 1.26) | 0.22 | 1.37 (1.19, 1.57) | **$1.13{\times}10^{-5}$** | 1.31 (1.13, 1.51) | **$2.63{\times}10^{-4}$** | 1.48 (1.16, 1.88) | **$1.80{\times}10^{-3}$** |

EAF, effect allele frequency; Hyperopia (SE ≥ +0.5D); EMM, emmetropia (-0.5D <SE <0.5D); MH myopia, moderate and high myopia (SE ≤ -3.0D); NM: no myopia (Hyperopia + EMM). The sample sizes for Hyperopia, EMM, Myo, NM, Mild myopia and MH myopia in HKCES-1 are 446, 246, 172, 692, 145 and 27; in HKCES-2 are 1140, 565, 361, 1705, 326 and 35; in Shantou are 17, 224, 1786, 241, 1257 and 529.

**Table S7. Association between rs4609227 and different myopia severities**

| Cohort | EAF | | | | | | EMM vs. Hyperopia | | Myopia vs. NM | | Mild myopia vs. NM | | MH myopia vs. NM | |
|---|---|---|---|---|---|---|---|---|---|---|---|---|---|---|
| | Hyperopia | EMM | Myo | NM | Mild myopia | MH myopia | OR (95%CI) | P | OR (95%CI) | P | OR (95%CI) | P | OR (95%CI) | P |
| HKCES-1 | 0.015 | 0.033 | 0.044 | 0.021 | 0.021 | 0.18 | 2.23 (1.05, 4.72) | 0.037 | 1.74 (0.89, 3.39) | 0.10 | 0.89 (0.36, 2.23) | 0.80 | 8.85 (3.35, 23.35) | **$1.08 \times 10^{-5}$** |
| HKCES-2 | 0.018 | 0.023 | 0.05 | 0.02 | 0.046 | 0.11 | 1.24 (0.75, 2.06) | 0.4 | 2.60 (1.68, 4.01) | **$1.62 \times 10^{-5}$** | 2.37 (1.50, 3.73) | **$2.16 \times 10^{-4}$** | 7.53 (2.64, 21.49) | **$1.59 \times 10^{-4}$** |
| Shantou | 0.029 | 0.036 | 0.037 | 0.035 | 0.037 | 0.038 | 1.31 (0.16, 10.6) | 0.8 | 1.05 (0.62, 1.79) | 0.85 | 1.06 (0.62, 1.81) | 0.84 | 1.06 (0.57, 1.97) | 0.85 |
| Meta-all | | | | | | | 1.48 (0.98, 2.23) | 0.061 | 1.79 (1.33, 2.42) | **$1.34 \times 10^{-4}$** | 1.56 (1.13, 2.16) | **$7.46 \times 10^{-3}$** | 2.55 (1.61, 4.09) | **$8.25 \times 10^{-5}$** |

EAF, effect allele frequency; Hyperopia (SE ≥ +0.5D); EMM, emmetropia (-0.5D <SE <0.5D); MH myopia, moderate and high myopia (SE ≤ -3.0D); NM: no myopia (Hyperopia + EMM). The sample sizes for Hyperopia, EMM, Myo, NM, Mild myopia and MH myopia in HKCES-1 are 446, 246, 172, 692, 145 and 27; in HKCES-2 are 1140, 565, 361, 1705, 326 and 35; in Shantou are 17, 224, 1786, 241, 1257 and 529.

**Table S8. Associations between spherical equivalent and 4 SNPs in adults**

| | | | | | Adult-1 | | | | | | Adult-2 | | | | | | Meta adult | | | |
|---|---|---|---|---|---|---|---|---|---|---|---|---|---|---|---|---|---|---|---|---|
| CHR | SNP | BP | EA | NEA | EAF | BETA | SE | L95 | U95 | P | EAF | BETA | SE | L95 | U95 | P | BETA | P | Q test | $I^2$ |
| 4 | rs292034 | 28478119 | G | T | 0.017 | 0.67 | 0.45 | -0.2 | 1.55 | 0.13 | 0.017 | -0.44 | 0.79 | -1.99 | 1.10 | 0.57 | 0.40 | 0.30 | 0.22 | 34.11 |
| 4 | rs17074027 | 183678700 | G | T | 0.077 | -0.44 | 0.21 | -0.86 | -0.02 | 0.039 | 0.068 | -0.74 | 0.42 | -1.57 | 0.09 | 0.08 | -0.50 | 0.0083 | 0.53 | 0 |
| 6 | rs6925312 | 88612769 | G | A | 0.79 | -0.10 | 0.13 | -0.37 | 0.16 | 0.44 | 0.77 | -0.2 | 0.24 | -0.27 | 0.66 | 0.40 | -0.031 | 0.79 | 0.27 | 19.25 |
| 8 | rs4609227 | 138229001 | C | G | 0.028 | -0.71 | 0.35 | -1.39 | -0.02 | 0.04 | 0.026 | -1.46 | 0.66 | -2.76 | -0.16 | 0.028 | -0.87 | 0.0051 | 0.32 | 0.45 |

EA, effect allele; NEA, non-effect allele; EAF, effect allele frequency. $I^2$, Heterogeneity.

**Table S9. SNP×Age interaction analysis of 4 significant SNPs from GWAS of HKCES-1 cohort**

| | rs292034 | | | rs17074027 | | | rs6925312 | | | rs4609227 | | |
|---|---|---|---|---|---|---|---|---|---|---|---|---|
| | **β** | **Std.error** | **P** | **β** | **Std.error** | **P** | **β** | **Std.error** | **P** | **β** | **Std.error** | **P** |
| Intercept | 2.3 | 0.42 | 7.49E-08 | 2.41 | 0.44 | 6.26E-08 | 1.53 | 1.08 | 0.16 | 2.3 | 0.43 | 8.56E-08 |
| Age | -0.32 | 0.05 | 7.55E-10 | -0.33 | 0.05 | 1.94E-09 | -0.16 | 0.14 | 0.27 | -0.32 | 0.05 | 1.72E-09 |
| SNP | 4.07 | 2.51 | 0.1 | 0.04 | 1.27 | 0.97 | 0.62 | 0.65 | 0.34 | 1.97 | 1.64 | 0.23 |
| SNP×Age | -0.66 | 0.31 | **0.036** | -0.09 | 0.16 | 0.59 | -0.13 | 0.09 | 0.14 | -0.37 | 0.2 | 0.07 |
| Sex | 0.33 | 0.1 | 0.00058 | 0.32 | 0.1 | 0.00086 | 0.32 | 0.1 | 0.0009 | 0.32 | 0.1 | 0.001 |
| PC1 | 0.66 | 3.14 | 0.83 | -0.3 | 3.14 | 0.92 | 0.33 | 3.14 | 0.92 | 0.29 | 3.13 | 0.93 |
| PC2 | -3.44 | 5.14 | 0.5 | -1.79 | 5.15 | 0.73 | -3.07 | 5.15 | 0.55 | -3.11 | 5.12 | 0.54 |
| PC3 | 0.51 | 2.64 | 0.85 | 0.02 | 2.64 | 0.99 | -0.05 | 2.64 | 0.98 | 0.33 | 2.63 | 0.9 |
| PC4 | 1.75 | 1.49 | 0.24 | 2.22 | 1.5 | 0.14 | 1.78 | 1.49 | 0.23 | 1.67 | 1.48 | 0.26 |
| PC5 | -1.48 | 2.22 | 0.5 | -2.09 | 2.22 | 0.35 | -1.87 | 2.22 | 0.4 | -1.51 | 2.21 | 0.5 |

SNP, single-nucleotide polymorphism; GWAS, genome-wide association study; HKCES, Hong Kong Children Eye Study; Std.error, standard error. PC, principal component.

**Table S10. SNP×Age interaction analysis of 4 significant SNPs in GWAS of LAMP cohort**

| | rs292034 | | | rs17074027 | | | rs6925312 | | | rs4609227 | | |
|---|---|---|---|---|---|---|---|---|---|---|---|---|
| | β | Std.error | P | β | Std.error | P | β | Std.error | P | β | Std.error | P |
| (Intercept) | -2 | 0.52 | 0.00015 | -1.74 | 0.54 | 0.0014 | -1.18 | 1.48 | 0.43 | -1.89 | 0.53 | 0.0004 |
| Age | -0.2 | 0.06 | 0.00029 | -0.22 | 0.06 | 0.00015 | -0.24 | 0.17 | 0.16 | -0.22 | 0.06 | 0.00017 |
| SNP | -3.73 | 3.25 | 0.25 | -1.65 | 1.53 | 0.28 | -0.48 | 0.86 | 0.58 | -1.85 | 1.76 | 0.29 |
| SNP×Age | 0.27 | 0.35 | 0.45 | 0.14 | 0.19 | 0.44 | 0.02 | 0.1 | 0.87 | 0.11 | 0.21 | 0.6 |
| Sex | -0.15 | 0.19 | 0.44 | -0.2 | 0.19 | 0.31 | -0.17 | 0.19 | 0.37 | -0.13 | 0.19 | 0.49 |
| PC1 | -0.65 | 1.79 | 0.72 | -0.63 | 1.8 | 0.73 | -0.59 | 1.8 | 0.74 | -0.57 | 1.79 | 0.75 |
| PC2 | -3.52 | 1.78 | 0.05 | -3.61 | 1.78 | 0.04 | -3.97 | 1.79 | 0.03 | -3.62 | 1.78 | 0.04 |
| PC3 | -0.86 | 1.79 | 0.63 | -0.84 | 1.79 | 0.64 | -0.83 | 1.79 | 0.64 | -0.9 | 1.79 | 0.61 |
| PC4 | -1.39 | 1.78 | 0.44 | -1.49 | 1.79 | 0.4 | -1.21 | 1.82 | 0.51 | -1.44 | 1.78 | 0.42 |
| PC5 | -5.08 | 1.79 | 0.0048 | -4.99 | 1.79 | 0.01 | -4.8 | 1.8 | 0.01 | -4.97 | 1.79 | 0.01 |
| PC6 | 0.09 | 1.82 | 0.96 | 0.28 | 1.82 | 0.88 | 0.23 | 1.82 | 0.9 | 0.28 | 1.82 | 0.88 |
| PC7 | -0.06 | 1.79 | 0.97 | -0.22 | 1.79 | 0.9 | 0 | 1.8 | 1 | -0.09 | 1.78 | 0.96 |

SNP, single-nucleotide polymorphism; GWAS, genome-wide association study; HKCES, Hong Kong Children Eye Study; Std.error, standard error. PC, principal component.

**Table S11. Comparison of SNPs previously associated with myopia in European adults and their effects in our children cohorts**

| | | | | | | Meta-GWAS of European adult cohorts[a] | | | | Meta-GWAS of our Chinese children corhorts | | | | |
|---|---|---|---|---|---|---|---|---|---|---|---|---|---|---|
| CHR | SNP | BP | Nearest genes | EA | NEA | EAF of European adults | P | Direction | Heterogeneity $I^2$ (%) | EAF of HKCES | EAF of LAMP | P | Direction | Heterogeneity $I^2$ (%) |
| 1 | rs6668720 | 2420913 | *PLCH2* | A | G | 0.37 | 2.81E-09 | +?+++ | 13.9 | 0.19 | 0.21 | 4.27E-01 | +- | 0 |
| 1 | rs902356 | 8432653 | *RERE* | A | G | 0.24 | 1.74E-08 | +++++ | 35.7 | 0.77 | 0.74 | 1.77E-01 | -+ | 86.31 |
| 1 | rs11121599 | 10666735 | *PEX14* | A | G | 0.21 | 1.76E-09 | +++++ | 59.8 | NA | NA | NA | NA | NA |
| 1 | rs3013105 | 13802325 | *LRRC38* | T | C | 0.6 | 2.49E-09 | ----- | 44.1 | 0.36 | 0.37 | 3.61E-01 | ++ | 0 |
| 1 | rs10753502 | 20759231 | *VWA5B1* | A | C | 0.33 | 1.2E-09 | +++++ | 2.8 | 0.46 | 0.48 | 5.26E-01 | -+ | 0 |
| 1 | rs10158878 | 23259421 | *EPHB2* | C | G | 0.74 | 5.16E-10 | +++++ | 0 | NA | NA | NA | NA | NA |
| 1 | rs3131703 | 40419681 | *TRIT1* | A | G | 0.41 | 4.09E-10 | ----- | 2.9 | NA | NA | NA | NA | NA |
| 1 | rs6670896 | 42334793 | *HIVEP3* | A | G | 0.68 | 5.21E-36 | ----- | 75.3 | 0.42 | 0.43 | 1.81E-01 | -- | 0 |
| 1 | rs673253 | 44062154 | *SZT2* | T | C | 0.44 | 2.72E-13 | ----- | 0 | 0.2 | 0.18 | 3.63E-01 | ++ | 0 |
| 1 | rs12409365 | 48107910 | *TRABD2B* | C | G | 0.94 | 3.16E-10 | ----- | 45.7 | NA | NA | NA | NA | NA |
| 1 | rs72660683 | 54191811 | *GLIS1* | T | C | 0.78 | 7.2E-13 | +++++ | 0 | NA | NA | NA | NA | NA |
| 1 | rs556161 | 61324813 | *NFIA* | A | G | 0.36 | 1.96E-28 | ----- | 69.5 | 0.52 | 0.52 | 1.11E-01 | -- | 0 |
| 1 | rs10493817 | 89126887 | *PKN2* | T | G | 0.62 | 3.35E-08 | ----- | 0 | 0.65 | 0.66 | 2.22E-01 | ++ | 0 |
| 1 | rs12568187 | 91193691 | *BARHL2* | T | C | 0.56 | 1.24E-30 | +++++ | 27.1 | 0.6 | 0.6 | 5.58E-02 | ++ | 0 |
| 1 | rs11165052 | 94444060 | *FAM69A* | A | C | 0.32 | 3.2E-10 | +++++ | 39.7 | 0.17 | 0.16 | 5.13E-01 | ++ | 0 |
| 1 | rs501057 | 108087825 | *PRMT6* | A | G | 0.5 | 7.17E-15 | ----- | 56.3 | 0.59 | 0.57 | 7.87E-01 | +- | 0 |
| 1 | rs4839270 | 113477320 | *FAM19A3* | A | T | 0.23 | 7.8E-20 | ----- | 58.3 | 0.03 | 0.03 | 4.37E-02 | -- | 74.09 |
| 1 | rs942453 | 150257262 | *OTUD7B* | A | G | 0.87 | 2.93E-15 | ----- | 0 | NA | NA | NA | NA | NA |
| 1 | rs4845402 | 154977929 | *FLAD1* | A | G | 0.14 | 5.36E-13 | ----- | 0 | NA | NA | NA | NA | NA |
| 1 | rs17420551 | 158039425 | *RRNAD1* | A | T | 0.72 | 7.01E-38 | +++++ | 0 | 0.96 | 0.96 | 3.93E-01 | -- | 0 |
| 1 | rs1556867 | 164213686 | *PBX1* | T | C | 0.24 | 9.45E-48 | ----- | 74.9 | 0.67 | 0.71 | 2.78E-01 | -+ | 67.04 |
| 1 | rs10753823 | 170798444 | *PRRX1* | A | G | 0.68 | 2.78E-11 | ----- | 0 | 0.45 | 0.46 | 5.67E-01 | -+ | 0 |
| 1 | rs505837 | 179871712 | *TOR1AIP2* | A | G | 0.64 | 3.09E-09 | ----- | 41.4 | 0.74 | 0.75 | 6.13E-01 | +- | 68.8 |
| 1 | rs12569272 | 192085298 | *RGS18* | T | G | 0.82 | 3.06E-08 | +++++ | 8.7 | 0.38 | 0.38 | 6.66E-02 | ++ | 0 |
| 1 | rs2808514 | 200343081 | *PTPRC* | A | G | 0.61 | 1.18E-52 | +++++ | 26.9 | 0.91 | 0.88 | 8.89E-01 | -+ | 0 |
| 1 | rs7549293 | 205312280 | *NUAK2* | C | G | 0.41 | 7.79E-13 | +++++ | 0 | 0.38 | 0.34 | 8.43E-01 | +- | 63.55 |
| 1 | rs2017760 | 207455421 | *SRGAP2* | T | G | 0.31 | 2.17E-54 | ----- | 3.7 | 0.68 | 0.66 | 4.50E-01 | ++ | 0 |
| 1 | rs11119421 | 210321043 | *SYT14* | A | G | 0.69 | 1.49E-13 | +++++ | 32.7 | 0.58 | 0.57 | 6.84E-01 | +- | 65.88 |
| 1 | rs6700065 | 214135660 | *RPS6KC1* | T | G | 0.66 | 1.09E-10 | ----- | 56.6 | 0.79 | 0.81 | 9.29E-01 | -- | 0 |
| 1 | rs11585774 | 217222415 | *ESRRG* | A | G | 0.2 | 2.56E-08 | +++++ | 0 | NA | NA | NA | NA | NA |
| 1 | rs7525202 | 219788519 | *TGFB2* | A | G | 0.45 | 1.2E-40 | ----- | 15.6 | 0.72 | 0.73 | 2.38E-01 | -- | 0 |
| 1 | rs6667260 | 226923938 | *ITPKB* | A | C | 0.54 | 1.25E-12 | +++++ | 0 | 0.34 | 0.34 | 2.79E-01 | +- | 0 |
| 1 | rs1490276 | 238130842 | *ZP4* | T | C | 0.52 | 6.91E-10 | +++++ | 17.7 | 0.51 | 0.51 | 5.84E-01 | -+ | 0 |
| 2 | rs300751 | 209063 | *FAM110C* | A | C | 0.5 | 6.45E-25 | ----- | 80.2 | 0.7 | 0.71 | 4.33E-01 | -- | 0 |
| 2 | rs11675358 | 2919797 | *TSSC1* | A | T | 0.71 | 5.47E-10 | +++++ | 0 | 0.73 | 0.72 | 7.07E-01 | -- | 0 |
| 2 | rs28495254 | 16226048 | *MYCN* | T | C | 0.91 | 1.56E-09 | ----- | 41.3 | 0.96 | 0.97 | 1.14E-01 | -- | 0 |
| 2 | rs1260326 | 27730940 | *SNX17* | T | C | 0.4 | 4.73E-10 | ----+ | 37.4 | 0.46 | 0.47 | 7.31E-01 | -- | 0 |
| 2 | rs11127261 | 30475153 | *LBH* | A | G | 0.28 | 2.05E-20 | ----- | 79.6 | NA | NA | NA | NA | NA |

**Table S11. Comparison of SNPs previously associated with myopia in European adults and their effects in our children cohorts**

| | | | | | | Meta-GWAS of European adult cohorts[a] | | | | Meta-GWAS of our Chinese children corhorts | | | | |
|---|---|---|---|---|---|---|---|---|---|---|---|---|---|---|
| CHR | SNP | BP | Nearest genes | EA | NEA | EAF of European adults | P | Direction | Heterogeneity $I^2$ (%) | EAF of HKCES | EAF of LAMP | P | Direction | Heterogeneity $I^2$ (%) |
| 2 | rs13409142 | 32666137 | *YIPF4* | T | C | 0.66 | 2.47E-09 | ----- | 0 | NA | NA | NA | NA | NA |
| 2 | rs1558655 | 40586022 | *SLC8A1* | A | T | 0.43 | 5.99E-09 | +++++ | 5.2 | 0.66 | 0.63 | 5.53E-01 | -+ | 0 |
| 2 | rs17032696 | 45137870 | *ABCG8* | A | C | 0.81 | 2.6E-26 | ----- | 54.9 | 0.51 | 0.53 | 9.78E-01 | -+ | 65.84 |
| 2 | rs17817004 | 46735423 | *EPAS1* | T | C | 0.37 | 1.05E-11 | +++++ | 0.8 | 0.06 | 0.06 | 9.43E-01 | +- | 0 |
| 2 | rs12475184 | 48293584 | *FBXO11* | T | C | 0.71 | 5.67E-09 | +++++ | 23.8 | NA | NA | NA | NA | NA |
| 2 | rs9309272 | 56095366 | *ASB3* | T | G | 0.13 | 2.59E-34 | ----- | 0 | NA | NA | NA | NA | NA |
| 2 | rs359260 | 60486936 | *BCL11A* | T | G | 0.63 | 1.54E-11 | +++++ | 13.6 | 0.84 | 0.8 | 6.98E-01 | ++ | 0 |
| 2 | rs11555696 | 74588605 | *SLC4A5* | A | G | 0.03 | 2.01E-11 | +?+++ | 0 | NA | NA | NA | NA | NA |
| 2 | rs6547257 | 79918454 | *CTNNA2* | A | T | 0.67 | 3.37E-12 | ----- | 0 | 0.89 | 0.92 | 3.39E-01 | -- | 32.63 |
| 2 | rs2570495 | 104443942 | *NA* | T | C | 0.6 | 4.61E-15 | +++++ | 0 | 0.31 | 0.34 | 2.51E-01 | -+ | 11.19 |
| 2 | rs7584334 | 124950895 | *CNTNAP5* | T | C | 0.22 | 5.77E-10 | -+--- | 35.1 | NA | NA | NA | NA | NA |
| 2 | rs61049169 | 146888708 | *ZEB2* | A | G | 0.45 | 5.34E-69 | +++++ | 0 | NA | NA | NA | NA | NA |
| 2 | rs7601475 | 148410887 | *ACVR2A* | A | G | 0.41 | 2.38E-10 | +++++ | 39.2 | 0.25 | 0.25 | 2.41E-01 | ++ | 0 |
| 2 | rs57902545 | 153702724 | *ARL6IP6* | T | C | 0.61 | 6.17E-11 | +++++ | 0 | 0.95 | 0.95 | 8.93E-01 | +- | 0 |
| 2 | rs192716 | 157376472 | *GPD2* | C | G | 0.71 | 1.28E-35 | +++++ | 50.2 | 0.81 | 0.79 | 1.31E-01 | ++ | 0 |
| 2 | rs10174570 | 161305018 | *BAZ2B* | A | G | 0.24 | 7.72E-10 | +++++ | 0 | NA | NA | NA | NA | NA |
| 2 | rs935402 | 166185721 | *SCN2A* | A | C | 0.74 | 1.1E-11 | +++++ | 47 | 0.62 | 0.64 | 3.95E-01 | -- | 0 |
| 2 | rs17428076 | 172851936 | *DYNC1I2* | C | G | 0.76 | 1.85E-39 | ----- | 25.7 | 0.94 | 0.94 | 7.12E-01 | +- | 51.97 |
| 2 | rs2573081 | 178828507 | *HNRNPA3* | C | G | 0.53 | 1.12E-70 | +++++ | 51.2 | 0.57 | 0.53 | 7.52E-02 | ++ | 0 |
| 2 | rs7602799 | 182260252 | *ITGA4* | T | C | 0.39 | 3.32E-08 | +++++ | 0 | 0.78 | 0.76 | 5.36E-01 | -+ | 73.45 |
| 2 | rs73042687 | 184351234 | *NA* | T | G | 0.22 | 7.53E-11 | ++++- | 42.7 | 0.54 | 0.57 | 4.42E-01 | ++ | 54.22 |
| 2 | rs13027546 | 208359507 | *CREB1* | C | G | 0.18 | 2.01E-08 | +++++ | 30.3 | NA | NA | NA | NA | NA |
| 2 | rs121908120 | 219755011 | *ZNF142* | A | T | 0.03 | 1.65E-17 | ----+ | 62.4 | NA | NA | NA | NA | NA |
| 2 | rs7569375 | 228093172 | *COL4A3* | T | C | 0.62 | 1.14E-08 | +++++ | 39.8 | 0.97 | 0.96 | 9.15E-01 | +- | 0 |
| 2 | rs1550094 | 233385396 | *B3GNT7* | A | G | 0.7 | 1.48E-123 | +?+++ | 90.1 | NA | NA | NA | NA | NA |
| 2 | rs7596847 | 239321118 | *TRAF3IP1* | A | T | 0.16 | 5.71E-16 | ----- | 36.1 | 0.19 | 0.18 | 7.85E-01 | +- | 0 |
| 3 | rs795288 | 4204455 | *LRRN1* | T | C | 0.68 | 2.78E-16 | +++++ | 15.9 | 0.59 | 0.57 | 3.42E-01 | -+ | 82.22 |
| 3 | rs178597 | 8196941 | *NA* | T | C | 0.59 | 6.28E-48 | +++++ | 0 | 0.16 | 0.14 | 8.47E-01 | +- | 0 |
| 3 | rs1568072 | 11041606 | *SLC6A1* | A | G | 0.23 | 1.57E-13 | +++++ | 0 | 0.24 | 0.26 | 8.73E-01 | -+ | 0 |
| 3 | rs13073161 | 16007825 | *ANKRD28* | T | C | 0.48 | 1.01E-17 | ----- | 42.5 | NA | NA | NA | NA | NA |
| 3 | rs1700943 | 24231030 | *NR1D2* | C | G | 0.47 | 4.38E-34 | ----- | 18.6 | NA | 0.55 | NA | NA- | NA |
| 3 | rs7427073 | 28163157 | *CMC1* | A | C | 0.71 | 9.76E-09 | ----- | 59.9 | 0.88 | 0.85 | 5.64E-02 | ++ | 0 |
| 3 | rs4130686 | 29443963 | *RBMS3* | C | G | 0.65 | 2.54E-12 | ----- | 21.9 | 0.52 | 0.56 | 6.69E-01 | ++ | 0 |
| 3 | rs6810039 | 33194990 | *CRTAP* | A | C | 0.4 | 2.01E-15 | +++++ | 0 | NA | NA | NA | NA | NA |
| 3 | rs12492279 | 41289031 | *EIF1B* | T | C | 0.54 | 3.31E-27 | +++++ | 0 | 0.75 | 0.7 | 2.21E-01 | +- | 82.35 |
| 3 | rs7634084 | 49949834 | *LRRC2* | A | T | 0.49 | 3.85E-14 | ----- | 23.8 | 0.86 | 0.86 | 9.77E-01 | -+ | 0 |
| 3 | rs78486117 | 51388394 | *DOCK3* | C | G | 0.08 | 7.77E-09 | ----- | 26 | NA | NA | NA | NA | NA |
| 3 | rs14165 | 53847408 | *TKT* | A | G | 0.31 | 9.04E-24 | +++++ | 0 | NA | NA | NA | NA | NA |

**Table S11. Comparison of SNPs previously associated with myopia in European adults and their effects in our children cohorts**

| | | | | | | Meta-GWAS of European adult cohorts[a] | | | | Meta-GWAS of our Chinese children corhorts | | | | |
|---|---|---|---|---|---|---|---|---|---|---|---|---|---|---|
| CHR | SNP | BP | Nearest genes | EA | NEA | EAF of European adults | P | Direction | Heterogeneity $I^2$ (%) | EAF of HKCES | EAF of LAMP | P | Direction | Heterogeneity $I^2$ (%) |
| 3 | rs1907462 | 56534434 | *ERC2* | A | G | 0.24 | 2.27E-10 | +++++ | 44.6 | 0.46 | 0.46 | 3.52E-01 | -+ | 0 |
| 3 | rs7431936 | 58263847 | *ABHD6* | A | G | 0.92 | 4.93E-14 | +++++ | 0 | NA | NA | NA | NA | NA |
| 3 | rs1562523 | 60933802 | *FHIT* | T | G | 0.44 | 1.02E-08 | +++++ | 0 | NA | NA | NA | NA | NA |
| 3 | rs35667547 | 64547477 | *THOC7* | C | G | 0.13 | 4.6E-11 | -?--+ | 67.9 | NA | NA | NA | NA | NA |
| 3 | rs60743220 | 68945153 | *FAM19A4* | T | C | 0.84 | 6.73E-24 | +++++ | 74.3 | 0.51 | 0.51 | 3.25E-01 | +- | 31.57 |
| 3 | rs11928769 | 70985059 | *FOXP1* | A | C | 0.3 | 3.58E-08 | +++++ | 0 | NA | NA | NA | NA | NA |
| 3 | rs1045960 | 73117835 | *PPP4R2* | T | C | 0.4 | 3.32E-11 | ----- | 36.9 | 0.09 | 0.08 | 6.96E-01 | ++ | 0 |
| 3 | rs73117594 | 78593913 | *ROBO1* | T | G | 0.11 | 2.03E-09 | +++++ | 0 | NA | NA | NA | NA | NA |
| 3 | rs6549043 | 85632126 | *CADM2* | A | T | 0.37 | 3.59E-18 | +++++ | 5.5 | 0.14 | 0.12 | 6.45E-01 | ++ | 0 |
| 3 | rs9824877 | 98896242 | *CPOX* | A | G | 0.22 | 5.11E-25 | ----- | 59.5 | NA | NA | NA | NA | NA |
| 3 | rs7619476 | 107800880 | *CD47* | A | G | 0.55 | 3.64E-10 | ----- | 29.2 | NA | NA | NA | NA | NA |
| 3 | rs35867 | 118739836 | *IGSF11* | T | C | 0.39 | 5.78E-16 | +++++ | 0 | 0.43 | 0.48 | 6.90E-01 | -+ | 0 |
| 3 | rs2124500 | 123093530 | *ADCY5* | T | C | 0.26 | 4.8E-08 | +++++ | 8.9 | NA | NA | NA | NA | NA |
| 3 | rs113761591 | 127305355 | *KLF15* | T | C | 0.2 | 9E-21 | +++++ | 0 | NA | NA | NA | NA | NA |
| 3 | rs10934960 | 130514936 | *PIK3R4* | A | G | 0.51 | 5.17E-11 | +++++ | 0 | 0.49 | 0.48 | 9.36E-02 | +- | 24.67 |
| 3 | rs3762672 | 132218623 | *DNAJC13* | T | G | 0.47 | 5.02E-09 | +++++ | 6.5 | 0.88 | 0.87 | 5.32E-01 | -+ | 52.45 |
| 3 | rs6767786 | 141104180 | *ZBTB38* | A | G | 0.35 | 1.45E-57 | +++++ | 53.4 | NA | NA | NA | NA | NA |
| 3 | rs62273875 | 147707460 | *ZIC1* | A | G | 0.83 | 8.45E-12 | ----- | 13.6 | 0.6 | 0.6 | 5.08E-01 | -- | 0 |
| 3 | rs41267883 | 150174658 | *TSC22D2* | A | G | 0.91 | 1.43E-10 | +++++ | 64.6 | 0.92 | 0.93 | 5.06E-01 | ++ | 0 |
| 3 | rs199771582 | 171989276 | *FNDC3B* | T | G | 0.81 | 1.89E-14 | +++++ | 0 | 0.7 | 0.69 | 2.40E-01 | ++ | 0 |
| 3 | rs9832291 | 178701523 | *ZMAT3* | T | C | 0.34 | 1.51E-09 | ----- | 0 | 0.45 | 0.42 | 2.46E-01 | -+ | 81.8 |
| 3 | rs1863622 | 186396616 | *HRG* | T | C | 0.78 | 1.01E-08 | ++++- | 54.3 | NA | NA | NA | NA | NA |
| 4 | rs57829920 | 5783386 | *EVC* | A | T | 0.56 | 1.71E-09 | ----- | 0 | NA | NA | NA | NA | NA |
| 4 | rs3756171 | 8602225 | *CPZ* | T | C | 0.25 | 1.78E-08 | +?+++ | 0 | 0.68 | 0.71 | 5.04E-01 | +- | 0 |
| 4 | rs9968377 | 18185077 | *LCORL* | T | C | 0.62 | 3.92E-09 | ----- | 36.1 | 0.8 | 0.8 | 5.62E-01 | -- | 0 |
| 4 | rs11734373 | 23939920 | *PPARGC1A* | A | G | 0.35 | 2.82E-11 | ----- | 38.6 | NA | NA | NA | NA | NA |
| 4 | rs6828872 | 30765027 | *PCDH7* | C | G | 0.53 | 3.47E-08 | +-+++ | 33.5 | 0.36 | 0.38 | 6.64E-02 | ++ | 0 |
| 4 | rs7691051 | 31993241 | *NA* | A | T | 0.33 | 4.27E-11 | ----- | 5.2 | 0.31 | 0.29 | 5.29E-01 | -- | 25.61 |
| 4 | rs33998475 | 42200204 | *BEND4* | A | G | 0.1 | 3.51E-09 | +++++ | 6 | 0.28 | 0.29 | 2.80E-01 | +- | 13.22 |
| 4 | rs28526098 | 44933151 | *GNPDA2* | A | C | 0.88 | 5.08E-18 | ----- | 56.4 | 0.63 | 0.62 | 9.38E-01 | -+ | 50.86 |
| 4 | rs10030755 | 62844408 | *LPHN3* | A | C | 0.8 | 1.55E-10 | +++++ | 0 | 0.57 | 0.58 | 7.72E-01 | -- | 0 |
| 4 | rs28564890 | 72535505 | *SLC4A4,GC* | T | C | 0.9 | 8.38E-09 | +++++ | 0 | 0.7 | 0.72 | 7.76E-01 | +- | 63.2 |
| 4 | rs74764079 | 81952637 | *ANXA3* | A | T | 0.03 | 1.99E-65 | ----- | 33.9 | NA | NA | NA | NA | NA |
| 4 | rs3775228 | 87985166 | *SLC10A6* | T | C | 0.4 | 6.59E-12 | +++++ | 56.4 | 0.39 | 0.39 | 1.36E-01 | +- | 67.02 |
| 4 | rs59473955 | 89757082 | *FAM13A* | T | C | 0.76 | 6.17E-33 | ----- | 72.7 | NA | NA | NA | NA | NA |
| 4 | rs13107325 | 103188709 | *BANK1* | T | C | 0.07 | 4.03E-17 | +++++ | 0 | NA | NA | NA | NA | NA |
| 4 | rs9790778 | 112129355 | *NA* | T | C | 0.42 | 1.37E-08 | +++++ | 0 | 0.22 | 0.22 | 3.15E-01 | ++ | 0 |
| 4 | rs12511880 | 120901526 | *MYOZ2* | A | T | 0.39 | 5.13E-18 | +++++ | 78.4 | NA | NA | NA | NA | NA |

**Table S11. Comparison of SNPs previously associated with myopia in European adults and their effects in our children cohorts**

| | | | | | | Meta-GWAS of European adult cohorts[a] | | | | Meta-GWAS of our Chinese children corhorts | | | | |
|---|---|---|---|---|---|---|---|---|---|---|---|---|---|---|
| CHR | SNP | BP | Nearest genes | EA | NEA | EAF of European adults | P | Direction | Heterogeneity $I^2$ (%) | EAF of HKCES | EAF of LAMP | P | Direction | Heterogeneity $I^2$ (%) |
| 4 | rs34687569 | 138497581 | *PCDH18* | T | C | 0.75 | 4.54E-11 | +++++ | 32.7 | 0.47 | 0.44 | 1.56E-01 | -- | 0 |
| 4 | rs73858679 | 141918504 | *RNF150* | A | C | 0.06 | 1.75E-08 | +++++ | 0 | NA | NA | NA | NA | NA |
| 4 | rs6537315 | 145724712 | *HHIP* | A | G | 0.44 | 5.42E-09 | +++++ | 0 | NA | NA | NA | NA | NA |
| 4 | rs11940147 | 149974078 | *NA* | T | G | 0.25 | 6.55E-12 | +++++ | 0 | NA | NA | NA | NA | NA |
| 4 | rs2045629 | 166777051 | *TLL1* | A | G | 0.44 | 7.99E-11 | +++++ | 0 | 0.84 | 0.82 | 6.71E-01 | -+ | 0 |
| 4 | rs147792504 | 174336590 | *GALNT7* | A | T | 0.02 | 2.01E-15 | -?--- | 59.1 | NA | NA | NA | NA | NA |
| 4 | rs35446926 | 183035265 | *TENM3* | T | C | 0.72 | 2.48E-11 | ----- | 0 | 0.47 | 0.47 | 2.28E-01 | -- | 0 |
| 5 | rs4499830 | 11852047 | *CTNND2* | T | C | 0.67 | 3E-08 | ----- | 0 | NA | NA | NA | NA | NA |
| 5 | rs2910644 | 41265864 | *C6* | A | G | 0.14 | 2.13E-08 | ----- | 0 | 0.27 | 0.28 | 5.30E-01 | ++ | 0 |
| 5 | rs75150349 | 45491724 | *FGF10* | T | G | 0.13 | 1.45E-13 | ----- | 0 | NA | NA | NA | NA | NA |
| 5 | rs35567312 | 49626919 | *EMB* | T | C | 0.11 | 7.03E-10 | ----- | 0 | NA | NA | NA | NA | NA |
| 5 | rs12514897 | 52670974 | *FST* | T | G | 0.89 | 5.99E-12 | +++++ | 22.7 | NA | NA | NA | NA | NA |
| 5 | rs3936511 | 55860781 | *MAP3K1* | A | G | 0.81 | 6.34E-09 | +++++ | 0 | 0.85 | 0.85 | 6.64E-01 | -+ | 12.83 |
| 5 | rs10223052 | 60800336 | *ELOVL7* | A | G | 0.36 | 8.83E-09 | ----- | 0 | NA | NA | NA | NA | NA |
| 5 | rs1309551 | 64288656 | *CWC27* | T | G | 0.55 | 1.84E-16 | ----- | 60.9 | 0.62 | 0.67 | 7.41E-01 | -- | 0 |
| 5 | rs7730838 | 71708698 | *MAP1B* | T | G | 0.45 | 1.4E-12 | +++++ | 60 | 0.88 | 0.87 | 3.45E-01 | ++ | 28.14 |
| 5 | rs256438 | 79366249 | *MTX3* | T | G | 0.64 | 1.1E-12 | +++++ | 12.9 | 0.77 | 0.81 | 2.18E-01 | ++ | 38.61 |
| 5 | rs7737179 | 87795525 | *TMEM161B* | A | G | 0.23 | 3.25E-23 | ----- | 1.9 | NA | NA | NA | NA | NA |
| 5 | rs4242244 | 92546237 | *NA* | T | G | 0.58 | 5.68E-09 | -+--- | 4.9 | 0.37 | 0.35 | 9.82E-01 | -+ | 0 |
| 5 | rs146172211 | 102620108 | *PAM* | A | C | 0.03 | 2.42E-10 | ++++- | 59.5 | NA | NA | NA | NA | NA |
| 5 | rs13189957 | 111836991 | *EPB41L4A* | A | G | 0.77 | 3.65E-08 | ----- | 41.2 | NA | NA | NA | NA | NA |
| 5 | rs10066524 | 123950880 | *ZNF608* | A | G | 0.21 | 4.14E-08 | ----- | 13.7 | 0.41 | 0.44 | 5.93E-01 | +- | 0 |
| 5 | rs6860901 | 127871750 | *FBN2* | T | C | 0.31 | 8.63E-11 | +++++ | 44.5 | 0.17 | 0.15 | 1.45E-01 | ++ | 0 |
| 5 | rs7732667 | 132070333 | *IL4* | C | G | 0.86 | 2.55E-10 | +++++ | 14.8 | 0.2 | 0.19 | 9.29E-01 | +- | 32.18 |
| 5 | rs246073 | 140317371 | *CXXC5* | T | C | 0.64 | 1.77E-14 | +++++ | 0 | 0.41 | 0.42 | 9.03E-01 | +- | 0 |
| 5 | rs6864640 | 143540718 | *YIPF5* | T | C | 0.21 | 1.95E-13 | ----- | 0 | 0.18 | 0.19 | 1.88E-01 | -- | 0 |
| 5 | rs62385438 | 158497639 | *EBF1* | T | C | 0.68 | 8.63E-18 | ----- | 21.3 | 0.81 | 0.78 | 7.22E-01 | -+ | 0 |
| 5 | rs10042371 | 165945863 | *NA* | T | G | 0.8 | 5.12E-12 | +++++ | 0 | 0.81 | 0.8 | 6.15E-01 | -- | 0 |
| 5 | rs6875105 | 173054917 | *BOD1* | T | C | 0.62 | 7.52E-09 | ----- | 40.1 | NA | 0.79 | NA | NA- | NA |
| 5 | rs13190379 | 174697891 | *DRD1* | A | T | 0.41 | 4.51E-16 | +++++ | 7.1 | NA | NA | NA | NA | NA |
| 5 | rs12186577 | 178359989 | *ZNF354B* | T | C | 0.9 | 8.63E-17 | +++++ | 47.4 | 0.88 | 0.87 | 6.17E-01 | -- | 0 |
| 6 | rs11758482 | 2438231 | *MAS1L* | A | G | 0.78 | 1.75E-20 | +++++ | 44.5 | 0.58 | 0.55 | 1.33E-01 | +- | 0 |
| 6 | rs9379066 | 6901856 | *LY86* | T | C | 0.64 | 1.01E-21 | +++++ | 24 | 0.85 | 0.83 | 3.54E-02 | ++ | 63.8 |
| 6 | rs115504329 | 11719478 | *ADTRP* | C | G | 0.06 | 3.23E-08 | +++++ | 37.7 | NA | NA | NA | NA | NA |
| 6 | rs4145443 | 22068174 | *CDKAL1* | T | G | 0.56 | 6.41E-38 | ----- | 82.1 | 0.13 | 0.15 | 8.41E-01 | +- | 27.55 |
| 6 | rs1150687 | 28162469 | *LRRC16A* | T | C | 0.61 | 4.32E-34 | +++++ | 0 | 0.42 | 0.42 | 1.84E-01 | ++ | 0 |
| 6 | rs4711751 | 43828582 | *GLTSCR1L* | T | C | 0.5 | 1.67E-12 | ----- | 0 | 0.75 | 0.77 | 7.57E-01 | -- | 0 |
| 6 | rs2076309 | 50789451 | *TFAP2D* | T | C | 0.46 | 4.19E-37 | +++++ | 26.7 | NA | NA | NA | NA | NA |

**Table S11. Comparison of SNPs previously associated with myopia in European adults and their effects in our children cohorts**

| | | | | | | Meta-GWAS of European adult cohorts[a] | | | | Meta-GWAS of our Chinese children corhorts | | | | |
|---|---|---|---|---|---|---|---|---|---|---|---|---|---|---|
| CHR | SNP | BP | Nearest genes | EA | NEA | EAF of European adults | P | Direction | Heterogeneity $I^2$ (%) | EAF of HKCES | EAF of LAMP | P | Direction | Heterogeneity $I^2$ (%) |
| 6 | rs7744813 | 73643289 | *OGFRL1* | A | C | 0.59 | 2.61E-205 | ----- | 91.5 | 0.78 | 0.82 | 5.51E-01 | -+ | 21.45 |
| 6 | rs171835 | 82464987 | *FAM46A* | A | G | 0.24 | 5.7E-14 | +++++ | 42.3 | 0.27 | 0.22 | 7.25E-01 | -+ | 0 |
| 6 | rs1118543 | 84380492 | *PRSS35* | C | G | 0.33 | 1.85E-26 | +++++ | 56.5 | 0.53 | 0.5 | 8.00E-01 | ++ | 0 |
| 6 | rs1906252 | 98550289 | *NA* | A | C | 0.49 | 2.44E-15 | ----+ | 74.7 | 0.43 | 0.38 | 4.11E-01 | -- | 0 |
| 6 | rs9484245 | 100193003 | *PRDM13* | A | C | 0.79 | 1.54E-15 | ----- | 0 | NA | NA | NA | NA | NA |
| 6 | rs11153164 | 109596309 | *CCDC162P* | A | G | 0.31 | 3.5E-08 | ----- | 0 | 0.39 | 0.38 | 8.00E-01 | +- | 78.72 |
| 6 | rs4947120 | 111819044 | *GTF3C6* | A | G | 0.86 | 3.07E-10 | ----- | 61.5 | NA | NA | NA | NA | NA |
| 6 | rs1064583 | 116446576 | *FRK* | A | G | 0.61 | 2.34E-36 | +++++ | 63.2 | NA | NA | NA | NA | NA |
| 6 | rs9372732 | 123348147 | *CLVS2* | T | C | 0.35 | 4.11E-11 | ----- | 0 | NA | NA | NA | NA | NA |
| 6 | rs12193446 | 129820038 | *LAMA2* | A | G | 0.91 | 0 | ----- | 93 | NA | NA | NA | NA | NA |
| 6 | rs4896068 | 134834538 | *SGK1* | A | G | 0.44 | 6.39E-10 | +++++ | 52.4 | 0.78 | 0.78 | 3.38E-01 | -- | 0 |
| 6 | rs7756435 | 141335140 | *NA* | C | G | 0.23 | 7.91E-12 | +++++ | 0 | 0.37 | 0.37 | 7.34E-01 | -- | 0 |
| 6 | rs9497026 | 144950023 | *UTRN* | T | G | 0.18 | 6.15E-10 | +++++ | 0 | 0.07 | 0.06 | 9.46E-01 | +- | 0 |
| 6 | rs4869965 | 151301241 | *MTHFD1L* | A | G | 0.3 | 5.14E-20 | ----- | 56.2 | 0.68 | 0.64 | 5.64E-01 | -+ | 0 |
| 6 | rs3127166 | 159248730 | *SYTL3* | A | G | 0.37 | 5.35E-09 | +++++ | 13.4 | NA | NA | NA | NA | NA |
| 6 | rs880095 | 164249854 | *QKI* | T | C | 0.56 | 4.54E-12 | +-+++ | 50.9 | 0.63 | 0.6 | 2.70E-01 | ++ | 0 |
| 7 | rs4721135 | 1912222 | *MAD1L1* | A | G | 0.59 | 1.79E-15 | -?--- | 7.6 | 0.51 | 0.51 | 5.92E-01 | +- | 0 |
| 7 | rs7786560 | 7252249 | *C1GALT1* | A | C | 0.39 | 1.56E-17 | +++++ | 0 | 0.06 | 0.06 | 2.07E-01 | -- | 0 |
| 7 | rs2355125 | 11920805 | *THSD7A* | A | G | 0.37 | 3.97E-08 | +++++ | 0 | 0.24 | 0.22 | 3.94E-01 | ++ | 0 |
| 7 | rs11772758 | 17079154 | *AGR3* | A | G | 0.22 | 8.61E-12 | ----- | 0 | 0.11 | 0.12 | 4.33E-01 | ++ | 0 |
| 7 | rs7780001 | 19477981 | *FERD3L* | A | T | 0.87 | 2.31E-09 | ----- | 0 | 0.81 | 0.82 | 5.67E-01 | -- | 0 |
| 7 | rs2075822 | 30490760 | *NOD1* | A | G | 0.77 | 1.41E-08 | +++++ | 0 | 0.78 | 0.78 | 5.53E-01 | -+ | 25.08 |
| 7 | rs2726052 | 36199170 | *EEPD1* | A | G | 0.22 | 2.74E-08 | +++++ | 0 | 0.03 | 0.03 | 8.39E-01 | +- | 0 |
| 7 | rs2696187 | 39043106 | *POU6F2* | T | C | 0.42 | 1.11E-11 | ----- | 0 | NA | NA | NA | NA | NA |
| 7 | rs7793034 | 42115516 | *GLI3* | A | G | 0.49 | 1.27E-10 | +++++ | 3 | 0.45 | 0.42 | 4.59E-01 | ++ | 27.47 |
| 7 | rs10258656 | 47763485 | *PKD1L1* | A | C | 0.22 | 1.17E-11 | +++++ | 61.6 | 0.29 | 0.31 | 6.69E-01 | -+ | 0 |
| 7 | rs12719025 | 51100190 | *COBL* | A | G | 0.54 | 3.11E-11 | +++++ | 52.8 | 0.81 | 0.79 | 6.92E-01 | -+ | 0 |
| 7 | rs7802500 | 69907025 | *AUTS2* | A | G | 0.21 | 1.97E-09 | +++++ | 31.3 | 0.22 | 0.23 | 7.79E-01 | +- | 0 |
| 7 | rs2867673 | 71752652 | *CALN1* | T | C | 0.48 | 2E-09 | ----- | 0 | 0.45 | 0.43 | 6.26E-01 | ++ | 0 |
| 7 | rs10260177 | 78075634 | *MAGI2* | T | C | 0.11 | 2.69E-17 | ----- | 0 | NA | NA | NA | NA | NA |
| 7 | rs6954078 | 82435878 | *PCLO* | T | G | 0.5 | 9.58E-24 | ----- | 2.8 | 0.83 | 0.83 | 8.10E-01 | -+ | 73.6 |
| 7 | rs144137329 | 84150814 | *SEMA3A* | T | C | 0.78 | 6.71E-11 | ----+ | 42.8 | 0.83 | 0.85 | 4.76E-01 | +- | 0 |
| 7 | rs7800245 | 86103733 | *GRM3* | C | G | 0.27 | 1.81E-16 | ----- | 48.8 | 0.26 | 0.3 | 2.15E-01 | -+ | 61.58 |
| 7 | rs854089 | 95303256 | *PDK4* | A | T | 0.3 | 8.04E-13 | +++++ | 46 | 0.32 | 0.29 | 1.37E-01 | +- | 0 |
| 7 | rs6946168 | 99598286 | *OR2AE1* | T | C | 0.29 | 3.38E-16 | ----- | 21.8 | 0.22 | 0.24 | 6.62E-01 | ++ | 0 |
| 7 | rs2708597 | 112056230 | *DOCK4* | C | G | 0.13 | 2.98E-11 | +++++ | 38.9 | 0.19 | 0.16 | 2.62E-01 | -+ | 0 |
| 7 | rs2024211 | 116153025 | *CAV2* | A | C | 0.73 | 5.9E-10 | +++++ | 52.4 | NA | NA | NA | NA | NA |
| 7 | rs148101639 | 124531504 | *GPR37* | A | G | 0.01 | 4.35E-08 | -?--+ | 18.7 | NA | NA | NA | NA | NA |

**Table S11. Comparison of SNPs previously associated with myopia in European adults and their effects in our children cohorts**

| | | | | | | Meta-GWAS of European adult cohorts[a] | | | | Meta-GWAS of our Chinese children corhorts | | | | |
|---|---|---|---|---|---|---|---|---|---|---|---|---|---|---|
| CHR | SNP | BP | Nearest genes | EA | NEA | EAF of European adults | P | Direction | Heterogeneity $I^2$ (%) | EAF of HKCES | EAF of LAMP | P | Direction | Heterogeneity $I^2$ (%) |
| 7 | rs17150996 | 127174679 | *GCC1* | A | G | 0.36 | 3.96E-08 | +++++ | 49.7 | 0.74 | 0.75 | 9.39E-01 | -+ | 0 |
| 7 | rs62468101 | 131550503 | *MKLN1* | T | C | 0.76 | 1.4E-11 | ----- | 0 | 0.97 | 0.96 | 2.13E-01 | -- | 0 |
| 7 | rs1646555 | 137604835 | *CREB3L2* | A | T | 0.37 | 2.09E-09 | ----+ | 48.1 | NA | NA | NA | NA | NA |
| 7 | rs58287690 | 151122699 | *WDR86* | T | G | 0.87 | 1.04E-20 | +?+++ | 0 | 0.95 | 0.94 | 7.42E-01 | ++ | 0 |
| 7 | rs12234576 | 158928375 | *PTPRN2* | A | G | 0.18 | 1.79E-37 | +++++ | 0 | 0.16 | 0.13 | 2.18E-02 | ++ | 0 |
| 8 | rs4875839 | 2490537 | *CSMD1* | C | G | 0.64 | 6.73E-10 | +++++ | 0 | 0.39 | 0.4 | 6.67E-02 | -- | 0 |
| 8 | rs10100265 | 10633159 | *SGK223* | A | C | 0.39 | 1E-24 | +++++ | 0 | 0.65 | 0.64 | 3.77E-01 | -+ | 86.54 |
| 8 | rs1905014 | 13407297 | *DLC1* | T | C | 0.57 | 1.41E-08 | ----- | 49.7 | 0.62 | 0.61 | 4.52E-01 | -- | 0 |
| 8 | rs11781149 | 22473158 | *DOK2* | T | C | 0.33 | 2.39E-17 | ----- | 0 | NA | NA | NA | NA | NA |
| 8 | rs1532276 | 27466157 | *CLU* | T | C | 0.4 | 3.35E-16 | ----- | 69.8 | 0.24 | 0.25 | 2.62E-01 | -- | 0 |
| 8 | rs16890057 | 40726582 | *ZMAT4* | A | G | 0.21 | 1.99E-81 | +++++ | 73.8 | 0.1 | 0.08 | 1.11E-01 | ++ | 0 |
| 8 | rs1371951 | 53385818 | *ST18* | C | G | 0.14 | 7.08E-25 | ----- | 49.6 | NA | NA | NA | NA | NA |
| 8 | rs61334940 | 57072570 | *LYN* | T | C | 0.81 | 1.14E-19 | ----- | 0 | 0.92 | 0.94 | 5.20E-01 | -+ | 0 |
| 8 | rs72621438 | 60178580 | *TOX* | C | G | 0.65 | 1E-153 | ----- | 65.9 | 0.55 | 0.59 | 4.07E-01 | -- | 0 |
| 8 | rs1425598 | 64753446 | *NA* | T | C | 0.71 | 3.5E-10 | +++++ | 0 | NA | NA | NA | NA | NA |
| 8 | rs4738094 | 71423744 | *NCOA2* | A | G | 0.65 | 1.21E-26 | ----- | 60.8 | NA | NA | NA | NA | NA |
| 8 | rs10112386 | 74281152 | *RDH10* | T | C | 0.83 | 8E-12 | +-+++ | 64 | NA | NA | NA | NA | NA |
| 8 | rs9650252 | 75731730 | *PI15* | T | C | 0.07 | 1.71E-13 | +++++ | 0 | 0.02 | 0.01 | 1.29E-02 | ++ | 0 |
| 8 | rs6995342 | 78951749 | *NA* | A | T | 0.63 | 3.57E-18 | ----- | 10.3 | 0.49 | 0.48 | 5.03E-01 | ++ | 0 |
| 8 | rs13268738 | 87673711 | *CNGB3* | T | C | 0.42 | 1.01E-14 | +++++ | 0 | NA | NA | NA | NA | NA |
| 8 | rs7816934 | 89312391 | *MMP16* | T | C | 0.9 | 1.15E-10 | +++++ | 36.4 | NA | NA | NA | NA | NA |
| 8 | rs2575725 | 97610840 | *SDC2* | A | G | 0.51 | 4.46E-09 | +++++ | 0 | NA | NA | NA | NA | NA |
| 8 | rs4133395 | 108314971 | *ANGPT1* | T | G | 0.61 | 2.72E-08 | +-+++ | 42.3 | 0.86 | 0.86 | 6.48E-01 | ++ | 0 |
| 8 | rs6988919 | 121630740 | *SNTB1* | C | G | 0.62 | 3.09E-27 | +++++ | 11.3 | 0.26 | 0.24 | 2.18E-01 | +- | 58.69 |
| 8 | rs1158569 | 131331012 | *ASAP1* | A | C | 0.49 | 3.48E-09 | ++++- | 41.4 | 0.39 | 0.38 | 9.29E-01 | -+ | 0 |
| 8 | rs1865249 | 142215982 | *DENND3* | A | C | 0.4 | 4.98E-10 | -?--- | 8.9 | 0.17 | 0.2 | 3.43E-01 | +- | 36.43 |
| 9 | rs7048915 | 4206388 | *GLIS3* | A | G | 0.26 | 3.94E-09 | ----- | 0 | NA | NA | NA | NA | NA |
| 9 | rs62538956 | 12679244 | *TYRP1* | T | C | 0.88 | 1.18E-11 | +++++ | 58.1 | 0.98 | 0.98 | 9.09E-01 | +- | 66.56 |
| 9 | rs1340044 | 18362105 | *ADAMTSL1* | A | T | 0.53 | 3.92E-51 | ----- | 27.9 | NA | NA | NA | NA | NA |
| 9 | rs7855188 | 20032153 | *SLC24A2* | A | T | 0.56 | 1.22E-13 | ----- | 29.8 | NA | NA | NA | NA | NA |
| 9 | rs1823289 | 22792513 | *NA* | C | G | 0.41 | 1.17E-09 | ----+ | 22 | NA | NA | NA | NA | NA |
| 9 | rs855719 | 33817617 | *PRSS3* | T | C | 0.42 | 4E-13 | ----- | 0 | 0.27 | 0.29 | 9.72E-01 | -+ | 0 |
| 9 | rs11145461 | 71766119 | *FXN* | T | C | 0.22 | 3.41E-39 | ----- | 84.1 | NA | 0.99 | NA | NA+ | NA |
| 9 | rs7042950 | 77149837 | *TMC1* | A | G | 0.78 | 6E-36 | +++++ | 75.4 | 0.2 | 0.19 | 1.10E-01 | -- | 0 |
| 9 | rs1961952 | 86325806 | *IDNK* | A | G | 0.61 | 5.7E-10 | ----- | 0 | NA | NA | NA | NA | NA |
| 9 | rs7849438 | 92038144 | *SEMA4D* | T | C | 0.67 | 2.62E-08 | ----- | 11.7 | 0.92 | 0.92 | 1.98E-01 | +- | 0 |
| 9 | rs13301794 | 100193001 | *TDRD7* | A | G | 0.52 | 4.79E-08 | +++++ | 3.5 | NA | NA | NA | NA | NA |
| 9 | rs1571590 | 101883808 | *TGFBR1* | A | G | 0.8 | 6.91E-25 | +++++ | 47.5 | NA | NA | NA | NA | NA |

**Table S11. Comparison of SNPs previously associated with myopia in European adults and their effects in our children cohorts**

| | | | | | | Meta-GWAS of European adult cohorts[a] | | | | Meta-GWAS of our Chinese children corhorts | | | | |
|---|---|---|---|---|---|---|---|---|---|---|---|---|---|---|
| CHR | SNP | BP | Nearest genes | EA | NEA | EAF of European adults | P | Direction | Heterogeneity $I^2$ (%) | EAF of HKCES | EAF of LAMP | P | Direction | Heterogeneity $I^2$ (%) |
| 9 | rs3764523 | 109765215 | *ZNF462* | A | G | 0.88 | 1.41E-15 | +++++ | 55.2 | NA | NA | NA | NA | NA |
| 9 | rs10980037 | 112544024 | *PALM2* | T | C | 0.76 | 4.44E-09 | ----- | 4.4 | 0.7 | 0.71 | 7.49E-01 | +- | 0 |
| 9 | rs41305473 | 116346118 | *RGS3* | A | G | 0.1 | 4.17E-08 | +++++ | 0 | NA | NA | NA | NA | NA |
| 9 | rs2072438 | 123651301 | *PHF19* | T | C | 0.44 | 1.65E-11 | ----- | 0 | 0.5 | 0.52 | 5.95E-01 | ++ | 0 |
| 9 | rs10122788 | 129206832 | *SCAI* | A | G | 0.57 | 1.1E-13 | +++++ | 0 | 0.73 | 0.72 | 6.62E-01 | -+ | 81.54 |
| 9 | rs943423 | 137437183 | *RXRA* | A | G | 0.7 | 4.56E-12 | +?+++ | 0 | NA | NA | NA | NA | NA |
| 9 | rs11103381 | 139097773 | *LHX3* | A | C | 0.68 | 1.37E-14 | -?--- | 81.2 | NA | NA | NA | NA | NA |
| 10 | rs12762379 | 21559884 | *NEBL* | T | C | 0.08 | 4.59E-09 | ----- | 10.6 | NA | NA | NA | NA | NA |
| 10 | rs10829282 | 27873025 | *RAB18* | A | T | 0.49 | 1.37E-08 | +++++ | 55.9 | 0.23 | 0.22 | 7.84E-01 | -- | 0 |
| 10 | rs3739996 | 30316872 | *NA* | T | C | 0.61 | 3.79E-19 | +++++ | 12.8 | 0.8 | 0.8 | 2.10E-01 | ++ | 1.97 |
| 10 | rs10793568 | 45415209 | *TMEM72* | A | G | 0.29 | 1.09E-14 | ----- | 0 | NA | 0.53 | NA | NA- | NA |
| 10 | rs56036047 | 49405013 | *ANXA8* | T | C | 0.73 | 1.98E-49 | +++++ | 0 | 0.93 | 0.93 | 3.63E-01 | -- | 0 |
| 10 | rs2842083 | 60329230 | *TFAM* | A | C | 0.46 | 1.87E-44 | ----- | 0 | 0.47 | 0.5 | 8.07E-01 | +- | 0 |
| 10 | rs1599166 | 62163729 | *ANK3* | A | G | 0.78 | 3.41E-18 | +++++ | 1.9 | NA | NA | NA | NA | NA |
| 10 | rs4262652 | 74127853 | *ANAPC16* | A | G | 0.64 | 1.83E-17 | +++++ | 0 | 0.14 | 0.12 | 5.78E-01 | ++ | 0 |
| 10 | rs7073435 | 75886512 | *VCL* | T | C | 0.27 | 2.42E-09 | +++++ | 0 | 0.65 | 0.65 | 5.41E-01 | +- | 13.56 |
| 10 | rs7895108 | 79061458 | *KCNMA1* | T | G | 0.37 | 2.96E-69 | ----- | 76.7 | 0.14 | 0.15 | NA | -NA | NA |
| 10 | rs4517452 | 86021024 | *GHITM* | T | C | 0.63 | 9.53E-71 | +++++ | 63.7 | 0.7 | 0.7 | NA | +NA | NA |
| 10 | rs10887652 | 88511237 | *BMPR1A* | C | G | 0.63 | 3.22E-08 | ----- | 0 | 0.27 | 0.27 | 4.45E-01 | -- | 0 |
| 10 | rs3781196 | 90042154 | *RNLS* | T | G | 0.23 | 4.6E-32 | +++++ | 0 | 0.17 | 0.16 | 8.03E-02 | +- | 0 |
| 10 | rs4933627 | 92712252 | *ANKRD1* | A | G | 0.42 | 2E-08 | +++++ | 0 | NA | NA | NA | NA | NA |
| 10 | rs12778014 | 94950273 | *EXOC6* | A | G | 0.34 | 2.77E-23 | +++++ | 51.1 | 0.07 | 0.08 | 3.68E-01 | +- | 0 |
| 10 | rs807037 | 102824349 | *ERLIN1* | C | G | 0.66 | 3.07E-22 | ----- | 0 | 0.43 | 0.43 | 2.68E-03 | -- | 0 |
| 10 | rs2501571 | 111855439 | *XPNPEP1* | T | G | 0.18 | 2.04E-14 | +++++ | 31.5 | NA | NA | NA | NA | NA |
| 10 | rs56299331 | 114788436 | *VTI1A* | T | C | 0.2 | 9.38E-46 | ----- | 70.8 | NA | NA | NA | NA | NA |
| 10 | rs11196996 | 116694631 | *TRUB1* | T | C | 0.41 | 2.36E-08 | ----- | 0 | 0.03 | 0.04 | 8.64E-01 | -+ | 48.92 |
| 10 | rs3750847 | 124215421 | *PLEKHA1* | T | C | 0.22 | 5.72E-23 | ----- | 43.2 | 0.6 | 0.56 | 7.89E-01 | ++ | 0 |
| 10 | rs1152698 | 126807285 | *CTBP2* | C | G | 0.17 | 8.33E-17 | ----- | 16.4 | NA | NA | NA | NA | NA |
| 11 | rs113626448 | 589392 | *PHRF1* | T | G | 0.95 | 9.54E-09 | -?--- | 0 | NA | NA | NA | NA | NA |
| 11 | rs10839545 | 1744093 | *MOB2* | A | G | 0.46 | 3.68E-13 | +++++ | 17.9 | NA | NA | NA | NA | NA |
| 11 | rs12291662 | 14342949 | *SPON1* | T | C | 0.65 | 3.38E-11 | ----- | 0 | 0.63 | 0.61 | 3.30E-01 | -+ | 0 |
| 11 | rs1550870 | 18751041 | *IGSF22* | T | C | 0.47 | 2.02E-27 | +++++ | 61.8 | NA | NA | NA | NA | NA |
| 11 | rs6484385 | 28668416 | *METTL15* | T | C | 0.63 | 6.6E-16 | +++++ | 12.9 | 0.21 | 0.2 | NA | +NA | NA |
| 11 | rs511217 | 30029948 | *KCNA4* | A | T | 0.74 | 2.3E-49 | ----- | 0 | 0.69 | 0.73 | 7.04E-01 | ++ | 0 |
| 11 | rs11606250 | 40149300 | *LRRC4C* | A | G | 0.17 | 1.38E-175 | ----- | 88.6 | 0.28 | 0.33 | 7.01E-02 | -- | 0 |
| 11 | rs11037404 | 43296794 | *API5* | T | C | 0.38 | 2.37E-24 | +++++ | 31.5 | 0.28 | 0.26 | 3.24E-01 | +- | 18.82 |
| 11 | rs55681357 | 45687366 | *CHST1* | T | C | 0.36 | 1.4E-10 | ----- | 58.9 | 0.16 | 0.16 | 3.29E-01 | +- | 34.1 |
| 11 | rs7928419 | 47392114 | *DDB2* | A | G | 0.67 | 1.94E-14 | ----- | 38.4 | NA | NA | NA | NA | NA |

**Table S11. Comparison of SNPs previously associated with myopia in European adults and their effects in our children cohorts**

| | | | | | | Meta-GWAS of European adult cohorts[a] | | | | Meta-GWAS of our Chinese children corhorts | | | | |
|---|---|---|---|---|---|---|---|---|---|---|---|---|---|---|
| CHR | SNP | BP | Nearest genes | EA | NEA | EAF of European adults | P | Direction | Heterogeneity $I^2$ (%) | EAF of HKCES | EAF of LAMP | P | Direction | Heterogeneity $I^2$ (%) |
| 11 | rs198443 | 61505696 | *SLC15A3* | T | G | 0.44 | 2.18E-24 | ----- | 71.4 | 0.79 | 0.81 | 8.93E-01 | ++ | 0 |
| 11 | rs620088 | 65508986 | *SLC25A45* | A | G | 0.35 | 3.21E-11 | +++++ | 36.1 | 0.25 | 0.22 | 5.52E-01 | +- | 10.26 |
| 11 | rs2276118 | 67288594 | *RPS6KB2* | T | C | 0.56 | 2.93E-13 | +++++ | 0 | 0.69 | 0.69 | 8.65E-01 | +- | 29.66 |
| 11 | rs493786 | 69329674 | *CCND1* | T | C | 0.33 | 5.45E-09 | +++++ | 0 | NA | NA | NA | NA | NA |
| 11 | rs1278092 | 73233855 | *P2RY6* | T | C | 0.39 | 1.4E-13 | ----- | 0 | NA | NA | NA | NA | NA |
| 11 | rs2155413 | 84634790 | *ANKRD42* | A | C | 0.47 | 1.97E-53 | ----- | 85.7 | NA | NA | NA | NA | NA |
| 11 | rs535888 | 92407574 | *FAT3* | T | C | 0.49 | 2.86E-10 | +++++ | 19.5 | 0.39 | 0.4 | 1.20E-01 | +- | 74.76 |
| 11 | rs4753693 | 95293753 | *KDM4E* | A | T | 0.51 | 2.85E-10 | ----- | 0 | NA | NA | NA | NA | NA |
| 11 | rs10895869 | 105600358 | *GRIA4* | A | C | 0.37 | 4.28E-39 | ----- | 66.2 | 0.33 | 0.38 | 4.16E-02 | -- | 66.02 |
| 11 | rs2028155 | 108267430 | *CUL5* | A | G | 0.45 | 3.53E-19 | ----- | 46.8 | 0.6 | 0.62 | 1.65E-01 | ++ | 51.28 |
| 11 | rs7947524 | 117671091 | *APOC3* | T | C | 0.5 | 2.95E-34 | ----- | 8.9 | 0.31 | 0.27 | 2.00E-01 | -+ | 0 |
| 11 | rs1029278 | 123421402 | *GRAMD1B* | A | G | 0.34 | 4.57E-21 | ----- | 0 | NA | NA | NA | NA | NA |
| 11 | rs6590199 | 126235667 | *ST3GAL4* | A | G | 0.16 | 6.21E-09 | ----- | 0 | 0.3 | 0.29 | 3.62E-01 | -- | 45.98 |
| 11 | rs10219187 | 129103092 | *ETS1* | T | C | 0.68 | 1.17E-17 | ----- | 0 | 0.71 | 0.69 | 3.95E-01 | -- | 0 |
| 11 | rs1790165 | 131928971 | *NTM* | A | C | 0.42 | 7.47E-29 | +++++ | 75.4 | 0.19 | 0.17 | 1.28E-01 | ++ | 0 |
| 12 | rs10848650 | 377207 | *SLC6A13* | C | G | 0.59 | 1.55E-08 | ----- | 0 | 0.17 | 0.14 | 6.10E-01 | -- | 0 |
| 12 | rs6489568 | 4930100 | *KCNA6* | A | G | 0.21 | 1.61E-08 | ----- | 0 | 0.37 | 0.4 | 8.80E-01 | -+ | 0 |
| 12 | rs5442 | 6954864 | *GNB3* | A | G | 0.07 | 2.64E-62 | -?--- | 84.7 | NA | NA | NA | NA | NA |
| 12 | rs10842971 | 9303296 | *A2ML1* | A | T | 0.69 | 1.34E-40 | +++++ | 0 | 0.92 | 0.91 | 6.98E-01 | -+ | 14.42 |
| 12 | rs7966177 | 10879132 | *YBX3* | T | C | 0.47 | 5.05E-09 | ----- | 42.7 | 0.23 | 0.2 | 8.33E-02 | ++ | 85.43 |
| 12 | rs17834080 | 14039124 | *GPR19* | T | G | 0.27 | 4.43E-34 | ----- | 0 | NA | NA | NA | NA | NA |
| 12 | rs6487108 | 20736762 | *PDE3A* | A | G | 0.29 | 2.95E-08 | ----- | 62 | 0.19 | 0.19 | 9.09E-01 | +- | 71.88 |
| 12 | rs144311425 | 22555028 | *ST8SIA1* | T | C | 0.09 | 8.8E-26 | ----- | 40.7 | 0.05 | 0.04 | 2.61E-01 | -- | 8.48 |
| 12 | rs4964008 | 26776560 | *ITPR2* | C | G | 0.66 | 2.44E-13 | ----- | 10 | NA | NA | NA | NA | NA |
| 12 | rs1489081 | 29063122 | *FAR2* | A | G | 0.47 | 2.98E-14 | ----- | 53.6 | 0.34 | 0.37 | 5.27E-01 | -- | 0 |
| 12 | rs7311970 | 31746047 | *DENND5B* | A | C | 0.62 | 8.09E-10 | ----- | 0 | 0.58 | 0.61 | 4.09E-01 | -+ | 65.5 |
| 12 | rs11181913 | 43574200 | *GXYLT1* | A | G | 0.91 | 2.75E-11 | +++++ | 0 | 0.93 | 0.92 | 9.17E-01 | +- | 66.46 |
| 12 | rs1468993 | 46155145 | *ARID2* | T | C | 0.49 | 2.42E-29 | +++++ | 68.5 | NA | NA | NA | NA | NA |
| 12 | rs3138142 | 56115585 | *CBX5* | T | C | 0.23 | 5.71E-174 | +++++ | 86.2 | 0.15 | 0.11 | 4.68E-01 | ++ | 0 |
| 12 | rs11178450 | 71233908 | *PTPRR* | T | C | 0.22 | 5.12E-26 | +++++ | 55.8 | 0.43 | 0.4 | 6.46E-02 | ++ | 0 |
| 12 | rs1280632 | 91622254 | *DCN* | T | C | 0.89 | 3.67E-09 | +++++ | 0 | NA | NA | NA | NA | NA |
| 12 | rs117821803 | 96194586 | *NTN4* | T | C | 0.19 | 1.2E-19 | +++++ | 0 | 0.03 | 0.03 | 1.71E-01 | -+ | 0 |
| 12 | rs7136446 | 102838515 | *NUP37* | T | C | 0.59 | 2.54E-10 | ----- | 0 | 0.82 | 0.84 | 7.79E-01 | -+ | 9.32 |
| 12 | rs17218455 | 106716441 | *CKAP4* | T | C | 0.17 | 1.14E-12 | ----- | 12.5 | 0.02 | 0.02 | 6.31E-01 | -- | 0 |
| 12 | rs7315286 | 108979990 | *TMEM119* | A | G | 0.55 | 1.51E-08 | +++++ | 26.1 | 0.13 | 0.16 | NA | +NA | NA |
| 12 | rs1732579 | 115716844 | *NA* | A | G | 0.65 | 3.85E-09 | ----- | 0 | 0.96 | 0.98 | 3.72E-01 | -+ | 82.04 |
| 12 | rs4766878 | 118236577 | *KSR2* | T | C | 0.45 | 8.41E-14 | ----- | 56.5 | 0.73 | 0.73 | 5.99E-01 | +- | 0 |
| 12 | rs11065286 | 121271734 | *COQ5* | T | C | 0.45 | 8.47E-11 | ----- | 0 | NA | NA | NA | NA | NA |

**Table S11. Comparison of SNPs previously associated with myopia in European adults and their effects in our children cohorts**

| | | | | | | Meta-GWAS of European adult cohorts[a] | | | | Meta-GWAS of our Chinese children corhorts | | | | |
|---|---|---|---|---|---|---|---|---|---|---|---|---|---|---|
| CHR | SNP | BP | Nearest genes | EA | NEA | EAF of European adults | P | Direction | Heterogeneity $I^2$ (%) | EAF of HKCES | EAF of LAMP | P | Direction | Heterogeneity $I^2$ (%) |
| 12 | rs7307053 | 124494540 | *ZNF664* | T | C | 0.32 | 3.74E-08 | ----- | 0 | 0.07 | 0.07 | 5.73E-01 | -- | 0 |
| 12 | rs12308700 | 131157564 | *RIMBP2* | A | G | 0.42 | 1.24E-13 | ----- | 71.2 | 0.31 | 0.32 | 8.60E-02 | -- | 0 |
| 12 | rs905224 | 133684321 | *ANKLE2* | T | G | 0.56 | 1.33E-12 | ----- | 0 | NA | NA | NA | NA | NA |
| 13 | rs587289 | 21596183 | *LATS2* | A | G | 0.28 | 9.68E-12 | +++++ | 0 | NA | NA | NA | NA | NA |
| 13 | rs34961350 | 28991902 | *FLT1* | C | G | 0.79 | 4.13E-32 | ----- | 0 | 0.72 | 0.72 | 6.50E-01 | +- | 28.23 |
| 13 | rs4769824 | 30883339 | *KATNAL1* | T | C | 0.44 | 1.32E-11 | ----- | 0 | 0.13 | 0.13 | 5.84E-01 | ++ | 0 |
| 13 | rs353 | 32454349 | *EEF1DP3* | A | G | 0.94 | 9.73E-13 | +++++ | 0 | NA | NA | NA | NA | NA |
| 13 | rs45502300 | 36246512 | *MIR548F5* | A | G | 0.97 | 2.17E-23 | +++++ | 0 | NA | NA | NA | NA | NA |
| 13 | rs9526001 | 45339106 | *TSC22D1* | T | C | 0.27 | 5E-09 | ----- | 1.1 | 0.36 | 0.37 | 6.63E-01 | -+ | 0 |
| 13 | rs9535263 | 50137363 | *SETDB2* | A | T | 0.7 | 1.08E-36 | ----- | 13.6 | 0.65 | 0.65 | 2.79E-01 | ++ | 0 |
| 13 | rs9526736 | 51799497 | *GUCY1B2* | T | C | 0.57 | 9.23E-09 | +++++ | 4.7 | 0.83 | 0.82 | 2.86E-01 | -- | 0 |
| 13 | rs36080062 | 67726830 | *PCDH9* | C | G | 0.2 | 7.14E-13 | ----- | 0 | NA | NA | NA | NA | NA |
| 13 | rs75012440 | 74631240 | *KLF12* | A | G | 0.14 | 6.22E-10 | ----- | 50.6 | 0.06 | 0.07 | 6.27E-01 | ++ | 0 |
| 13 | rs4052505 | 80512487 | *NA* | T | C | 0.19 | 8.13E-20 | ----- | 23.7 | 0.32 | 0.34 | 9.35E-02 | -- | 0 |
| 13 | rs9547035 | 85573496 | *NA* | T | G | 0.73 | 7.12E-32 | +++++ | 59.8 | 0.59 | 0.59 | 8.55E-01 | -+ | 0 |
| 13 | rs1328371 | 93912785 | *GPC6* | T | C | 0.49 | 3.59E-23 | ----- | 0 | NA | NA | NA | NA | NA |
| 13 | rs9562057 | 96897856 | *HS6ST3* | T | C | 0.1 | 6.06E-09 | ----- | 0 | 0.32 | 0.29 | 1.44E-02 | -- | 0 |
| 13 | rs724154 | 100673616 | *SLC15A1* | A | G | 0.45 | 1.92E-76 | ----- | 71 | 0.18 | 0.2 | 2.97E-01 | ++ | 0 |
| 13 | rs9521975 | 111539925 | *ANKRD10* | A | G | 0.41 | 5.56E-09 | ----- | 0 | 0.14 | 0.14 | 1.84E-01 | -- | 20.8 |
| 14 | rs4981370 | 21779495 | *RPGRIP1* | A | G | 0.49 | 5.67E-09 | -+--- | 43.9 | 0.47 | 0.43 | 4.67E-01 | +- | 72.04 |
| 14 | rs34354104 | 24707479 | *NEDD8-MDP1* | A | G | 0.05 | 4.84E-10 | +++++ | 0 | NA | NA | NA | NA | NA |
| 14 | rs12883788 | 33303540 | *AKAP6* | T | C | 0.45 | 2.11E-33 | +++++ | 0 | 0.28 | 0.27 | 4.29E-01 | -- | 0 |
| 14 | rs34217772 | 42273570 | *LRFN5* | C | G | 0.81 | 1.18E-39 | +++++ | 74 | NA | 0.99 | NA | NA+ | NA |
| 14 | rs9788504 | 51270267 | *SAV1* | C | G | 0.59 | 1.07E-11 | +++++ | 32.4 | NA | NA | NA | NA | NA |
| 14 | rs2855530 | 54421917 | *BMP4* | C | G | 0.5 | 1.92E-59 | ----- | 56.4 | 0.47 | 0.47 | 2.98E-01 | -- | 39.67 |
| 14 | rs928109 | 57279583 | *OTX2* | T | C | 0.48 | 6.15E-11 | ----- | 0 | 0.36 | 0.36 | 7.50E-01 | -+ | 0 |
| 14 | rs1254314 | 60896434 | *PPM1A* | C | G | 0.29 | 1.98E-40 | ----- | 60.5 | 0.69 | 0.72 | 4.04E-01 | +- | 72.37 |
| 14 | rs34096978 | 63784378 | *GPHB5* | A | G | 0.04 | 1.03E-11 | +++++ | 0 | NA | NA | NA | NA | NA |
| 14 | rs3211166 | 69703158 | *TMEM229B* | A | G | 0.69 | 6.16E-11 | +++++ | 0 | NA | NA | NA | NA | NA |
| 14 | rs73294488 | 74949801 | *DPF3* | T | C | 0.99 | 6.52E-25 | -?--- | 56.6 | NA | NA | NA | NA | NA |
| 14 | rs56284621 | 89429230 | *TTC8* | T | C | 0.8 | 2.82E-19 | +++++ | 62.5 | 0.62 | 0.63 | 2.17E-01 | +- | 9.97 |
| 14 | rs12896393 | 92625903 | *TRIP11* | A | G | 0.84 | 4.49E-24 | ----- | 75.9 | 0.49 | 0.49 | 7.18E-01 | +- | 0 |
| 14 | rs7144361 | 96103184 | *GLRX5* | T | C | 0.08 | 9.31E-11 | +++++ | 0 | 0.11 | 0.11 | 1.74E-01 | ++ | 0 |
| 14 | rs4905998 | 101181781 | *WDR25* | A | G | 0.32 | 3.19E-10 | +++++ | 25.4 | 0.6 | 0.59 | 9.75E-01 | -+ | 0 |
| 14 | rs35337422 | 104407243 | *ZNF839* | A | C | 0.86 | 9.67E-21 | +++++ | 53.8 | NA | NA | NA | NA | NA |
| 15 | rs11161294 | 26607405 | *ATP10A3* | A | G | 0.13 | 4.18E-11 | +++++ | 25.6 | 0.18 | 0.2 | 9.88E-01 | -+ | 0 |
| 15 | rs79406658 | 27988096 | *OCA2* | A | C | 0.43 | 1.37E-15 | +++++ | 0 | 0.04 | 0.05 | 4.58E-01 | -- | 0 |
| 15 | rs3784589 | 31294714 | *TRPM1* | A | C | 0.05 | 4.74E-10 | +++++ | 64.6 | NA | NA | NA | NA | NA |

**Table S11. Comparison of SNPs previously associated with myopia in European adults and their effects in our children cohorts**

| | | | | | | Meta-GWAS of European adult cohorts[a] | | | | Meta-GWAS of our Chinese children corhorts | | | | |
|---|---|---|---|---|---|---|---|---|---|---|---|---|---|---|
| CHR | SNP | BP | Nearest genes | EA | NEA | EAF of European adults | P | Direction | Heterogeneity $I^2$ (%) | EAF of HKCES | EAF of LAMP | P | Direction | Heterogeneity $I^2$ (%) |
| 15 | rs524952 | 35005886 | *GOLGA8B* | A | T | 0.49 | 2.49E-245 | ----- | 94.1 | 0.56 | 0.52 | 3.65E-02 | ++ | 0 |
| 15 | rs35636169 | 36594557 | *NA* | T | C | 0.63 | 3.33E-09 | ----- | 14.4 | 0.77 | 0.76 | 8.50E-01 | +- | 60.39 |
| 15 | rs1058734 | 40862064 | *BAHD1* | T | C | 0.46 | 2.83E-11 | +++++ | 50.9 | 0.09 | 0.1 | 2.30E-01 | +- | 48.17 |
| 15 | rs2017765 | 48749703 | *FBN1* | T | C | 0.13 | 3.3E-24 | +++++ | 25.1 | NA | NA | NA | NA | NA |
| 15 | rs8042462 | 51045616 | *USP50* | A | G | 0.03 | 5.53E-12 | +++++ | 35.7 | NA | NA | NA | NA | NA |
| 15 | rs1912409 | 56158495 | *NEDD4* | T | C | 0.6 | 1.72E-09 | ----- | 0 | 0.51 | 0.52 | 7.02E-01 | +- | 71.62 |
| 15 | rs55870008 | 61489314 | *RORA* | A | G | 0.45 | 5.55E-09 | +++++ | 50.6 | 0.68 | 0.64 | 4.26E-01 | ++ | 33.76 |
| 15 | rs7162310 | 63571234 | *TPM1* | T | C | 0.21 | 5.98E-36 | +++++ | 68.1 | 0.47 | 0.46 | 4.93E-01 | -+ | 74.98 |
| 15 | rs8042545 | 66992821 | *LCTL* | A | G | 0.25 | 2.04E-12 | ----- | 61 | 0.05 | 0.06 | 1.96E-01 | ++ | 0 |
| 15 | rs28588430 | 74223430 | *LOXL1* | C | G | 0.49 | 1.61E-18 | +++++ | 30.7 | 0.1 | 0.09 | 5.53E-02 | ++ | 0 |
| 15 | rs4886850 | 77404160 | *PEAK1* | C | G | 0.76 | 1.08E-09 | ----- | 44.6 | NA | NA | NA | NA | NA |
| 15 | rs1961579 | 79380516 | *CHRNA3* | A | G | 0.42 | 7.72E-84 | ----- | 79.2 | 0.51 | 0.53 | 1.03E-01 | -- | 0 |
| 15 | rs8039459 | 82320426 | *MEX3B* | C | G | 0.31 | 2.52E-27 | +++++ | 0 | 0.52 | 0.53 | 1.75E-01 | -- | 0 |
| 15 | rs12440698 | 85825906 | *AKAP13* | A | G | 0.88 | 4.69E-08 | ----- | 0 | NA | NA | NA | NA | NA |
| 15 | rs4502168 | 93744204 | *SLCO3A1* | T | C | 0.28 | 5.67E-12 | +++++ | 0 | 0.27 | 0.28 | 6.30E-01 | -+ | 0 |
| 15 | rs10520789 | 96141867 | *MCTP2* | A | G | 0.12 | 1E-11 | +++++ | 31.9 | 0.02 | 0.02 | 9.27E-01 | -+ | 0 |
| 15 | rs2654980 | 99504843 | *IGF1R* | T | C | 0.25 | 3.71E-08 | +++++ | 0 | 0.03 | 0.02 | 9.00E-01 | +- | 0 |
| 16 | rs710902 | 1376510 | *AXIN1* | T | C | 0.13 | 6.4E-18 | +?+++ | 0 | NA | NA | NA | NA | NA |
| 16 | rs17648524 | 7459683 | *RBFOX1* | C | G | 0.37 | 1.35E-180 | ----- | 86.8 | 0.07 | 0.07 | 1.15E-01 | -- | 53.65 |
| 16 | rs1868289 | 10215813 | *GRIN2A* | T | G | 0.68 | 1.13E-17 | ----- | 50.6 | 0.55 | 0.53 | 7.00E-01 | -- | 0 |
| 16 | rs2606558 | 18821191 | *RPS15A* | C | G | 0.46 | 8.96E-10 | ----- | 0.3 | NA | NA | NA | NA | NA |
| 16 | rs497523 | 28577931 | *GSG1L* | T | C | 0.65 | 3E-09 | +++-+ | 74.6 | NA | NA | NA | NA | NA |
| 16 | rs62026817 | 55492932 | *IRX5* | A | G | 0.23 | 7.43E-10 | -+--- | 60.8 | 0.18 | 0.17 | 6.70E-01 | -+ | 66.32 |
| 16 | rs255049 | 68013471 | *SLC9A5* | T | C | 0.81 | 2.45E-14 | +++++ | 58.7 | 0.9 | 0.93 | 8.40E-01 | -- | 0 |
| 16 | rs28533147 | 73195849 | *MARVELD3* | T | G | 0.82 | 4.38E-10 | +++++ | 46.3 | NA | NA | NA | NA | NA |
| 16 | rs13339407 | 78156295 | *WWOX* | A | G | 0.69 | 9.45E-10 | ----- | 0 | NA | NA | NA | NA | NA |
| 16 | rs4635359 | 80537760 | *MAF* | A | C | 0.74 | 1.71E-28 | ----- | 0 | 0.41 | 0.47 | 6.10E-01 | -- | 0 |
| 16 | rs1054521 | 87436720 | *MAP1LC3B* | T | G | 0.94 | 5.53E-09 | +?+++ | 75.1 | NA | NA | NA | NA | NA |
| 17 | rs9892466 | 3969527 | *ATP2A3* | A | T | 0.33 | 6.47E-14 | ----- | 0 | 0.16 | 0.16 | 3.29E-01 | ++ | 65.99 |
| 17 | rs9217 | 7363088 | *NLGN2* | T | C | 0.64 | 3E-22 | ----- | 72.6 | 0.79 | 0.81 | 3.76E-01 | ++ | 0 |
| 17 | rs2908972 | 11407259 | *SHISA6* | A | T | 0.4 | 3.71E-84 | ----- | 77.3 | 0.54 | 0.58 | 1.26E-01 | ++ | 0 |
| 17 | rs115152181 | 14136125 | *COX10* | A | T | 0.43 | 3.96E-27 | ----- | 25.9 | NA | NA | NA | NA | NA |
| 17 | rs62070229 | 31227593 | *MYO1D* | A | G | 0.81 | 7.04E-44 | +++++ | 68.7 | NA | NA | NA | NA | NA |
| 17 | rs35497503 | 37620627 | *STAC2* | T | C | 0.75 | 1.11E-24 | +++++ | 21.6 | 0.68 | 0.68 | 7.71E-01 | -+ | 27.39 |
| 17 | rs76324150 | 43973233 | *ARHGAP27* | T | C | 0.22 | 8.24E-16 | +++++ | 33.5 | NA | NA | NA | NA | NA |
| 17 | rs7222840 | 47280915 | *B4GALNT2* | T | C | 0.32 | 1.27E-44 | +++++ | 74.4 | NA | NA | NA | NA | NA |
| 17 | rs8075811 | 54735307 | *NOG* | A | G | 0.36 | 7.34E-42 | +++++ | 76.4 | 0.35 | 0.31 | 4.71E-01 | ++ | 55.1 |
| 17 | rs3785837 | 59468942 | *BCAS3* | A | G | 0.77 | 3.36E-21 | -?--- | 0 | NA | NA | NA | NA | NA |

**Table S11. Comparison of SNPs previously associated with myopia in European adults and their effects in our children cohorts**

| | | | | | | Meta-GWAS of European adult cohorts[a] | | | | Meta-GWAS of our Chinese children corhorts | | | | |
|---|---|---|---|---|---|---|---|---|---|---|---|---|---|---|
| CHR | SNP | BP | Nearest genes | EA | NEA | EAF of European adults | P | Direction | Heterogeneity $I^2$ (%) | EAF of HKCES | EAF of LAMP | P | Direction | Heterogeneity $I^2$ (%) |
| 17 | rs1991401 | 62502435 | *KCNH6* | A | G | 0.68 | 1.81E-13 | +++++ | 20.4 | 0.6 | 0.59 | 8.80E-01 | +- | 0 |
| 17 | rs9895291 | 63550797 | *AXIN2* | A | C | 0.16 | 1.4E-08 | ----- | 27.6 | 0.08 | 0.08 | 5.32E-01 | +- | 0 |
| 17 | rs62084693 | 66036110 | *BPTF* | T | C | 0.75 | 7.11E-12 | ----- | 0 | 0.28 | 0.28 | 6.21E-01 | -- | 0 |
| 17 | rs4793501 | 68718734 | *NA* | T | C | 0.58 | 2.57E-24 | ----- | 83.3 | 0.39 | 0.4 | 6.27E-01 | +- | 78.24 |
| 17 | rs448203 | 75495065 | *MGAT5B* | T | C | 0.6 | 4.35E-14 | +++++ | 12.9 | 0.07 | 0.05 | 3.86E-01 | +- | 0 |
| 17 | rs9747347 | 79606820 | *TMEM105* | T | C | 0.36 | 2.22E-50 | ----- | 86.1 | NA | NA | NA | NA | NA |
| 18 | rs11659457 | 6461151 | *L3MBTL4* | A | G | 0.41 | 3.63E-14 | +++++ | 19.9 | 0.51 | 0.53 | 4.56E-01 | -+ | 0 |
| 18 | rs11564403 | 25662803 | *CDH2* | A | T | 0.28 | 2.48E-15 | ----- | 39.1 | NA | NA | NA | NA | NA |
| 18 | rs1427039 | 35347100 | *CELF4* | T | C | 0.66 | 1.49E-08 | ----- | 0 | NA | 0.7 | NA | NA- | NA |
| 18 | rs10163854 | 39162524 | *NA* | C | G | 0.9 | 5.7E-12 | ----- | 32.4 | 0.99 | 0.98 | 6.13E-01 | -+ | 0 |
| 18 | rs16978339 | 42886236 | *SETBP1* | T | G | 0.84 | 2.88E-36 | ----- | 0 | 0.88 | 0.89 | 4.37E-01 | -- | 0 |
| 18 | rs12965607 | 47391025 | *DYM* | T | G | 0.85 | 3.89E-60 | +++++ | 2.9 | NA | NA | NA | NA | NA |
| 18 | rs41396445 | 53242137 | *TCF4* | A | C | 0.35 | 4.14E-12 | ++++- | 66.8 | 0.4 | 0.37 | 9.82E-01 | +- | 13.53 |
| 18 | rs9646588 | 62990567 | *NA* | A | G | 0.37 | 9.42E-12 | ----- | 0 | 0.45 | 0.46 | 1.25E-01 | -- | 61.54 |
| 18 | rs734559 | 72179579 | *CNDP2* | A | G | 0.23 | 2.41E-14 | +++++ | 80.1 | 0.3 | 0.29 | 2.37E-01 | -+ | 54.83 |
| 18 | rs72973714 | 74076020 | *ZNF516* | T | C | 0.07 | 3.06E-08 | +++++ | 0 | NA | NA | NA | NA | NA |
| 18 | rs8096658 | 77156537 | *NFATC1* | C | G | 0.51 | 5.19E-10 | -?--- | 49.8 | 0.63 | 0.65 | 8.65E-01 | ++ | 0 |
| 19 | rs123698 | 807442 | *PPAP2C* | C | G | 0.6 | 1.19E-11 | +?+++ | 0 | NA | NA | NA | NA | NA |
| 19 | rs75488612 | 4037322 | *PIAS4* | A | G | 0.1 | 1.7E-09 | -?--- | 0 | NA | NA | NA | NA | NA |
| 19 | rs10416763 | 8232734 | *FBN3* | T | C | 0.45 | 3.8E-16 | +++++ | 0 | 0.45 | 0.43 | 1.40E-02 | ++ | 8.38 |
| 19 | rs113628353 | 13201606 | *NFIX,LYL1* | A | G | 0.11 | 1.93E-11 | +++++ | 0 | NA | NA | NA | NA | NA |
| 19 | rs75452709 | 17455453 | *ANO8* | C | G | 0.5 | 3.9E-08 | +?+++ | 0 | NA | NA | NA | NA | NA |
| 19 | rs2074302 | 19381728 | *RFXANK* | C | G | 0.16 | 3.99E-29 | ----- | 0 | 0.02 | 0.02 | NA | -NA | NA |
| 19 | rs73035723 | 31779696 | *TSHZ3* | A | G | 0.11 | 1.32E-10 | ----- | 71.4 | NA | NA | NA | NA | NA |
| 19 | rs807479 | 36253317 | *UPK1A* | T | C | 0.47 | 1.2E-10 | -?--- | 45.7 | 0.46 | 0.45 | 7.38E-01 | -+ | 0 |
| 19 | rs57654288 | 38078771 | *ZNF793* | A | G | 0.38 | 7.04E-09 | +++++ | 0 | 0.08 | 0.06 | 3.49E-01 | -- | 76.44 |
| 19 | rs429358 | 45411941 | *PVRL2* | T | C | 0.85 | 3.03E-17 | ----- | 59.7 | 0.91 | 0.92 | 2.54E-01 | +- | 29.21 |
| 19 | rs75601727 | 48531806 | *CCDC9* | A | G | 0.1 | 1.11E-27 | +++++ | 0 | 0.05 | 0.05 | 2.15E-01 | ++ | 0 |
| 20 | rs11087326 | 2127684 | *STK35* | T | C | 0.25 | 1.01E-12 | ----- | 53.6 | 0.93 | 0.94 | 9.44E-01 | -+ | 0 |
| 20 | rs41281858 | 3209083 | *LZTS3* | A | G | 0.13 | 5.81E-11 | +++++ | 37 | NA | NA | NA | NA | NA |
| 20 | rs7272323 | 4756691 | *RASSF2* | C | G | 0.6 | 9.41E-11 | +++++ | 0 | 0.27 | 0.24 | 5.26E-01 | ++ | 20.99 |
| 20 | rs235770 | 6761765 | *BMP2* | T | C | 0.39 | 3.9E-31 | ----- | 13.4 | 0.29 | 0.31 | 3.86E-01 | -- | 0 |
| 20 | rs6136407 | 18529855 | *DZANK1* | A | C | 0.61 | 2.14E-08 | +++++ | 25.1 | 0.87 | 0.86 | 2.30E-02 | ++ | 0 |
| 20 | rs6137371 | 21485247 | *NKX2-2* | T | C | 0.58 | 2.68E-09 | +++++ | 0 | 0.53 | 0.57 | 6.89E-01 | -+ | 21.05 |
| 20 | rs6050351 | 25118271 | *VSX1* | T | C | 0.18 | 4.59E-08 | +++++ | 7.2 | 0.42 | 0.42 | 5.76E-02 | -- | 0 |
| 20 | rs2378078 | 32714728 | *CHMP4B* | T | C | 0.64 | 4.84E-19 | ----- | 58.6 | NA | NA | NA | NA | NA |
| 20 | rs6120993 | 34185161 | *GDF5* | T | G | 0.14 | 1.59E-20 | ----- | 30.8 | 0.15 | 0.19 | 6.25E-01 | +- | 0 |
| 20 | rs6102322 | 39872768 | *TOP1* | T | C | 0.31 | 7.27E-10 | ----- | 0 | 0.78 | 0.82 | 1.17E-01 | -- | 10.4 |

**Table S11. Comparison of SNPs previously associated with myopia in European adults and their effects in our children cohorts**

| | | | | | | Meta-GWAS of European adult cohorts[a] | | | | Meta-GWAS of our Chinese children corhorts | | | | |
|---|---|---|---|---|---|---|---|---|---|---|---|---|---|---|
| CHR | SNP | BP | Nearest genes | EA | NEA | EAF of European adults | P | Direction | Heterogeneity $I^2$ (%) | EAF of HKCES | EAF of LAMP | P | Direction | Heterogeneity $I^2$ (%) |
| 20 | rs8121964 | 42489757 | *TOX2* | A | G | 0.25 | 1.45E-08 | ----- | 0 | NA | NA | NA | NA | NA |
| 20 | rs6099283 | 55375861 | *TFAP2C* | A | G | 0.25 | 3.06E-09 | ----- | 0 | 0.35 | 0.33 | 3.53E-01 | ++ | 1.53 |
| 20 | rs6070941 | 58354388 | *PHACTR3* | C | G | 0.83 | 1.55E-10 | +++++ | 0 | 0.75 | 0.76 | 5.86E-01 | -+ | 42.36 |
| 20 | rs2259858 | 62346459 | *BHLHE23* | A | T | 0.75 | 1.03E-14 | +++++ | 51.4 | 0.41 | 0.37 | 1.13E-01 | +- | 48.4 |
| 21 | rs2229741 | 16340289 | *NRIP1* | T | C | 0.42 | 1.34E-29 | ----- | 43.4 | 0.79 | 0.81 | 4.63E-01 | +- | 53.05 |
| 21 | rs117123217 | 30910628 | *GRIK1* | A | G | 0.04 | 1.35E-10 | ----- | 7.9 | NA | NA | NA | NA | NA |
| 21 | rs74848904 | 36612241 | *RUNX1* | A | C | 0.1 | 1.66E-08 | +++++ | 0 | NA | NA | NA | NA | NA |
| 21 | rs2212597 | 39983404 | *ERG* | T | C | 0.82 | 5.49E-11 | ----- | 3.3 | 0.72 | 0.69 | 4.06E-01 | -- | 0 |
| 21 | rs7277023 | 41915359 | *DSCAM* | A | G | 0.98 | 4.63E-08 | ----- | 9.8 | 0.9 | 0.91 | 9.05E-02 | -- | 0 |
| 21 | rs11700909 | 45118701 | *HSF2BP* | C | G | 0.59 | 2.56E-10 | +++++ | 52.7 | NA | NA | NA | NA | NA |
| 21 | rs73157695 | 47371947 | *PCBP3* | A | G | 0.3 | 1.91E-36 | ----- | 30.6 | 0.17 | 0.2 | 8.07E-01 | +- | 40.62 |
| 22 | rs9608575 | 27486722 | *MN1* | A | G | 0.67 | 2.32E-11 | ----+ | 28.2 | NA | NA | NA | NA | NA |
| 22 | rs2097352 | 30236444 | *ASCC2* | A | T | 0.75 | 2.46E-09 | +++++ | 0 | 0.86 | 0.89 | 2.26E-02 | ++ | 0 |
| 22 | rs9606973 | 32900527 | *BPIFC* | A | T | 0.21 | 5.91E-13 | ----- | 0 | 0.14 | 0.12 | 9.97E-02 | -- | 0 |
| 22 | rs13056506 | 38580917 | *CARD10* | T | G | 0.6 | 2.46E-13 | ----- | 0 | 0.74 | 0.78 | 6.19E-01 | +- | 0 |
| 22 | rs6519240 | 41416557 | *CACNA1I* | T | C | 0.24 | 1.83E-27 | ----- | 0 | 0.27 | 0.24 | 8.87E-01 | +- | 0 |
| 22 | rs73175083 | 46383612 | *WNT7B* | T | C | 0.32 | 1.42E-26 | -?--- | 71.3 | NA | NA | NA | NA | NA |

[a]The results wre from Hysi PG, et al. Meta-analysis of 542,934 subjects of European ancestry identifies new genes and mechanisms predisposing to refractive error and myopia. Nat Genet. 2020 Apr;52(4):401-407. doi: 10.1038/s41588-020-0599-0. Epub 2020 Mar 30. $I^2$ (%), Heterogeneity. EA, effect allele; NEA, non-effect allele; EAF, effect allele frequency.

**Table S12. Frequency distribution in our study and the 1000Genome Project**

| SNP | Gene | EA | Frequency of EA in our study | | | | | | | Frequency of EA in 1000 Genome Project | | | | | |
|---|---|---|---|---|---|---|---|---|---|---|---|---|---|---|---|
| | | | HKCES-1 (N=864) | LAMP (N=373) | HKCES-2 (N=2066) | SMS (N=2027) | HKCES_all (N=2930) | Adult-1 (N=1212) | Adult-2 (N=602) | Global (N=2504) | AFR (N=661) | AMR (N=347) | EAS (N=504) | EUR (N=503) | SAS (N=489) |
| rs292034 | *MIR4275* | G | 0.013 | 0.011 | 0.011 | 0.013 | 0.012 | 0.017 | 0.017 | 0.15 | 0.19 | 0.15 | 0.01 | 0.27 | 0.11 |
| rs17074027 | *TENM3* | G | 0.058 | 0.06 | 0.076 | 0.073 | 0.071 | 0.077 | 0.068 | 0.09 | 0.08 | 0.08 | 0.09 | 0.06 | 0.15 |
| rs6925312 | *LOC101928911* | G | 0.78 | 0.76 | 0.78 | 0.790 | 0.780 | 0.79 | 0.77 | 0.47 | 0.26 | 0.51 | 0.76 | 0.42 | 0.47 |
| rs4609227 | *FAM135B* | C | 0.025 | 0.025 | 0.03 | 0.037 | 0.025 | 0.028 | 0.026 | 0.10 | 0.08 | 0.07 | 0.05 | 0.11 | 0.14 |

EA, effect allele; AFR, Africa; AMR, America; EAS, East Asia; EUR, Europe; SAS, South Asia.

**Table S13. Interactions between genetic risk score strata of spherical equivalent and diopter-hours and outdoor time**

| | Marginal effect model[a] | | | Ineraction effect model[b] | | | Ineraction effect model[c] | | |
|---|---|---|---|---|---|---|---|---|---|
| | **β** | **Std.Error** | **P** | **β** | **Std.Error** | **P** | **β** | **Std.Error** | **P** |
| GRS | 1.02 | 0.13 | **$2.14 \times 10^{-15}$** | - | - | - | - | - | - |
| Diopter-hours | -0.032 | 0.0078 | **$3.49 \times 10^{-5}$** | - | - | - | - | - | - |
| Outdoor time | 0.23 | 0.048 | **$1.76 \times 10^{-6}$** | - | - | - | - | - | - |
| GRS:Diopter-hours | - | - | - | 0.14 | 0.038 | **0.0003** | - | - | - |
| GRS:Outdoor time | - | - | - | - | - | - | -0.0031 | 0.2 | 0.99 |

[a] The marginal model was adjusted for age and sex.

[b] In the interaction model, multiplicative interaction effect was estimated as the product of the individual effects of GRS and diopter-hours with age, sex and outdoor time adjusted.

[c] In the interaction model, multiplicative interaction effect was estimated as the product of the individual effects of GRS and outdoor time with age, sex and diopter-hours adjusted.

Toal of 2057 children in HKCES-2 with data of diopter-hours and outdoor time are used for analysis in each model.

**Table S14. Associations of strata of genetic risk score and diopter-hours in relation to myopia status**

| Strata of diopter-hours | GRS strata | Control (N=1628) | Case (N=429) | OR (95%CI) | P |
|---|---|---|---|---|---|
| Q1 (0.4 to < 7.0 diopter-hours/day) | Low | 148 | 23 | 1 | - |
| | Moderate | 196 | 34 | 1.1 (0.62, 1.98) | 0.74 |
| | High | 79 | 12 | 0.95 (0.43, 2) | 0.90 |
| P for trend | 0.95 | | | | |
| Q2 (7.0 to < 9.0 diopter-hours/day) | Low | 135 | 19 | 0.91 (0.47, 1.74) | 0.77 |
| | Moderate | 158 | 31 | 1.07 (0.60, 1.94) | 0.83 |
| | High | 94 | 25 | 1.54 (0.82, 2.91) | 0.18 |
| P for trend | 0.07 | | | | |
| Q3 (9.0 to < 12.0 diopter-hours/day) | Low | 136 | 23 | 1.03 (0.55, 1.94) | 0.91 |
| | Moderate | 177 | 47 | 1.6 (0.93, 2.82) | 0.10 |
| | High | 70 | 32 | 2.81 (1.64, 5.6) | **0.00044** |
| P for trend | **0.00039** | | | | |
| Q4 ( ≥ 12.0 diopter-hours/day) | Low | 155 | 47 | 1.78 (1.03, 3.14) | **0.04** |
| | Moderate | 31 | 12 | 1.95 (0.77, 4.96) | 0.15 |
| | High | 249 | 124 | 3.01 (1.73, 4.71) | **4.70E-05** |
| P for trend | **0.011** | | | | |

Multivariable model with futher adjustment for age, sex and outdoor time.

Myopia is defined as ≤ -0.5D.

Toal of 2057 children in HKCES-2 with data of diopter-hours and outdoor time are used for analysis.

**Table S15. Functional prediciton of 4 genome-wide significance SNPs**

| | | Roadmap Epigenomics Project | | | Encyclopedia of DNA Elements Project | | |
|---|---|---|---|---|---|---|---|
| **SNP** | **CHR** | **Feature Type Class** | **Feature Type** | **Epigenome** | **Feature Type Class** | **Feature Type** | **Epigenome** |
| rs292034 | 4 | Histone | H3K9me3 | Placenta | Histone | H3K9me3 | HepG2 |
| | | | H3K14ac | IMR90 | | | |
| rs17074027 | 4 | Histone | H3K36me3 | Neural stem progenitor cell, H1 hESC, HUES64 | Histone | H3K36me3 | Bipolar neuron, SK N SH |
| | | | H3K36me3, H3K4me1 | Neuron | | | |
| | | | H3K27me3 | T cells | | H3K9me3 | Bipolar neuron |
| | | | H3K79me1 | IMR90 | | | |
| | | | H3K4me1 | H1 hESC, HUES64, iPS 20b, HUES48 | | H3K27me3 | PC9, A549 |
| | | | H2BK20ac | H9 | | | |
| rs6925312 | 6 | Histone | H3K4me1 | Neutrophil, Monocytes-CD14+ (PB), HUES64, iPS 20b, HUES48, neuron, iPS 15b, neural stem progenitor cell, HUES6, endodermal cell, H1 hESC | Histone | H3K4me1 | Bipolar neuron, monocytes-CD14+, GM12878, Karpas 42, MM 1S |
| | | | H3K27ac | Spleen | | | |
| | | | H3K4me3 | CD4 positive CD25 positive alpha beta regulatory T cell | | H3K36me3 | Bipolar neuron |
| | | | H3K27me3 | IMR90 | | H3K27me3 | HSMMtube, myotube, NH-A, DND-41, astrocyte, skeletal muscle myoblast, HSMM |
| rs4609227 | 8 | Histone | H3K9me3 | Endodermal cell, HUES64, HUES48, HUES6, fetal muscle Leg, iPS 20b, H1 hESC, IMR 90, monocytes-CD14+, placenta, kidney | Histone | H3K9me3 | Bipolar neuron, HepG2, HCT116, Hela-S3 |
| | | | | | | H3K4me1 | PC3, Hela-S3 |
| | | | | | Open Chromatin | DNase1 | PC3 |
| | | | H2BK12ac, H3K14ac, H2AK5ac | H1-trophoblast | Transcription Factor | JunD | Hela-S3 |

IMR90: human lung fibroblast cell line IMR90; H1 hESC: H1 human embryonic stem cells; HUES: homo sapiens embryonic stem cell; iPS: induced pluripotent stem cell; SK N SH: human neuroblastoma cell line; PC9: human non-small cell lung cancer (NSCLC) cell line; A549: adenocarcinomic human alveolar basal epithelial cell line; GM12878: lymphoblastoid cell line; MM 1S: multiple myeloma cell line; HSMM: skeletal muscle myoblasts; NH-A: normal human astrocytes; DND-41: T cell leukemia cell line; HCT116: human colon cancer cell line; Hela-S3: human cervical epithlioid carcinoma cell line; PC3: human prostate cancer cell line; DNase1: deoxyribonuclease I.
Annotations are avialible at SNPnexus https://www.snp-nexus.org/v4/.

**Table S16. Gene Ontology Terms Significantly Enriched among Candidate Genes**

| Gene Ontology Identification | Gene Ontology Term | Category, Level | Gene Set Size | Candidate Genes Contained[a] | P Value | q Value |
|---|---|---|---|---|---|---|
| GO:0007156 | Homophilic cell adhesion via plasma membrane adhesion molecules | BP, 5 | 169 | *TENM3; PCDH7* | 0.00073 | 0.00073 |
| GO:0098742 | Cell-cell adhesion via plasma-membrane adhesion molecules | BP, 4 | 275 | *TENM3; PCDH7* | 0.0019 | 0.015 |
| GO:0030667 | Secretory granule membrane | CC, 3 | 306 | *SPACA1; PCDH7* | 0.0024 | 0.024 |
| GO:0042802 | Identical protein binding | MF, 3 | 1948 | *TENM3; DCTD; AKIRIN2* | 0.0085 | 0.017 |

GO, Gene ontology; BP,Biological process; CC, Cellular components; MF, Molecular founction.

[a] The nearby coding gene *PCDH7* (1.9Mpb downstream of *MIR4275* ), *AKIRIN2* (82.6Kbp upstream of *LOC101928911* ) and *SPACA1* (136 Kbp downstream of *LOC101928911* ) and *DCTD* (87 Kbp downstream of *TENM3* ) were additionally included in the GO analysis.

**Table S17. Credible set of SNPs identified using CAVIAR**

| CHR | BP | SNP | A1 | A2 | Prob_in_pCausalSet | Causal_Post_Prob | r2 |
|---|---|---|---|---|---|---|---|
| **4** | **28478119** | **rs292034** | **G** | **T** | **0.050** | **0.097** | **1.00** |
| 4 | 28482974 | rs55684803 | T | A | 0.006 | 0.011 | 1.00 |
| 4 | 28484234 | rs569100 | C | T | 0.006 | 0.011 | 1.00 |
| 4 | 28484389 | rs567993 | G | T | 0.006 | 0.011 | 1.00 |
| 4 | 28488065 | rs292085 | A | G | 0.006 | 0.011 | 1.00 |
| 4 | 28489008 | rs292084 | C | T | 0.006 | 0.011 | 1.00 |
| 4 | 28489163 | rs1402670715 | A | T | 0.005 | 0.009 | 0.97 |
| 4 | 28490381 | rs292082 | C | T | 0.006 | 0.011 | 1.00 |
| 4 | 28493198 | rs292079 | G | A | 0.006 | 0.011 | 1.00 |
| 4 | 28493840 | rs292078 | C | G | 0.006 | 0.011 | 1.00 |
| 4 | 28494313 | rs292076 | A | G | 0.006 | 0.011 | 1.00 |
| 4 | 28496256 | rs292072 | A | G | 0.004 | 0.008 | 0.97 |
| 4 | 28497583 | rs292065 | T | C | 0.004 | 0.008 | 0.97 |
| 4 | 28498910 | rs292063 | G | A | 0.004 | 0.008 | 0.97 |
| 4 | 28499782 | rs292062 | G | A | 0.004 | 0.008 | 0.97 |
| 4 | 28500681 | rs292060 | A | C | 0.004 | 0.008 | 0.97 |
| 4 | 28503151 | rs292057 | T | C | 0.004 | 0.008 | 0.97 |
| 4 | 28506937 | rs28645352 | T | G | 0.004 | 0.008 | 0.97 |
| 4 | 28507244 | rs1303819 | G | A | 0.004 | 0.008 | 0.97 |
| 4 | 28507335 | rs1303818 | G | A | 0.004 | 0.008 | 0.97 |
| 4 | 28507573 | rs1303817 | T | C | 0.004 | 0.008 | 0.97 |
| 4 | 28507739 | rs1303816 | A | G | 0.004 | 0.008 | 0.94 |
| 4 | 28509079 | rs13147296 | A | C | 0.004 | 0.008 | 0.97 |
| 4 | 28518533 | rs13123915 | G | C | 0.004 | 0.008 | 0.97 |
| 4 | 28519470 | rs673272 | G | A | 0.004 | 0.008 | 0.97 |
| 4 | 28520773 | rs893217957 | C | A | 0.003 | 0.007 | 0.94 |
| 4 | 28521854 | rs292053 | G | T | 0.004 | 0.008 | 0.97 |
| 4 | 28522519 | rs292052 | C | T | 0.004 | 0.008 | 0.97 |
| 4 | 28524559 | rs611376 | T | C | 0.004 | 0.008 | 0.97 |
| 4 | 28525051 | rs292049 | T | C | 0.001 | 0.002 | 0.88 |

**Table S17. Credible set of SNPs identified using CAVIAR**

| CHR | BP | SNP | A1 | A2 | Prob_in_pCausalSet | Causal_Post_Prob | r2 |
|---|---|---|---|---|---|---|---|
| 4 | 28527304 | rs167106 | C | A | 0.004 | 0.008 | 0.97 |
| 4 | 28527537 | rs292047 | T | G | 0.006 | 0.012 | 0.84 |
| 4 | 28528060 | rs10517147 | C | T | 0.004 | 0.008 | 0.97 |
| 4 | 28532565 | rs638223 | C | T | 0.004 | 0.008 | 0.97 |
| 4 | 28538250 | rs292038 | G | C | 0.004 | 0.008 | 0.97 |
| 4 | 28538736 | rs1510712 | G | A | 0.004 | 0.008 | 0.97 |
| 4 | 28539924 | rs687535 | A | G | 0.001 | 0.002 | 0.88 |
| 4 | 28540930 | rs13125329 | C | A | 0.004 | 0.008 | 0.97 |
| 4 | 28542348 | rs542264 | G | C | 0.004 | 0.008 | 0.97 |
| 4 | 28542928 | rs292014 | A | G | 0.004 | 0.008 | 0.97 |
| 4 | 28545048 | rs167104 | G | A | 0.002 | 0.004 | 0.94 |
| 4 | 28547108 | rs557255 | T | C | 0.002 | 0.004 | 0.94 |
| 4 | 28557165 | rs2458606 | G | A | 0.006 | 0.011 | 0.83 |
| 4 | 28558043 | rs2458607 | G | A | 0.006 | 0.011 | 0.83 |
| 4 | 28560540 | rs2458608 | A | G | 0.006 | 0.011 | 0.83 |
| 4 | 28561303 | rs2995111 | A | G | 0.006 | 0.011 | 0.83 |
| 4 | 28561374 | rs2960592 | T | C | 0.006 | 0.011 | 0.83 |
| 4 | 28562034 | rs2471721 | C | T | 0.006 | 0.011 | 0.83 |
| 4 | 28563166 | rs2471720 | A | G | 0.006 | 0.011 | 0.83 |
| 4 | 28563313 | rs2458613 | A | G | 0.006 | 0.011 | 0.83 |
| 4 | 28563413 | rs2471719 | C | T | 0.006 | 0.011 | 0.83 |
| 4 | 28563455 | rs2458614 | G | A | 0.006 | 0.011 | 0.83 |
| 4 | 28565123 | rs2458616 | A | C | 0.006 | 0.011 | 0.83 |
| 4 | 28565233 | rs2471718 | G | A | 0.006 | 0.011 | 0.83 |
| 4 | 28566571 | rs2458617 | C | T | 0.006 | 0.011 | 0.83 |
| 4 | 28566686 | rs2458618 | T | G | 0.006 | 0.011 | 0.83 |
| 4 | 28568637 | rs2943155 | C | T | 0.006 | 0.011 | 0.83 |
| 4 | 28568717 | rs2943156 | C | G | 0.006 | 0.011 | 0.83 |
| 4 | 28569819 | rs2471717 | T | G | 0.006 | 0.011 | 0.83 |
| 4 | 28570278 | rs2471715 | T | G | 0.006 | 0.011 | 0.83 |

**Table S17. Credible set of SNPs identified using CAVIAR**

| CHR | BP | SNP | A1 | A2 | Prob_in_pCausalSet | Causal_Post_Prob | r2 |
|---|---|---|---|---|---|---|---|
| 4 | 28571563 | rs12505899 | C | T | 0.006 | 0.012 | 0.65 |
| 4 | 28571606 | rs12501893 | T | C | 0.006 | 0.012 | 0.65 |
| 4 | 28571640 | rs2943157 | T | C | 0.006 | 0.012 | 0.65 |
| 4 | 28571652 | rs2943158 | G | T | 0.006 | 0.012 | 0.65 |
| 4 | 28571988 | rs1514126 | G | A | 0.006 | 0.012 | 0.65 |
| 4 | 28572095 | rs1514128 | C | T | 0.006 | 0.012 | 0.65 |
| 4 | 28572856 | rs2458624 | A | G | 0.006 | 0.012 | 0.65 |
| 4 | 28575113 | rs2471710 | T | C | 0.001 | 0.002 | 0.57 |
| 4 | 28575292 | rs2471709 | T | C | 0.001 | 0.002 | 0.57 |
| 4 | 28575709 | rs2458626 | C | A | 0.001 | 0.002 | 0.57 |
| 4 | 28576379 | rs2458627 | C | A | 0.001 | 0.002 | 0.57 |
| 4 | 28577504 | rs1514129 | G | C | 0.005 | 0.01 | 0.64 |
| 4 | 28579020 | rs2458628 | T | C | 0.001 | 0.002 | 0.57 |
| 4 | 28580184 | rs2458629 | G | A | 0.001 | 0.002 | 0.57 |
| 4 | 28581335 | rs2471707 | A | C | 0.001 | 0.002 | 0.57 |
| 4 | 28581431 | rs2458630 | A | T | 0.005 | 0.01 | 0.64 |
| 4 | 28583060 | rs2471705 | A | G | 0.005 | 0.01 | 0.64 |
| 4 | 28584417 | rs2471703 | A | G | 0.005 | 0.01 | 0.64 |
| 4 | 28587846 | rs1168693995 | G | C | 0.005 | 0.01 | 0.64 |
| 4 | 28589079 | rs2471711 | T | C | 0.005 | 0.01 | 0.64 |
| 4 | 28589619 | rs2248575 | A | G | 0.005 | 0.01 | 0.64 |
| 4 | 28591924 | rs2458619 | A | G | 0.005 | 0.01 | 0.64 |
| 4 | 28593164 | rs2471685 | C | T | 0.005 | 0.01 | 0.64 |
| 4 | 28593978 | rs2471687 | C | T | 0.005 | 0.01 | 0.64 |
| 4 | 28594111 | rs2471688 | A | G | 0.005 | 0.01 | 0.64 |
| 4 | 28595064 | rs2471689 | G | A | 0.005 | 0.01 | 0.64 |
| 4 | 28596316 | rs2471690 | C | T | 0.005 | 0.01 | 0.64 |
| 4 | 28597991 | rs2471691 | C | T | 0.005 | 0.01 | 0.64 |
| 4 | 28598358 | rs2471692 | C | T | 0.005 | 0.01 | 0.64 |
| 4 | 28600877 | rs2458621 | C | G | 0.004 | 0.008 | 0.62 |

**Table S17. Credible set of SNPs identified using CAVIAR**

| CHR | BP | SNP | A1 | A2 | Prob_in_pCausalSet | Causal_Post_Prob | r2 |
|---|---|---|---|---|---|---|---|
| 4 | 28604977 | rs9790405 | C | T | 0.0002 | 0.0003 | 0.24 |
| 4 | 28678144 | rs1267296960 | T | A | 0.002 | 0.004 | 0.61 |
| 4 | 28719509 | rs1493159 | C | T | 0.003 | 0.005 | 0.58 |
| 4 | 28727427 | rs2087875 | A | G | 0.002 | 0.003 | 0.54 |
| 4 | 28735828 | rs28793586 | T | C | 0.002 | 0.003 | 0.54 |
| 4 | 28736038 | rs55869607 | A | G | 0.001 | 0.002 | 0.50 |
| 4 | 28736971 | rs28704662 | A | T | 0.001 | 0.002 | 0.50 |
| 4 | 28737112 | rs67524851 | C | T | 0.001 | 0.002 | 0.50 |
| 4 | 28738088 | rs10001415 | C | T | 0.001 | 0.002 | 0.50 |
| 4 | 28740012 | rs36071950 | A | C | 0.001 | 0.002 | 0.50 |
| 4 | 28741919 | rs1491311167 | T | A | 0.001 | 0.002 | 0.50 |
| 4 | 28745163 | rs77075089 | A | G | 0.001 | 0.002 | 0.50 |
| 4 | 28747310 | rs10001616 | A | G | 0.001 | 0.002 | 0.50 |
| 4 | 28748805 | rs71596161 | A | G | 0.001 | 0.002 | 0.50 |
| 4 | 28748810 | rs28401471 | T | C | 0.001 | 0.002 | 0.50 |
| 4 | 28748871 | rs28362049 | C | T | 0.001 | 0.002 | 0.50 |
| 4 | 28749863 | rs7695442 | T | A | 0.001 | 0.002 | 0.50 |
| 4 | 28750665 | rs13102811 | T | C | 0.001 | 0.002 | 0.50 |
| 4 | 28754779 | rs7693339 | T | A | 0.001 | 0.002 | 0.50 |
| 4 | 28761781 | rs6851565 | A | G | 0.002 | 0.004 | 0.51 |
| 4 | 28762951 | rs34024932 | T | C | 0.002 | 0.004 | 0.51 |
| 4 | 28767776 | rs11737432 | A | G | 0.002 | 0.004 | 0.51 |
| 4 | 28771767 | rs73215498 | A | T | 0.002 | 0.004 | 0.51 |
| 4 | 28774747 | rs73219413 | A | G | 0.002 | 0.003 | 0.50 |
| 4 | 28775853 | rs73219415 | G | A | 0.002 | 0.003 | 0.50 |
| 4 | 28779671 | rs66528608 | A | G | 0.002 | 0.003 | 0.50 |
| 4 | 28781817 | rs73219418 | T | A | 0.002 | 0.003 | 0.50 |
| 4 | 28788513 | rs13144199 | A | T | 0.002 | 0.003 | 0.50 |
| 4 | 28791121 | rs13113800 | C | T | 0.002 | 0.003 | 0.50 |
| 4 | 28792788 | rs1353049 | C | T | 0.002 | 0.003 | 0.50 |

**Table S17. Credible set of SNPs identified using CAVIAR**

| CHR | BP | SNP | A1 | A2 | Prob_in_pCausalSet | Causal_Post_Prob | r2 |
|---|---|---|---|---|---|---|---|
| 4 | 28793330 | rs35538171 | A | G | 0.002 | 0.003 | 0.50 |
| 4 | 28795187 | rs67741650 | T | C | 0.002 | 0.003 | 0.50 |
| 4 | 28798954 | rs67753751 | C | A | 0.002 | 0.003 | 0.50 |
| 4 | 28799742 | rs13140136 | C | T | 0.002 | 0.003 | 0.50 |
| 4 | 28801221 | rs34072092 | C | T | 0.002 | 0.003 | 0.50 |
| 4 | 28812966 | rs34575420 | A | T | 0.001 | 0.002 | 0.53 |
| 4 | 28813116 | rs34231546 | A | C | 0.001 | 0.002 | 0.53 |
| 4 | 28813302 | rs13118585 | T | C | 0.001 | 0.002 | 0.53 |
| 4 | 28816220 | rs7349599 | A | G | 0.001 | 0.002 | 0.53 |
| 4 | 28820334 | rs35351681 | T | C | 0.001 | 0.002 | 0.53 |
| 4 | 28827186 | rs35168262 | T | C | 0.001 | 0.002 | 0.53 |
| 4 | 28827190 | rs2309887 | T | G | 0.0004 | 0.001 | 0.47 |
| 4 | 28829722 | rs71596163 | A | G | 0.001 | 0.002 | 0.53 |
| 4 | 28831118 | rs67012720 | C | G | 0.001 | 0.002 | 0.53 |
| 4 | 28832740 | rs17653404 | G | A | 0.001 | 0.002 | 0.53 |
| 4 | 28836491 | rs541307772 | C | T | 0.0002 | 0.0004 | 0.47 |
| 4 | 28838463 | rs13125283 | C | T | 0.0002 | 0.0004 | 0.47 |
| 4 | 28841728 | rs36008028 | A | G | 0.0002 | 0.0004 | 0.47 |
| 4 | 28842321 | rs13127063 | A | G | 0.0002 | 0.0004 | 0.47 |
| 4 | 28848518 | rs11730630 | C | T | 0.0002 | 0.0004 | 0.47 |
| 4 | 28851739 | rs34940643 | G | A | 0.0002 | 0.0004 | 0.47 |
| 4 | 28853720 | rs75924892 | A | G | 0.0002 | 0.0004 | 0.47 |
| 4 | 28854021 | rs111923755 | T | C | 0.0003 | 0.001 | 0.42 |
| 4 | 28854081 | rs35875568 | C | T | 0.0003 | 0.001 | 0.42 |
| 4 | 28854365 | rs13124043 | G | A | 0.0003 | 0.001 | 0.42 |
| 4 | 28855098 | rs4450915 | C | T | 0.0003 | 0.001 | 0.42 |
| 4 | 28855370 | rs10021028 | C | T | 0.0003 | 0.001 | 0.42 |
| 4 | 28855708 | rs10011877 | C | G | 0.0003 | 0.001 | 0.42 |
| 4 | 28855799 | rs145797021 | T | G | 0.0003 | 0.001 | 0.42 |
| 4 | 28856428 | rs4467552 | A | G | 0.0003 | 0.001 | 0.42 |

**Table S17. Credible set of SNPs identified using CAVIAR**

| CHR | BP | SNP | A1 | A2 | Prob_in_pCausalSet | Causal_Post_Prob | r2 |
|---|---|---|---|---|---|---|---|
| 4 | 28856499 | rs4461513 | C | A | 0.0003 | 0.001 | 0.42 |
| 4 | 28856694 | rs4459975 | A | G | 0.0003 | 0.001 | 0.42 |
| 4 | 28856766 | rs4459978 | C | T | 0.0003 | 0.001 | 0.42 |
| 4 | 28856826 | rs146120146 | T | A | 0.0003 | 0.001 | 0.42 |
| 4 | 28856930 | rs141149319 | G | A | 0.0003 | 0.001 | 0.42 |
| 4 | 28858062 | rs28876111 | G | A | 0.0002 | 0.0005 | 0.40 |
| 4 | 28858325 | rs28857240 | A | G | 0.0002 | 0.0005 | 0.38 |
| 4 | 28858542 | rs28798375 | A | C | 0.0002 | 0.0005 | 0.40 |
| 4 | 28858558 | rs28876079 | T | C | 0.0002 | 0.0005 | 0.40 |
| 4 | 28858575 | rs1202616961 | A | G | 0.0002 | 0.0005 | 0.40 |
| 4 | 28858578 | rs28868298 | A | G | 0.0002 | 0.0005 | 0.40 |
| 4 | 28858857 | rs6828321 | G | A | 0.0002 | 0.0005 | 0.40 |
| 4 | 28858875 | rs6828839 | A | G | 0.0002 | 0.0005 | 0.40 |
| 4 | 28858917 | rs13120881 | T | C | 0.0002 | 0.0004 | 0.44 |
| 4 | 28859001 | rs6828537 | G | A | 0.0002 | 0.0005 | 0.40 |
| 4 | 28859077 | rs6829283 | A | G | 0.0002 | 0.0005 | 0.40 |
| 4 | 28859332 | rs73221285 | G | A | 0.0002 | 0.0005 | 0.40 |
| 4 | 28859532 | rs28419219 | A | G | 0.0002 | 0.0005 | 0.40 |
| 4 | 28859561 | rs28508131 | G | A | 0.0002 | 0.0005 | 0.40 |
| 4 | 28859652 | rs28674090 | G | A | 0.0002 | 0.0005 | 0.40 |
| 4 | 28859923 | rs28710451 | C | G | 0.0002 | 0.0005 | 0.40 |
| 4 | 28859955 | rs28532690 | G | A | 0.0002 | 0.0005 | 0.40 |
| 4 | 28860189 | rs13129333 | C | G | 0.0002 | 0.0005 | 0.40 |
| 4 | 28860282 | rs13129541 | C | G | 0.0002 | 0.0004 | 0.44 |
| 4 | 28860291 | rs10025076 | T | G | 0.0002 | 0.0005 | 0.40 |
| 4 | 28860599 | rs9992199 | A | T | 0.0002 | 0.0005 | 0.40 |
| 4 | 28861789 | rs11736200 | T | C | 0.0002 | 0.0005 | 0.40 |
| 4 | 28861911 | rs10008384 | G | A | 0.0002 | 0.0005 | 0.40 |
| 4 | 28862413 | rs10031220 | T | C | 0.0002 | 0.0005 | 0.40 |
| 4 | 28862538 | rs4280718 | G | A | 0.0002 | 0.0005 | 0.40 |

**Table S17. Credible set of SNPs identified using CAVIAR**

| CHR | BP | SNP | A1 | A2 | Prob_in_pCausalSet | Causal_Post_Prob | r2 |
|---|---|---|---|---|---|---|---|
| 4 | 28862780 | rs73221298 | T | C | 0.0002 | 0.0004 | 0.44 |
| 4 | 28863159 | rs7698028 | G | A | 0.0002 | 0.0005 | 0.40 |
| 4 | 28863746 | rs7682215 | C | T | 0.0002 | 0.0005 | 0.40 |
| 4 | 28863905 | rs12331292 | A | T | 0.0002 | 0.0005 | 0.40 |
| 4 | 28863907 | rs12330987 | A | C | 0.0002 | 0.0005 | 0.40 |
| 4 | 28864153 | rs7439579 | C | T | 0.0002 | 0.0005 | 0.40 |
| 4 | 28864709 | rs3924142 | T | C | 0.0002 | 0.0005 | 0.40 |
| 4 | 28865223 | rs34939094 | A | G | 0.0002 | 0.0005 | 0.40 |
| 4 | 28865703 | rs28544086 | T | C | 0.0002 | 0.0005 | 0.40 |
| 4 | 28867318 | rs67583658 | A | C | 0.0002 | 0.0003 | 0.37 |
| 4 | 28867795 | rs6853736 | G | A | 0.0002 | 0.0003 | 0.37 |
| 4 | 28868305 | rs10033285 | T | C | 0.0002 | 0.0003 | 0.37 |
| 4 | 28868349 | rs7356502 | G | A | 0.0002 | 0.0003 | 0.37 |
| 4 | 28868389 | rs10000627 | A | T | 0.0002 | 0.0003 | 0.37 |
| 4 | 28868549 | rs979446864 | G | T | 0.0002 | 0.0003 | 0.37 |
| 4 | 28868688 | rs34723504 | T | C | 0.0002 | 0.0003 | 0.37 |
| 4 | 28869631 | rs35412443 | A | G | 0.0002 | 0.0003 | 0.37 |
| 4 | 28871596 | rs13146956 | A | T | 0.0002 | 0.0004 | 0.38 |
| 4 | 28871862 | rs28633494 | G | A | 0.0002 | 0.0004 | 0.38 |
| 4 | 28873007 | rs73222920 | G | A | 0.0002 | 0.0004 | 0.38 |
| 4 | rs118081337 | 183654119 | A | G | 0.0018 | 0.0032 | 0.31 |
| 4 | rs118081337 | 183654119 | A | G | 0.002 | 0.003 | 0.31 |
| 4 | rs11938233 | 183605088 | A | G | 0.00052 | 0.00094 | 0.16 |
| 4 | rs13435237 | 183654099 | G | A | 0.0011 | 0.0021 | 0.26 |
| 4 | rs13435237 | 183654099 | G | A | 0.001 | 0.002 | 0.26 |
| **4** | **rs17074027** | **183678700** | **G** | **T** | **0.22** | **0.4** | **1.00** |
| 4 | rs17074036 | 183685402 | A | G | 0.003 | 0.005 | 0.55 |
| 4 | rs17074036 | 183685402 | A | G | 0.0027 | 0.0048 | 0.55 |
| 4 | rs4862091 | 183684204 | C | T | 0.006 | 0.01 | 0.37 |
| 4 | rs4862091 | 183684204 | C | T | 0.0055 | 0.01 | 0.37 |

**Table S17. Credible set of SNPs identified using CAVIAR**

| CHR | BP | SNP | A1 | A2 | Prob_in_pCausalSet | Causal_Post_Prob | r2 |
|---|---|---|---|---|---|---|---|
| 4 | rs6552604 | 183663607 | T | C | 0.0012 | 0.0022 | 0.29 |
| 4 | rs6552604 | 183663607 | T | C | 0.001 | 0.002 | 0.29 |
| 4 | rs6829132 | 183681725 | C | A | 0.006 | 0.01 | 0.59 |
| 4 | rs6829132 | 183681725 | C | A | 0.0055 | 0.01 | 0.59 |
| 4 | rs7691449 | 183660235 | G | A | 0.001 | 0.003 | 0.27 |
| 4 | rs7691449 | 183660235 | G | A | 0.0014 | 0.0025 | 0.27 |
| 4 | rs9993361 | 183671543 | G | A | 0.0012 | 0.0022 | 0.31 |
| 4 | rs9993361 | 183671543 | G | A | 0.001 | 0.002 | 0.31 |
| 6 | 88546326 | rs2499497 | T | C | 0.0003 | 0.001 | 0.22 |
| 6 | 88547515 | rs2787913 | A | G | 0.0003 | 0.001 | 0.21 |
| 6 | 88549795 | rs718220 | G | A | 0.0003 | 0.001 | 0.23 |
| 6 | 88550425 | rs2499495 | T | G | 0.0003 | 0.001 | 0.23 |
| 6 | 88550917 | rs2477310 | C | T | 0.0003 | 0.001 | 0.23 |
| 6 | 88555067 | rs1324688 | T | C | 0.001 | 0.002 | 0.45 |
| 6 | 88556885 | rs1324689 | T | C | 0.001 | 0.002 | 0.48 |
| 6 | 88559273 | rs9344734 | T | G | 0.001 | 0.001 | 0.47 |
| 6 | 88559472 | rs9344735 | A | G | 0.001 | 0.002 | 0.48 |
| 6 | 88559495 | rs9344736 | A | G | 0.001 | 0.002 | 0.48 |
| 6 | 88560207 | rs9342126 | A | G | 0.001 | 0.001 | 0.47 |
| 6 | 88562207 | rs373513473 | A | G | 0.001 | 0.002 | 0.48 |
| 6 | 88562282 | 6:88562282:TAA | T | TAAC | 0.001 | 0.001 | 0.48 |
| 6 | 88562327 | rs574644780 | CT | C | 0.001 | 0.001 | 0.43 |
| 6 | 88563032 | rs6454654 | T | C | 0.001 | 0.002 | 0.48 |
| 6 | 88563240 | rs1222677393 | A | AT | 0.001 | 0.001 | 0.46 |
| 6 | 88563303 | rs6415023 | C | G | 0.001 | 0.002 | 0.48 |
| 6 | 88563547 | rs6454655 | G | A | 0.001 | 0.002 | 0.48 |
| 6 | 88564756 | rs9353509 | T | C | 0.001 | 0.002 | 0.48 |
| 6 | 88566365 | rs9351134 | A | T | 0.001 | 0.002 | 0.48 |
| 6 | 88568988 | rs56071491 | T | C | 0.001 | 0.002 | 0.49 |
| 6 | 88569002 | rs55764175 | G | A | 0.001 | 0.002 | 0.49 |

**Table S17. Credible set of SNPs identified using CAVIAR**

| CHR | BP | SNP | A1 | A2 | Prob_in_pCausalSet | Causal_Post_Prob | r2 |
|---|---|---|---|---|---|---|---|
| 6 | 88569098 | rs6939791 | A | G | 0.001 | 0.002 | 0.49 |
| 6 | 88569317 | 6:88569317:CT:C | C | CT | 0.001 | 0.002 | 0.49 |
| 6 | 88569662 | rs6940862 | A | G | 0.001 | 0.002 | 0.49 |
| 6 | 88569807 | rs73752066 | A | G | 0.001 | 0.002 | 0.48 |
| 6 | 88570008 | rs7770681 | G | T | 0.001 | 0.002 | 0.48 |
| 6 | 88571034 | rs4615355 | T | G | 0.001 | 0.002 | 0.52 |
| 6 | 88571289 | rs77565838 | G | C | 0.001 | 0.002 | 0.48 |
| 6 | 88571309 | rs6454657 | A | G | 0.001 | 0.002 | 0.52 |
| 6 | 88571437 | rs6454658 | A | G | 0.001 | 0.002 | 0.52 |
| 6 | 88571803 | rs1327051952 | G | C | 0.003 | 0.006 | 0.55 |
| 6 | 88571968 | rs9450817 | A | G | 0.002 | 0.004 | 0.55 |
| 6 | 88575196 | rs4298315 | G | C | 0.002 | 0.004 | 0.55 |
| 6 | 88575620 | rs9362457 | G | T | 0.002 | 0.004 | 0.55 |
| 6 | 88575716 | rs9344737 | T | C | 0.002 | 0.003 | 0.47 |
| 6 | 88577883 | rs3923216 | A | G | 0.008 | 0.016 | 0.66 |
| 6 | 88578195 | rs6415024 | T | C | 0.014 | 0.026 | 0.70 |
| 6 | 88578373 | rs4320331 | T | C | 0.013 | 0.024 | 0.74 |
| 6 | 88578393 | rs4389736 | G | A | 0.013 | 0.024 | 0.74 |
| 6 | 88579382 | rs4537110 | G | A | 0.013 | 0.024 | 0.75 |
| 6 | 88579560 | rs4129674 | A | G | 0.013 | 0.024 | 0.74 |
| 6 | 88579705 | rs4129673 | C | G | 0.013 | 0.024 | 0.74 |
| 6 | 88580664 | rs4506018 | A | G | 0.013 | 0.025 | 0.74 |
| 6 | 88582415 | rs9294392 | T | A | 0.013 | 0.025 | 0.79 |
| 6 | 88582598 | rs9344738 | T | C | 0.005 | 0.009 | 0.70 |
| 6 | 88584710 | rs1464272840 | C | CAAA | 0.009 | 0.016 | 0.80 |
| 6 | 88585489 | rs9444561 | T | C | 0.008 | 0.015 | 0.73 |
| 6 | 88588004 | rs9784861 | A | G | 0.008 | 0.015 | 0.73 |
| 6 | 88592819 | rs4519980 | G | A | 0.016 | 0.03 | 0.83 |
| 6 | 88593579 | rs9359760 | T | C | 0.006 | 0.01 | 0.73 |
| 6 | 88596854 | rs4485983 | A | G | 0.030 | 0.056 | 0.85 |

**Table S17. Credible set of SNPs identified using CAVIAR**

| CHR | BP | SNP | A1 | A2 | Prob_in_pCausalSet | Causal_Post_Prob | r2 |
|---|---|---|---|---|---|---|---|
| 6 | 88603026 | rs9359761 | G | A | 0.049 | 0.09 | 0.91 |
| 6 | 88605297 | 6:88605297:TG:T | T | TG | 0.015 | 0.027 | 0.80 |
| 6 | 88606134 | rs9450821 | A | G | 0.076 | 0.14 | 0.91 |
| 6 | 88608343 | rs9450823 | G | C | 0.001 | 0.003 | 0.21 |
| 6 | 88609397 | rs9362458 | A | G | 0.007 | 0.013 | 0.86 |
| 6 | 88609933 | rs5878052 | C | CT | 0.001 | 0.003 | 0.21 |
| 6 | 88610137 | rs4235834 | T | G | 0.001 | 0.003 | 0.21 |
| 6 | 88611793 | rs6918951 | C | T | 0.001 | 0.003 | 0.21 |
| 6 | 88611850 | rs6903270 | T | C | 0.001 | 0.003 | 0.21 |
| **6** | **88612769** | **rs6925312** | **A** | **G** | **0.079** | **0.15** | **1.00** |
| 8 | 138068064 | rs36014845 | G | A | 0.0002 | 0.0005 | 0.20 |
| 8 | 138068934 | rs34063731 | C | T | 0.001 | 0.001 | 0.25 |
| 8 | 138069118 | rs35628260 | AC | A | 0.001 | 0.001 | 0.26 |
| 8 | 138069364 | rs11984900 | T | G | 0.001 | 0.001 | 0.25 |
| 8 | 138070221 | rs34266606 | C | T | 0.001 | 0.001 | 0.25 |
| 8 | 138070877 | rs11997096 | G | A | 0.001 | 0.001 | 0.25 |
| 8 | 138071307 | rs72736397 | T | C | 0.001 | 0.001 | 0.25 |
| 8 | 138071531 | rs78342455 | T | G | 0.001 | 0.001 | 0.25 |
| 8 | 138073544 | rs34164683 | G | A | 0.001 | 0.001 | 0.25 |
| 8 | 138073934 | rs13267873 | T | C | 0.001 | 0.001 | 0.25 |
| 8 | 138075582 | rs116713894 | A | G | 0.001 | 0.001 | 0.25 |
| 8 | 138077925 | rs111833581 | G | T | 0.001 | 0.001 | 0.25 |
| 8 | 138079129 | rs1012244813 | T | TA | 0.001 | 0.001 | 0.25 |
| 8 | 138079747 | rs34948159 | T | G | 0.001 | 0.001 | 0.25 |
| 8 | 138080449 | rs13260461 | A | G | 0.001 | 0.001 | 0.25 |
| 8 | 138081290 | rs4295697 | G | A | 0.001 | 0.001 | 0.25 |
| 8 | 138082238 | rs34065840 | T | G | 0.0005 | 0.001 | 0.26 |
| 8 | 138083152 | rs71532111 | T | G | 0.001 | 0.001 | 0.27 |
| 8 | 138083408 | rs71532112 | C | G | 0.0005 | 0.001 | 0.26 |
| 8 | 138085350 | rs71532113 | T | G | 0.0005 | 0.001 | 0.26 |

**Table S17. Credible set of SNPs identified using CAVIAR**

| CHR | BP | SNP | A1 | A2 | Prob_in_pCausalSet | Causal_Post_Prob | r2 |
|---|---|---|---|---|---|---|---|
| 8 | 138085500 | rs71532114 | A | T | 0.0005 | 0.001 | 0.26 |
| 8 | 138086085 | rs35498675 | GT | G | 0.0005 | 0.001 | 0.26 |
| 8 | 138087924 | rs76844363 | G | A | 0.0005 | 0.001 | 0.26 |
| 8 | 138088089 | rs35725671 | T | G | 0.0005 | 0.001 | 0.26 |
| 8 | 138088503 | rs36074855 | T | C | 0.0005 | 0.001 | 0.26 |
| 8 | 138090760 | rs35926600 | A | G | 0.0005 | 0.001 | 0.26 |
| 8 | 138092136 | rs35253257 | T | G | 0.0005 | 0.001 | 0.26 |
| 8 | 138095097 | rs4366109 | A | G | 0.0005 | 0.001 | 0.26 |
| 8 | 138097325 | rs2076987 | G | C | 0.0005 | 0.001 | 0.26 |
| 8 | 138099701 | rs1009752441 | C | A | 0.0004 | 0.001 | 0.26 |
| 8 | 138100124 | rs13250544 | G | A | 0.0005 | 0.001 | 0.26 |
| 8 | 138100641 | rs4401897 | A | G | 0.0005 | 0.001 | 0.26 |
| 8 | 138102948 | rs12375407 | C | T | 0.0005 | 0.001 | 0.26 |
| 8 | 138103238 | rs12375349 | G | C | 0.0005 | 0.001 | 0.26 |
| 8 | 138104714 | rs13255859 | T | C | 0.0005 | 0.001 | 0.26 |
| 8 | 138105391 | rs35409626 | A | C | 0.0005 | 0.001 | 0.26 |
| 8 | 138105486 | rs12375361 | C | T | 0.0005 | 0.001 | 0.26 |
| 8 | 138110330 | rs34101782 | C | G | 0.0005 | 0.001 | 0.26 |
| 8 | 138110428 | rs36103261 | A | G | 0.0005 | 0.001 | 0.26 |
| 8 | 138113906 | rs13266672 | A | T | 0.0004 | 0.001 | 0.26 |
| 8 | 138114639 | rs16906760 | C | T | 0.0005 | 0.001 | 0.26 |
| 8 | 138114990 | rs9657449 | A | G | 0.0005 | 0.001 | 0.26 |
| 8 | 138116658 | rs16906762 | T | C | 0.0005 | 0.001 | 0.26 |
| 8 | 138118015 | rs16906764 | G | A | 0.0005 | 0.001 | 0.26 |
| 8 | 138119182 | 8:138119182:TG:[illegible] | T | TG | 0.0003 | 0.001 | 0.26 |
| 8 | 138123015 | rs36076245 | C | G | 0.0003 | 0.001 | 0.28 |
| 8 | 138123150 | rs35201079 | C | T | 0.0003 | 0.001 | 0.28 |
| 8 | 138124046 | rs34561439 | T | A | 0.0003 | 0.001 | 0.28 |
| 8 | 138125859 | 8:138125859:A:<[illegible] | <CN0> | A | 0.0003 | 0.001 | 0.28 |
| 8 | 138132057 | rs13266810 | A | G | 0.0003 | 0.001 | 0.28 |

**Table S17. Credible set of SNPs identified using CAVIAR**

| CHR | BP | SNP | A1 | A2 | Prob_in_pCausalSet | Causal_Post_Prob | r2 |
|---|---|---|---|---|---|---|---|
| 8 | 138133781 | rs13278930 | A | G | 0.0003 | 0.001 | 0.28 |
| 8 | 138134249 | rs34802845 | C | T | 0.0003 | 0.001 | 0.28 |
| 8 | 138139421 | rs79537912 | C | G | 0.0003 | 0.001 | 0.28 |
| 8 | 138139430 | rs77164139 | A | G | 0.0003 | 0.0005 | 0.27 |
| 8 | 138140306 | rs71532115 | G | T | 0.0003 | 0.001 | 0.28 |
| 8 | 138140402 | rs71532116 | T | G | 0.0003 | 0.001 | 0.28 |
| 8 | 138141732 | rs79603374 | G | A | 0.0003 | 0.001 | 0.28 |
| 8 | 138143836 | rs13257805 | T | C | 0.0003 | 0.001 | 0.28 |
| 8 | 138143957 | 8:138143957:AC:A | A | AC | 0.0003 | 0.001 | 0.28 |
| 8 | 138144251 | rs35406480 | C | T | 0.0003 | 0.001 | 0.28 |
| 8 | 138145168 | rs35204735 | G | C | 0.0003 | 0.001 | 0.28 |
| 8 | 138145866 | rs13261322 | G | A | 0.0003 | 0.001 | 0.28 |
| 8 | 138147898 | rs1395122747 | C | T | 0.0003 | 0.001 | 0.28 |
| 8 | 138148461 | rs35751459 | T | C | 0.0003 | 0.001 | 0.28 |
| 8 | 138150662 | rs146391199 | C | T | 0.0003 | 0.001 | 0.28 |
| 8 | 138150772 | rs35123521 | C | T | 0.0003 | 0.001 | 0.28 |
| 8 | 138152537 | rs71532118 | A | G | 0.0003 | 0.001 | 0.28 |
| 8 | 138159522 | rs13275346 | G | C | 0.0003 | 0.001 | 0.28 |
| 8 | 138163740 | rs34250776 | T | A | 0.0004 | 0.001 | 0.33 |
| 8 | 138165302 | rs34679127 | TA | T | 0.0005 | 0.001 | 0.33 |
| 8 | 138176986 | rs71532119 | A | G | 0.001 | 0.001 | 0.38 |
| 8 | 138178588 | rs2091923 | G | A | 0.001 | 0.001 | 0.38 |
| 8 | 138184366 | rs34797832 | C | T | 0.001 | 0.001 | 0.38 |
| 8 | 138186519 | rs13266366 | A | C | 0.001 | 0.002 | 0.37 |
| 8 | 138186852 | rs13276198 | C | T | 0.001 | 0.001 | 0.38 |
| 8 | 138190742 | rs36036943 | A | G | 0.001 | 0.001 | 0.38 |
| 8 | 138190839 | rs71532120 | T | G | 0.001 | 0.001 | 0.38 |
| 8 | 138191583 | rs57275182 | A | G | 0.001 | 0.001 | 0.38 |
| 8 | 138193581 | rs13263868 | A | G | 0.001 | 0.001 | 0.38 |
| 8 | 138193884 | rs34229947 | A | G | 0.001 | 0.002 | 0.37 |

**Table S17. Credible set of SNPs identified using CAVIAR**

| CHR | BP | SNP | A1 | A2 | Prob_in_pCausalSet | Causal_Post_Prob | r2 |
|---|---|---|---|---|---|---|---|
| 8 | 138194801 | rs34136532 | T | C | 0.001 | 0.002 | 0.37 |
| 8 | 138195417 | rs35601795 | C | G | 0.001 | 0.001 | 0.38 |
| 8 | 138195423 | rs34164159 | G | A | 0.001 | 0.002 | 0.37 |
| 8 | 138196442 | rs539732750 | TA | T | 0.001 | 0.002 | 0.37 |
| 8 | 138196686 | rs35469155 | T | C | 0.001 | 0.002 | 0.38 |
| 8 | 138198353 | rs13260372 | A | G | 0.001 | 0.002 | 0.38 |
| 8 | 138198722 | rs71532121 | T | C | 0.001 | 0.002 | 0.38 |
| 8 | 138201893 | rs36041928 | T | A | 0.001 | 0.001 | 0.38 |
| 8 | 138203722 | rs35284528 | G | A | 0.001 | 0.001 | 0.38 |
| 8 | 138204086 | rs56034364 | G | A | 0.001 | 0.001 | 0.38 |
| 8 | 138204459 | rs6993267 | C | G | 0.001 | 0.001 | 0.38 |
| 8 | 138205679 | rs1327465202 | T | C | 0.001 | 0.001 | 0.38 |
| 8 | 138205739 | rs7825732 | C | A | 0.001 | 0.001 | 0.38 |
| 8 | 138206529 | rs13255582 | G | A | 0.001 | 0.001 | 0.38 |
| 8 | 138206735 | rs35116705 | A | T | 0.001 | 0.001 | 0.38 |
| 8 | 138207554 | rs60849715 | A | G | 0.0004 | 0.001 | 0.36 |
| 8 | 138207778 | rs1466052925 | A | AACAC | 0.001 | 0.001 | 0.38 |
| 8 | 138207845 | rs1201942664 | T | TAA | 0.001 | 0.001 | 0.38 |
| 8 | 138207850 | rs34923526 | G | A | 0.001 | 0.001 | 0.38 |
| 8 | 138207967 | rs35624484 | A | G | 0.0004 | 0.001 | 0.36 |
| 8 | 138207978 | rs34352107 | C | T | 0.0004 | 0.001 | 0.36 |
| 8 | 138208072 | rs34796536 | AT | A | 0.0004 | 0.001 | 0.36 |
| 8 | 138208097 | rs10109188 | A | G | 0.0004 | 0.001 | 0.36 |
| 8 | 138208193 | rs10096608 | C | A | 0.0004 | 0.001 | 0.36 |
| 8 | 138208196 | rs10096609 | G | A | 0.0004 | 0.001 | 0.36 |
| 8 | 138209031 | rs7822935 | T | C | 0.0004 | 0.001 | 0.36 |
| 8 | 138209049 | rs7840717 | G | A | 0.0004 | 0.001 | 0.36 |
| 8 | 138209669 | rs7012808 | G | A | 0.001 | 0.002 | 0.40 |
| 8 | 138209837 | rs6982911 | C | T | 0.001 | 0.001 | 0.38 |
| 8 | 138209903 | rs532615715 | GA | G | 0.001 | 0.001 | 0.37 |

**Table S17. Credible set of SNPs identified using CAVIAR**

| CHR | BP | SNP | A1 | A2 | Prob_in_pCausalSet | Causal_Post_Prob | r2 |
|---|---|---|---|---|---|---|---|
| 8 | 138210656 | rs28622139 | C | G | 0.001 | 0.001 | 0.36 |
| **8** | **138229001** | **rs4609227** | **C** | **G** | **0.043** | **0.083** | **1.00** |
| 8 | 138230558 | rs28403940 | C | G | 0.025 | 0.048 | 0.97 |
| 8 | 138234249 | rs80211230 | A | G | 0.025 | 0.048 | 0.97 |
| 8 | 138235253 | rs10112576 | G | A | 0.024 | 0.046 | 0.97 |
| 8 | 138237837 | rs75806625 | A | G | 0.025 | 0.048 | 0.97 |
| 8 | 138244924 | rs6984969 | C | G | 0.025 | 0.048 | 0.97 |
| 8 | 138247025 | rs146779024 | CA | C | 0.017 | 0.033 | 0.95 |
| 8 | 138247237 | rs11166694 | T | A | 0.025 | 0.048 | 0.97 |
| 8 | 138247238 | rs11166695 | A | C | 0.025 | 0.048 | 0.97 |
| 8 | 138249581 | rs11985863 | G | A | 0.004 | 0.007 | 0.81 |
| 8 | 138250392 | rs74886588 | A | G | 0.001 | 0.001 | 0.70 |
| 8 | 138251017 | rs11998305 | T | C | 0.018 | 0.035 | 0.95 |
| 8 | 138252239 | rs73389879 | G | A | 0.018 | 0.035 | 0.95 |
| 8 | 138255624 | rs10094379 | G | A | 0.018 | 0.035 | 0.95 |
| 8 | 138256100 | rs57442651 | C | T | 0.018 | 0.035 | 0.95 |
| 8 | 138257593 | rs59661752 | T | C | 0.0001 | 0.0003 | 0.21 |
| 8 | 138257675 | rs58693023 | G | A | 0.0001 | 0.0003 | 0.21 |
| 8 | 138258042 | rs144388387 | T | G | 0.015 | 0.03 | 0.94 |
| 8 | 138258220 | rs7829774 | C | A | 0.0001 | 0.0003 | 0.21 |
| 8 | 138259895 | rs927781987 | AT | A | 0.0001 | 0.0003 | 0.21 |
| 8 | 138260171 | rs11780629 | A | G | 0.0001 | 0.0003 | 0.21 |
| 8 | 138260456 | rs10113847 | G | A | 0.015 | 0.03 | 0.94 |
| 8 | 138261877 | rs7813071 | G | A | 0.0001 | 0.0003 | 0.21 |
| 8 | 138263163 | rs9886630 | A | C | 0.015 | 0.03 | 0.94 |
| 8 | 138265199 | rs4301477 | T | C | 0.015 | 0.03 | 0.94 |
| 8 | 138265859 | rs10088225 | A | T | 0.015 | 0.03 | 0.94 |
| 8 | 138266983 | rs4634676 | A | G | 0.0001 | 0.0003 | 0.21 |
| 8 | 138267262 | rs77674621 | T | C | 0.015 | 0.03 | 0.94 |
| 8 | 138267684 | rs78339783 | G | T | 0.006 | 0.012 | 0.89 |

**Table S17. Credible set of SNPs identified using CAVIAR**

| CHR | BP | SNP | A1 | A2 | Prob_in_pCausalSet | Causal_Post_Prob | r2 |
|---|---|---|---|---|---|---|---|
| 8 | 138268297 | rs57391177 | A | T | 0.006 | 0.012 | 0.89 |
| 8 | 138268600 | rs61548155 | G | C | 0.015 | 0.03 | 0.94 |
| 8 | 138268930 | rs56907949 | G | T | 0.003 | 0.006 | 0.88 |
| 8 | 138268994 | rs58971409 | C | T | 0.015 | 0.03 | 0.94 |
| 8 | 138271518 | rs7002431 | A | G | 0.015 | 0.03 | 0.94 |
| 8 | 138273862 | rs10505672 | A | G | 0.003 | 0.005 | 0.76 |
| 8 | 138275567 | rs6986961 | T | C | 0.007 | 0.013 | 0.78 |
| 8 | 138278430 | rs7817282 | C | T | 0.0001 | 0.0003 | 0.21 |
| 8 | 138280386 | rs36031258 | CAT | C | 0.005 | 0.01 | 0.69 |
| 8 | 138282708 | rs777367903 | A | T | 0.001 | 0.002 | 0.70 |
| 8 | 138284752 | rs10094924 | C | T | 0.004 | 0.007 | 0.77 |
| 8 | 138285722 | rs4338139 | A | G | 0.004 | 0.007 | 0.77 |
| 8 | 138286203 | rs933952236 | A | C | 0.004 | 0.007 | 0.77 |
| 8 | 138286211 | rs10090816 | A | G | 0.007 | 0.013 | 0.78 |
| 8 | 138286232 | rs7000198 | T | C | 0.004 | 0.007 | 0.77 |
| 8 | 138286602 | rs139017414 | TA | T | 0.007 | 0.013 | 0.78 |
| 8 | 138287048 | rs117105019 | A | G | 0.001 | 0.001 | 0.65 |
| 8 | 138287175 | rs117497573 | T | C | 0.0004 | 0.001 | 0.33 |
| 8 | 138291867 | rs6992584 | T | G | 0.010 | 0.019 | 0.77 |
| 8 | 138297674 | rs11774124 | T | C | 0.004 | 0.007 | 0.67 |
| 8 | 138298903 | rs56200536 | G | C | 0.003 | 0.007 | 0.66 |
| 8 | 138328409 | rs78507288 | A | T | 0.0004 | 0.001 | 0.47 |

**Table S18. Significant eQTL results**

| UniqID | CHR | BP | Database | Tissue | Gene | Symbol | Tested_allele | Signed_stats | P | FDR |
|---|---|---|---|---|---|---|---|---|---|---|
| 4:28470936:A:G | 4 | 28470936 | PsychENCODE | Brain_Cortex | ENSG00000250064 | RP11-123O22.1 | G | -0.32 | 2.83E-22 | 1.83E-19 |
| 4:28472221:A:T | 4 | 28472221 | PsychENCODE | Brain_Cortex | ENSG00000250064 | RP11-123O22.1 | A | -0.31 | 7.26E-22 | 4.57E-19 |
| 4:28473625:G:T | 4 | 28473625 | PsychENCODE | Brain_Cortex | ENSG00000250064 | RP11-123O22.1 | T | -0.31 | 1.05E-21 | 6.57E-19 |
| 4:28473922:C:T | 4 | 28473922 | PsychENCODE | Brain_Cortex | ENSG00000250064 | RP11-123O22.1 | C | -0.31 | 2.36E-21 | 1.44E-18 |
| 4:28474937:C:G | 4 | 28474937 | PsychENCODE | Brain_Cortex | ENSG00000250064 | RP11-123O22.1 | C | -0.31 | 1.33E-19 | 7.14E-17 |
| 4:28476701:A:G | 4 | 28476701 | PsychENCODE | Brain_Cortex | ENSG00000250064 | RP11-123O22.1 | A | -0.31 | 1.28E-19 | 6.88E-17 |
| 4:28476726:A:G | 4 | 28476726 | PsychENCODE | Brain_Cortex | ENSG00000250064 | RP11-123O22.1 | A | -0.31 | 1.24E-19 | 6.65E-17 |
| 4:28476895:A:G | 4 | 28476895 | PsychENCODE | Brain_Cortex | ENSG00000250064 | RP11-123O22.1 | G | -0.31 | 1.31E-19 | 7.03E-17 |
| 4:28482974:A:T | 4 | 28482974 | PsychENCODE | Brain_Cortex | ENSG00000250064 | RP11-123O22.1 | T | -0.29 | 9.09E-18 | 4.24E-15 |
| 4:28484234:C:T | 4 | 28484234 | PsychENCODE | Brain_Cortex | ENSG00000250064 | RP11-123O22.1 | C | -0.29 | 3.69E-18 | 1.77E-15 |
| 4:28484389:G:T | 4 | 28484389 | PsychENCODE | Brain_Cortex | ENSG00000250064 | RP11-123O22.1 | G | -0.29 | 3.67E-18 | 1.76E-15 |
| 4:28488065:A:G | 4 | 28488065 | PsychENCODE | Brain_Cortex | ENSG00000250064 | RP11-123O22.1 | A | -0.29 | 1.57E-17 | 7.17E-15 |
| 4:28490381:C:T | 4 | 28490381 | PsychENCODE | Brain_Cortex | ENSG00000250064 | RP11-123O22.1 | C | -0.29 | 1.87E-17 | 8.50E-15 |
| 4:28493198:A:G | 4 | 28493198 | PsychENCODE | Brain_Cortex | ENSG00000250064 | RP11-123O22.1 | G | -0.29 | 5.89E-18 | 2.78E-15 |
| 4:28493840:C:G | 4 | 28493840 | PsychENCODE | Brain_Cortex | ENSG00000250064 | RP11-123O22.1 | C | -0.29 | 6.21E-18 | 2.93E-15 |
| 4:28494313:A:G | 4 | 28494313 | PsychENCODE | Brain_Cortex | ENSG00000250064 | RP11-123O22.1 | A | -0.29 | 6.53E-18 | 3.08E-15 |
| 4:28496256:A:G | 4 | 28496256 | PsychENCODE | Brain_Cortex | ENSG00000250064 | RP11-123O22.1 | A | -0.29 | 7.56E-18 | 3.55E-15 |
| 4:28497583:C:T | 4 | 28497583 | PsychENCODE | Brain_Cortex | ENSG00000250064 | RP11-123O22.1 | T | -0.28 | 3.70E-17 | 1.64E-14 |
| 4:28498910:A:G | 4 | 28498910 | PsychENCODE | Brain_Cortex | ENSG00000250064 | RP11-123O22.1 | G | -0.29 | 1.06E-17 | 4.90E-15 |
| 4:28499782:A:G | 4 | 28499782 | PsychENCODE | Brain_Cortex | ENSG00000250064 | RP11-123O22.1 | G | -0.29 | 1.20E-17 | 5.53E-15 |
| 4:28503151:C:T | 4 | 28503151 | PsychENCODE | Brain_Cortex | ENSG00000250064 | RP11-123O22.1 | T | -0.29 | 6.80E-18 | 3.20E-15 |
| 4:28506937:G:T | 4 | 28506937 | PsychENCODE | Brain_Cortex | ENSG00000250064 | RP11-123O22.1 | T | -0.27 | 7.11E-17 | 3.09E-14 |
| 4:28507335:A:G | 4 | 28507335 | PsychENCODE | Brain_Cortex | ENSG00000250064 | RP11-123O22.1 | G | -0.29 | 8.30E-18 | 3.88E-15 |
| 4:28507573:C:T | 4 | 28507573 | PsychENCODE | Brain_Cortex | ENSG00000250064 | RP11-123O22.1 | T | -0.29 | 8.32E-18 | 3.89E-15 |
| 4:28507739:A:G | 4 | 28507739 | PsychENCODE | Brain_Cortex | ENSG00000250064 | RP11-123O22.1 | A | -0.27 | 7.34E-17 | 3.19E-14 |
| 4:28509079:A:C | 4 | 28509079 | PsychENCODE | Brain_Cortex | ENSG00000250064 | RP11-123O22.1 | A | -0.29 | 9.10E-18 | 4.24E-15 |
| 4:28518533:C:G | 4 | 28518533 | PsychENCODE | Brain_Cortex | ENSG00000250064 | RP11-123O22.1 | G | -0.29 | 1.71E-17 | 7.81E-15 |
| 4:28519470:A:G | 4 | 28519470 | PsychENCODE | Brain_Cortex | ENSG00000250064 | RP11-123O22.1 | G | -0.29 | 1.88E-17 | 8.53E-15 |
| 4:28520773:A:C | 4 | 28520773 | PsychENCODE | Brain_Cortex | ENSG00000250064 | RP11-123O22.1 | C | -0.29 | 8.90E-18 | 4.16E-15 |
| 4:28522519:C:T | 4 | 28522519 | PsychENCODE | Brain_Cortex | ENSG00000250064 | RP11-123O22.1 | C | -0.28 | 3.79E-17 | 1.68E-14 |
| 4:28524559:C:T | 4 | 28524559 | PsychENCODE | Brain_Cortex | ENSG00000250064 | RP11-123O22.1 | T | -0.28 | 4.18E-17 | 1.85E-14 |
| 4:28524852:A:G | 4 | 28524852 | PsychENCODE | Brain_Cortex | ENSG00000250064 | RP11-123O22.1 | G | 0.18 | 8.97E-11 | 2.17E-08 |
| 4:28527861:A:G | 4 | 28527861 | PsychENCODE | Brain_Cortex | ENSG00000250064 | RP11-123O22.1 | G | 0.18 | 8.49E-11 | 2.06E-08 |
| 4:28528060:C:T | 4 | 28528060 | PsychENCODE | Brain_Cortex | ENSG00000250064 | RP11-123O22.1 | C | -0.28 | 7.15E-17 | 3.10E-14 |
| 4:28528234:A:T | 4 | 28528234 | PsychENCODE | Brain_Cortex | ENSG00000250064 | RP11-123O22.1 | A | -0.19 | 1.27E-11 | 3.40E-09 |
| 4:28532565:C:T | 4 | 28532565 | PsychENCODE | Brain_Cortex | ENSG00000250064 | RP11-123O22.1 | C | -0.28 | 2.02E-16 | 8.46E-14 |
| 4:28538250:C:G | 4 | 28538250 | PsychENCODE | Brain_Cortex | ENSG00000250064 | RP11-123O22.1 | G | -0.28 | 1.07E-16 | 4.57E-14 |
| 4:28538736:A:G | 4 | 28538736 | PsychENCODE | Brain_Cortex | ENSG00000250064 | RP11-123O22.1 | G | -0.28 | 2.60E-16 | 1.08E-13 |

**Table S18. Significant eQTL results**

| UniqID | CHR | BP | Database | Tissue | Gene | Symbol | Tested_allele | Signed_stats | P | FDR |
|---|---|---|---|---|---|---|---|---|---|---|
| 4:28539924:A:G | 4 | 28539924 | PsychENCODE | Brain_Cortex | ENSG00000250064 | RP11-123O22.1 | A | -0.19 | 9.36E-11 | 2.26E-08 |
| 4:28540930:A:C | 4 | 28540930 | PsychENCODE | Brain_Cortex | ENSG00000250064 | RP11-123O22.1 | C | -0.28 | 2.56E-16 | 1.06E-13 |
| 4:28542348:C:G | 4 | 28542348 | PsychENCODE | Brain_Cortex | ENSG00000250064 | RP11-123O22.1 | G | -0.28 | 4.51E-16 | 1.84E-13 |
| 4:28542928:A:G | 4 | 28542928 | PsychENCODE | Brain_Cortex | ENSG00000250064 | RP11-123O22.1 | A | -0.28 | 1.77E-16 | 7.46E-14 |
| 4:28545048:A:G | 4 | 28545048 | PsychENCODE | Brain_Cortex | ENSG00000250064 | RP11-123O22.1 | G | -0.27 | 1.23E-15 | 4.84E-13 |
| 4:28557165:A:G | 4 | 28557165 | PsychENCODE | Brain_Cortex | ENSG00000250064 | RP11-123O22.1 | G | -0.27 | 1.42E-15 | 5.54E-13 |
| 4:28558043:A:G | 4 | 28558043 | PsychENCODE | Brain_Cortex | ENSG00000250064 | RP11-123O22.1 | G | -0.27 | 7.34E-16 | 2.94E-13 |
| 4:28560540:A:G | 4 | 28560540 | PsychENCODE | Brain_Cortex | ENSG00000250064 | RP11-123O22.1 | A | -0.27 | 7.39E-16 | 2.96E-13 |
| 4:28561303:A:G | 4 | 28561303 | PsychENCODE | Brain_Cortex | ENSG00000250064 | RP11-123O22.1 | A | -0.27 | 1.28E-15 | 5.00E-13 |
| 4:28561374:C:T | 4 | 28561374 | PsychENCODE | Brain_Cortex | ENSG00000250064 | RP11-123O22.1 | T | -0.27 | 7.37E-16 | 2.95E-13 |
| 4:28562034:C:T | 4 | 28562034 | PsychENCODE | Brain_Cortex | ENSG00000250064 | RP11-123O22.1 | C | -0.27 | 7.40E-16 | 2.96E-13 |
| 4:28563166:A:G | 4 | 28563166 | PsychENCODE | Brain_Cortex | ENSG00000250064 | RP11-123O22.1 | A | -0.27 | 7.40E-16 | 2.96E-13 |
| 4:28563313:A:G | 4 | 28563313 | PsychENCODE | Brain_Cortex | ENSG00000250064 | RP11-123O22.1 | A | -0.27 | 7.42E-16 | 2.97E-13 |
| 4:28563413:C:T | 4 | 28563413 | PsychENCODE | Brain_Cortex | ENSG00000250064 | RP11-123O22.1 | C | -0.27 | 7.42E-16 | 2.97E-13 |
| 4:28565123:A:C | 4 | 28565123 | PsychENCODE | Brain_Cortex | ENSG00000250064 | RP11-123O22.1 | A | -0.27 | 7.40E-16 | 2.96E-13 |
| 4:28565233:A:G | 4 | 28565233 | PsychENCODE | Brain_Cortex | ENSG00000250064 | RP11-123O22.1 | G | -0.27 | 7.39E-16 | 2.96E-13 |
| 4:28566571:C:T | 4 | 28566571 | PsychENCODE | Brain_Cortex | ENSG00000250064 | RP11-123O22.1 | C | -0.27 | 7.33E-16 | 2.93E-13 |
| 4:28566686:G:T | 4 | 28566686 | PsychENCODE | Brain_Cortex | ENSG00000250064 | RP11-123O22.1 | T | -0.27 | 7.32E-16 | 2.93E-13 |
| 4:28568637:C:T | 4 | 28568637 | PsychENCODE | Brain_Cortex | ENSG00000250064 | RP11-123O22.1 | C | -0.27 | 6.83E-16 | 2.74E-13 |
| 4:28568717:C:G | 4 | 28568717 | PsychENCODE | Brain_Cortex | ENSG00000250064 | RP11-123O22.1 | C | -0.27 | 8.73E-16 | 3.47E-13 |
| 4:28569819:G:T | 4 | 28569819 | PsychENCODE | Brain_Cortex | ENSG00000250064 | RP11-123O22.1 | T | -0.28 | 5.84E-16 | 2.36E-13 |
| 4:28570278:G:T | 4 | 28570278 | PsychENCODE | Brain_Cortex | ENSG00000250064 | RP11-123O22.1 | T | -0.28 | 5.40E-16 | 2.19E-13 |
| 4:28571606:C:T | 4 | 28571606 | PsychENCODE | Brain_Cortex | ENSG00000250064 | RP11-123O22.1 | T | -0.28 | 4.81E-16 | 1.95E-13 |
| 4:28571640:C:T | 4 | 28571640 | PsychENCODE | Brain_Cortex | ENSG00000250064 | RP11-123O22.1 | T | -0.28 | 6.62E-16 | 2.66E-13 |
| 4:28571652:G:T | 4 | 28571652 | PsychENCODE | Brain_Cortex | ENSG00000250064 | RP11-123O22.1 | G | -0.27 | 1.54E-15 | 5.98E-13 |
| 4:28571988:A:G | 4 | 28571988 | PsychENCODE | Brain_Cortex | ENSG00000250064 | RP11-123O22.1 | G | -0.28 | 4.54E-16 | 1.85E-13 |
| 4:28572856:A:G | 4 | 28572856 | PsychENCODE | Brain_Cortex | ENSG00000250064 | RP11-123O22.1 | A | -0.28 | 4.24E-16 | 1.73E-13 |
| 4:28575113:C:T | 4 | 28575113 | PsychENCODE | Brain_Cortex | ENSG00000250064 | RP11-123O22.1 | T | -0.15 | 9.01E-06 | 0.001 |
| 4:28575292:C:T | 4 | 28575292 | PsychENCODE | Brain_Cortex | ENSG00000250064 | RP11-123O22.1 | T | -0.15 | 8.81E-06 | 0.001 |
| 4:28575709:A:C | 4 | 28575709 | PsychENCODE | Brain_Cortex | ENSG00000250064 | RP11-123O22.1 | C | -0.15 | 8.81E-06 | 0.001 |
| 4:28576379:A:C | 4 | 28576379 | PsychENCODE | Brain_Cortex | ENSG00000250064 | RP11-123O22.1 | C | -0.15 | 8.82E-06 | 0.001 |
| 4:28577504:C:G | 4 | 28577504 | PsychENCODE | Brain_Cortex | ENSG00000250064 | RP11-123O22.1 | G | -0.22 | 4.28E-09 | 8.39E-07 |
| 4:28581335:A:C | 4 | 28581335 | PsychENCODE | Brain_Cortex | ENSG00000250064 | RP11-123O22.1 | A | -0.15 | 9.41E-06 | 0.001 |
| 4:28581431:A:T | 4 | 28581431 | PsychENCODE | Brain_Cortex | ENSG00000250064 | RP11-123O22.1 | A | -0.25 | 8.66E-10 | 1.86E-07 |
| 4:28583060:A:G | 4 | 28583060 | PsychENCODE | Brain_Cortex | ENSG00000250064 | RP11-123O22.1 | A | -0.22 | 4.22E-09 | 8.27E-07 |
| 4:28584417:A:G | 4 | 28584417 | PsychENCODE | Brain_Cortex | ENSG00000250064 | RP11-123O22.1 | A | -0.22 | 8.51E-09 | 1.60E-06 |
| 4:28587846:C:G | 4 | 28587846 | PsychENCODE | Brain_Cortex | ENSG00000250064 | RP11-123O22.1 | G | -0.22 | 1.42E-08 | 2.58E-06 |
| 4:28589079:C:T | 4 | 28589079 | PsychENCODE | Brain_Cortex | ENSG00000250064 | RP11-123O22.1 | T | -0.25 | 1.04E-09 | 2.20E-07 |

**Table S18. Significant eQTL results**

| UniqID | CHR | BP | Database | Tissue | Gene | Symbol | Tested_allele | Signed_stats | P | FDR |
|---|---|---|---|---|---|---|---|---|---|---|
| 4:28589619:A:G | 4 | 28589619 | PsychENCODE | Brain_Cortex | ENSG00000250064 | RP11-123O22.1 | A | -0.25 | 1.04E-09 | 2.22E-07 |
| 4:28591924:A:G | 4 | 28591924 | PsychENCODE | Brain_Cortex | ENSG00000250064 | RP11-123O22.1 | A | -0.25 | 1.10E-09 | 2.33E-07 |
| 4:28593164:C:T | 4 | 28593164 | PsychENCODE | Brain_Cortex | ENSG00000250064 | RP11-123O22.1 | C | -0.25 | 1.12E-09 | 2.36E-07 |
| 4:28593978:C:T | 4 | 28593978 | PsychENCODE | Brain_Cortex | ENSG00000250064 | RP11-123O22.1 | C | -0.25 | 1.10E-09 | 2.34E-07 |
| 4:28594111:A:G | 4 | 28594111 | PsychENCODE | Brain_Cortex | ENSG00000250064 | RP11-123O22.1 | A | -0.25 | 1.10E-09 | 2.33E-07 |
| 4:28595064:A:G | 4 | 28595064 | PsychENCODE | Brain_Cortex | ENSG00000250064 | RP11-123O22.1 | G | -0.25 | 1.10E-09 | 2.33E-07 |
| 4:28596316:C:T | 4 | 28596316 | PsychENCODE | Brain_Cortex | ENSG00000250064 | RP11-123O22.1 | C | -0.25 | 1.10E-09 | 2.33E-07 |
| 4:28597991:C:T | 4 | 28597991 | PsychENCODE | Brain_Cortex | ENSG00000250064 | RP11-123O22.1 | C | -0.22 | 1.41E-08 | 2.57E-06 |
| 4:28598358:C:T | 4 | 28598358 | PsychENCODE | Brain_Cortex | ENSG00000250064 | RP11-123O22.1 | C | -0.25 | 1.10E-09 | 2.33E-07 |
| 4:28600877:C:G | 4 | 28600877 | PsychENCODE | Brain_Cortex | ENSG00000250064 | RP11-123O22.1 | C | -0.25 | 1.10E-09 | 2.33E-07 |
| 4:28616198:A:G | 4 | 28616198 | PsychENCODE | Brain_Cortex | ENSG00000250064 | RP11-123O22.1 | G | 0.25 | 1.74E-09 | 3.59E-07 |
| 4:28617013:C:G | 4 | 28617013 | PsychENCODE | Brain_Cortex | ENSG00000250064 | RP11-123O22.1 | C | 0.13 | 5.15E-05 | 0.0047 |
| 4:28618060:G:T | 4 | 28618060 | PsychENCODE | Brain_Cortex | ENSG00000250064 | RP11-123O22.1 | G | 0.25 | 1.73E-09 | 3.56E-07 |
| 4:28620849:A:T | 4 | 28620849 | PsychENCODE | Brain_Cortex | ENSG00000250064 | RP11-123O22.1 | A | 0.14 | 3.40E-05 | 0.0033 |
| 4:28621704:C:T | 4 | 28621704 | PsychENCODE | Brain_Cortex | ENSG00000250064 | RP11-123O22.1 | C | 0.25 | 1.77E-09 | 3.65E-07 |
| 4:28623448:A:G | 4 | 28623448 | PsychENCODE | Brain_Cortex | ENSG00000250064 | RP11-123O22.1 | A | 0.25 | 1.75E-09 | 3.61E-07 |
| 4:28623645:G:T | 4 | 28623645 | PsychENCODE | Brain_Cortex | ENSG00000250064 | RP11-123O22.1 | G | 0.25 | 1.75E-09 | 3.61E-07 |
| 4:28624594:G:T | 4 | 28624594 | PsychENCODE | Brain_Cortex | ENSG00000250064 | RP11-123O22.1 | G | 0.25 | 1.71E-09 | 3.53E-07 |
| 4:28626286:A:C | 4 | 28626286 | PsychENCODE | Brain_Cortex | ENSG00000250064 | RP11-123O22.1 | C | 0.25 | 1.60E-09 | 3.31E-07 |
| 4:28629058:C:G | 4 | 28629058 | PsychENCODE | Brain_Cortex | ENSG00000250064 | RP11-123O22.1 | C | 0.25 | 1.48E-09 | 3.07E-07 |
| 4:28629539:C:T | 4 | 28629539 | PsychENCODE | Brain_Cortex | ENSG00000250064 | RP11-123O22.1 | C | 0.25 | 1.45E-09 | 3.01E-07 |
| 4:28631217:A:G | 4 | 28631217 | PsychENCODE | Brain_Cortex | ENSG00000250064 | RP11-123O22.1 | A | 0.25 | 1.41E-09 | 2.94E-07 |
| 4:28633364:G:T | 4 | 28633364 | PsychENCODE | Brain_Cortex | ENSG00000250064 | RP11-123O22.1 | G | 0.25 | 1.33E-09 | 2.78E-07 |
| 4:28633813:C:T | 4 | 28633813 | PsychENCODE | Brain_Cortex | ENSG00000250064 | RP11-123O22.1 | C | 0.25 | 1.02E-09 | 2.17E-07 |
| 4:28651223:A:G | 4 | 28651223 | PsychENCODE | Brain_Cortex | ENSG00000250064 | RP11-123O22.1 | G | 0.25 | 8.95E-10 | 1.92E-07 |
| 4:28651305:C:T | 4 | 28651305 | PsychENCODE | Brain_Cortex | ENSG00000250064 | RP11-123O22.1 | T | 0.25 | 8.96E-10 | 1.92E-07 |
| 4:28652801:C:T | 4 | 28652801 | PsychENCODE | Brain_Cortex | ENSG00000250064 | RP11-123O22.1 | T | 0.25 | 9.01E-10 | 1.93E-07 |
| 4:28653531:C:T | 4 | 28653531 | PsychENCODE | Brain_Cortex | ENSG00000250064 | RP11-123O22.1 | C | 0.25 | 9.00E-10 | 1.93E-07 |
| 4:28656016:A:C | 4 | 28656016 | PsychENCODE | Brain_Cortex | ENSG00000250064 | RP11-123O22.1 | A | 0.25 | 8.72E-10 | 1.87E-07 |
| 4:28656017:G:T | 4 | 28656017 | PsychENCODE | Brain_Cortex | ENSG00000250064 | RP11-123O22.1 | T | 0.25 | 8.72E-10 | 1.87E-07 |
| 4:28662735:C:T | 4 | 28662735 | PsychENCODE | Brain_Cortex | ENSG00000250064 | RP11-123O22.1 | C | 0.25 | 1.30E-09 | 2.73E-07 |
| 4:28663069:C:G | 4 | 28663069 | PsychENCODE | Brain_Cortex | ENSG00000250064 | RP11-123O22.1 | C | 0.25 | 1.12E-09 | 2.38E-07 |
| 4:28666576:A:G | 4 | 28666576 | PsychENCODE | Brain_Cortex | ENSG00000250064 | RP11-123O22.1 | A | 0.25 | 1.06E-09 | 2.25E-07 |
| 4:28666876:C:G | 4 | 28666876 | PsychENCODE | Brain_Cortex | ENSG00000250064 | RP11-123O22.1 | G | 0.25 | 1.05E-09 | 2.23E-07 |
| 4:28671928:A:G | 4 | 28671928 | PsychENCODE | Brain_Cortex | ENSG00000250064 | RP11-123O22.1 | A | 0.25 | 9.70E-10 | 2.07E-07 |
| 4:28673636:A:C | 4 | 28673636 | PsychENCODE | Brain_Cortex | ENSG00000250064 | RP11-123O22.1 | A | 0.13 | 6.80E-05 | 0.006 |
| 4:28676223:C:T | 4 | 28676223 | PsychENCODE | Brain_Cortex | ENSG00000250064 | RP11-123O22.1 | C | 0.25 | 9.00E-10 | 1.93E-07 |
| 4:28676642:A:G | 4 | 28676642 | PsychENCODE | Brain_Cortex | ENSG00000250064 | RP11-123O22.1 | A | 0.25 | 8.94E-10 | 1.91E-07 |

**Table S18. Significant eQTL results**

| UniqID | CHR | BP | Database | Tissue | Gene | Symbol | Tested_allele | Signed_stats | P | FDR |
|---|---|---|---|---|---|---|---|---|---|---|
| 4:28681385:G:T | 4 | 28681385 | PsychENCODE | Brain_Cortex | ENSG00000250064 | RP11-123O22.1 | G | 0.25 | 8.38E-10 | 1.80E-07 |
| 4:28686903:A:G | 4 | 28686903 | PsychENCODE | Brain_Cortex | ENSG00000250064 | RP11-123O22.1 | A | 0.25 | 8.11E-10 | 1.75E-07 |
| 4:28687841:C:T | 4 | 28687841 | PsychENCODE | Brain_Cortex | ENSG00000250064 | RP11-123O22.1 | C | 0.25 | 8.12E-10 | 1.75E-07 |
| 4:28690532:G:T | 4 | 28690532 | PsychENCODE | Brain_Cortex | ENSG00000250064 | RP11-123O22.1 | T | 0.19 | 3.86E-07 | 5.58E-05 |
| 4:28692671:A:G | 4 | 28692671 | PsychENCODE | Brain_Cortex | ENSG00000250064 | RP11-123O22.1 | G | 0.25 | 8.89E-10 | 1.90E-07 |
| 4:28696268:A:G | 4 | 28696268 | PsychENCODE | Brain_Cortex | ENSG00000250064 | RP11-123O22.1 | G | 0.25 | 9.83E-10 | 2.09E-07 |
| 4:28696850:A:T | 4 | 28696850 | PsychENCODE | Brain_Cortex | ENSG00000250064 | RP11-123O22.1 | T | 0.24 | 2.36E-09 | 4.79E-07 |
| 4:28698177:C:T | 4 | 28698177 | PsychENCODE | Brain_Cortex | ENSG00000250064 | RP11-123O22.1 | C | 0.25 | 1.03E-09 | 2.18E-07 |
| 4:28699965:C:T | 4 | 28699965 | PsychENCODE | Brain_Cortex | ENSG00000250064 | RP11-123O22.1 | C | 0.25 | 1.09E-09 | 2.30E-07 |
| 4:28701658:C:G | 4 | 28701658 | PsychENCODE | Brain_Cortex | ENSG00000250064 | RP11-123O22.1 | C | 0.25 | 1.14E-09 | 2.41E-07 |
| 4:28710551:A:T | 4 | 28710551 | PsychENCODE | Brain_Cortex | ENSG00000250064 | RP11-123O22.1 | T | 0.25 | 1.08E-09 | 2.29E-07 |
| 4:28712457:A:G | 4 | 28712457 | PsychENCODE | Brain_Cortex | ENSG00000250064 | RP11-123O22.1 | A | 0.25 | 1.20E-09 | 2.52E-07 |
| 4:28714432:A:T | 4 | 28714432 | PsychENCODE | Brain_Cortex | ENSG00000250064 | RP11-123O22.1 | T | 0.25 | 1.11E-09 | 2.34E-07 |
| 4:28717531:A:G | 4 | 28717531 | PsychENCODE | Brain_Cortex | ENSG00000250064 | RP11-123O22.1 | G | 0.25 | 1.20E-09 | 2.53E-07 |
| 4:28719509:C:T | 4 | 28719509 | PsychENCODE | Brain_Cortex | ENSG00000250064 | RP11-123O22.1 | T | 0.24 | 3.24E-09 | 6.44E-07 |
| 4:28720102:A:G | 4 | 28720102 | PsychENCODE | Brain_Cortex | ENSG00000250064 | RP11-123O22.1 | G | 0.24 | 3.25E-09 | 6.47E-07 |
| 4:28720915:A:G | 4 | 28720915 | PsychENCODE | Brain_Cortex | ENSG00000250064 | RP11-123O22.1 | G | 0.25 | 1.14E-09 | 2.41E-07 |
| 4:28721385:C:T | 4 | 28721385 | PsychENCODE | Brain_Cortex | ENSG00000250064 | RP11-123O22.1 | C | 0.24 | 3.15E-09 | 6.28E-07 |
| 4:28722300:A:G | 4 | 28722300 | PsychENCODE | Brain_Cortex | ENSG00000250064 | RP11-123O22.1 | G | 0.25 | 8.91E-10 | 1.91E-07 |
| 4:28723616:G:T | 4 | 28723616 | PsychENCODE | Brain_Cortex | ENSG00000250064 | RP11-123O22.1 | G | 0.25 | 1.07E-09 | 2.28E-07 |
| 4:28724974:A:G | 4 | 28724974 | PsychENCODE | Brain_Cortex | ENSG00000250064 | RP11-123O22.1 | A | 0.25 | 9.96E-10 | 2.12E-07 |
| 4:28727427:A:G | 4 | 28727427 | PsychENCODE | Brain_Cortex | ENSG00000250064 | RP11-123O22.1 | A | -0.24 | 1.90E-07 | 2.89E-05 |
| 4:28735828:C:T | 4 | 28735828 | PsychENCODE | Brain_Cortex | ENSG00000250064 | RP11-123O22.1 | T | -0.25 | 1.10E-07 | 1.74E-05 |
| 4:28736036:A:C | 4 | 28736036 | PsychENCODE | Brain_Cortex | ENSG00000250064 | RP11-123O22.1 | A | -0.15 | 3.21E-05 | 0.0031 |
| 4:28736038:A:G | 4 | 28736038 | PsychENCODE | Brain_Cortex | ENSG00000250064 | RP11-123O22.1 | A | -0.25 | 1.46E-07 | 2.27E-05 |
| 4:28736971:A:T | 4 | 28736971 | PsychENCODE | Brain_Cortex | ENSG00000250064 | RP11-123O22.1 | A | -0.15 | 2.72E-05 | 0.0027 |
| 4:28737112:C:T | 4 | 28737112 | PsychENCODE | Brain_Cortex | ENSG00000250064 | RP11-123O22.1 | C | -0.25 | 1.12E-07 | 1.78E-05 |
| 4:28740012:A:C | 4 | 28740012 | PsychENCODE | Brain_Cortex | ENSG00000250064 | RP11-123O22.1 | A | -0.26 | 6.46E-08 | 1.06E-05 |
| 4:28747310:A:G | 4 | 28747310 | PsychENCODE | Brain_Cortex | ENSG00000250064 | RP11-123O22.1 | A | -0.17 | 1.29E-06 | 0.00017 |
| 4:28748805:A:G | 4 | 28748805 | PsychENCODE | Brain_Cortex | ENSG00000250064 | RP11-123O22.1 | A | -0.26 | 7.51E-08 | 1.22E-05 |
| 4:28749863:A:T | 4 | 28749863 | PsychENCODE | Brain_Cortex | ENSG00000250064 | RP11-123O22.1 | T | -0.18 | 4.47E-07 | 6.38E-05 |
| 4:28750665:C:T | 4 | 28750665 | PsychENCODE | Brain_Cortex | ENSG00000250064 | RP11-123O22.1 | T | -0.25 | 7.74E-08 | 1.26E-05 |
| 4:28754779:A:T | 4 | 28754779 | PsychENCODE | Brain_Cortex | ENSG00000250064 | RP11-123O22.1 | T | -0.21 | 7.91E-09 | 1.49E-06 |
| 4:28761781:A:G | 4 | 28761781 | PsychENCODE | Brain_Cortex | ENSG00000250064 | RP11-123O22.1 | A | -0.23 | 4.90E-09 | 9.52E-07 |
| 4:28762951:C:T | 4 | 28762951 | PsychENCODE | Brain_Cortex | ENSG00000250064 | RP11-123O22.1 | T | -0.26 | 3.29E-08 | 5.66E-06 |
| 4:28767776:A:G | 4 | 28767776 | PsychENCODE | Brain_Cortex | ENSG00000250064 | RP11-123O22.1 | A | -0.26 | 3.53E-08 | 6.04E-06 |
| 4:28771767:A:T | 4 | 28771767 | PsychENCODE | Brain_Cortex | ENSG00000250064 | RP11-123O22.1 | A | -0.26 | 3.72E-08 | 6.36E-06 |
| 4:28774747:A:G | 4 | 28774747 | PsychENCODE | Brain_Cortex | ENSG00000250064 | RP11-123O22.1 | A | -0.26 | 4.03E-08 | 6.84E-06 |

**Table S18. Significant eQTL results**

| UniqID | CHR | BP | Database | Tissue | Gene | Symbol | Tested_allele | Signed_stats | P | FDR |
|---|---|---|---|---|---|---|---|---|---|---|
| 4:28775853:A:G | 4 | 28775853 | PsychENCODE | Brain_Cortex | ENSG00000250064 | RP11-123O22.1 | G | -0.26 | 5.25E-08 | 8.75E-06 |
| 4:28779671:A:G | 4 | 28779671 | PsychENCODE | Brain_Cortex | ENSG00000250064 | RP11-123O22.1 | A | -0.26 | 4.00E-08 | 6.80E-06 |
| 4:28781817:A:T | 4 | 28781817 | PsychENCODE | Brain_Cortex | ENSG00000250064 | RP11-123O22.1 | T | -0.26 | 5.41E-08 | 9.00E-06 |
| 4:28788513:A:T | 4 | 28788513 | PsychENCODE | Brain_Cortex | ENSG00000250064 | RP11-123O22.1 | A | -0.26 | 4.48E-08 | 7.55E-06 |
| 4:28791121:C:T | 4 | 28791121 | PsychENCODE | Brain_Cortex | ENSG00000250064 | RP11-123O22.1 | C | -0.27 | 2.70E-08 | 4.71E-06 |
| 4:28792788:C:T | 4 | 28792788 | PsychENCODE | Brain_Cortex | ENSG00000250064 | RP11-123O22.1 | C | -0.27 | 2.63E-08 | 4.60E-06 |
| 4:28793330:A:G | 4 | 28793330 | PsychENCODE | Brain_Cortex | ENSG00000250064 | RP11-123O22.1 | A | -0.27 | 2.12E-08 | 3.75E-06 |
| 4:28795187:C:T | 4 | 28795187 | PsychENCODE | Brain_Cortex | ENSG00000250064 | RP11-123O22.1 | T | -0.27 | 1.87E-08 | 3.34E-06 |
| 4:28799742:C:T | 4 | 28799742 | PsychENCODE | Brain_Cortex | ENSG00000250064 | RP11-123O22.1 | C | -0.16 | 2.75E-05 | 0.0027 |
| 4:28801221:C:T | 4 | 28801221 | PsychENCODE | Brain_Cortex | ENSG00000250064 | RP11-123O22.1 | C | -0.27 | 1.39E-08 | 2.53E-06 |
| 4:28812966:A:T | 4 | 28812966 | PsychENCODE | Brain_Cortex | ENSG00000250064 | RP11-123O22.1 | A | -0.28 | 2.97E-08 | 5.15E-06 |
| 4:28813116:A:C | 4 | 28813116 | PsychENCODE | Brain_Cortex | ENSG00000250064 | RP11-123O22.1 | A | -0.28 | 2.84E-08 | 4.93E-06 |
| 4:28813302:C:T | 4 | 28813302 | PsychENCODE | Brain_Cortex | ENSG00000250064 | RP11-123O22.1 | T | -0.28 | 2.93E-08 | 5.08E-06 |
| 4:28820334:C:T | 4 | 28820334 | PsychENCODE | Brain_Cortex | ENSG00000250064 | RP11-123O22.1 | T | -0.27 | 1.26E-08 | 2.30E-06 |
| 4:28827186:C:T | 4 | 28827186 | PsychENCODE | Brain_Cortex | ENSG00000250064 | RP11-123O22.1 | T | -0.28 | 3.54E-08 | 6.07E-06 |
| 4:28829722:A:G | 4 | 28829722 | PsychENCODE | Brain_Cortex | ENSG00000250064 | RP11-123O22.1 | A | -0.28 | 3.42E-08 | 5.87E-06 |
| 4:28466655:A:ATAT | 4 | 28466655 | GTEx/v8 | Brain_Cortex | ENSG00000249228 | RP11-769N22.1 | A | -0.45 | 3.15E-06 | 0.00014 |
| 4:28473922:C:T | 4 | 28473922 | GTEx/v8 | Brain_Cortex | ENSG00000249228 | RP11-769N22.1 | C | -0.43 | 5.98E-05 | 0.00014 |

**Table S19. Association between *MIR4275* rs292034 and spherical power in children**

| | | | | | HKCES 1 | | | | | | HKCES 2 | | | | | | Meta children | | | |
|---|---|---|---|---|---|---|---|---|---|---|---|---|---|---|---|---|---|---|---|---|
| **CHR** | **SNP** | **BP** | **EA** | **NEA** | **EAF** | **BETA** | **SE** | **L95** | **U95** | **P[a]** | **EAF** | **BETA** | **SE** | **L95** | **U95** | **P[a]** | **BETA** | **P** | **Q test** | **$I^2$ (%)** |
| 4 | rs292034 | 28478119 | G | T | 0.013 | -1.43 | 0.32 | -2.06 | -0.81 | $8.29\times10^{-6}$ | 0.011 | -0.59 | 0.2 | -0.99 | -0.19 | 0.0037 | -0.83 | $1.18\times10^{-6}$ | 0.02 | 79 |

[a]Results were adjusted for age and sex. $I^2$ (%), Heterogeneity.

**Table S20. Association between *MIR4275* rs292034 and corneal astigmatism in children**

| | | | | | HKCES 1 | | | | | | HKCES 2 | | | | | | Meta children | | | |
|---|---|---|---|---|---|---|---|---|---|---|---|---|---|---|---|---|---|---|---|---|
| **CHR** | **SNP** | **BP** | **EA** | **NEA** | **EAF** | **OR** | **SE** | **L95** | **U95** | **P[a]** | **EAF** | **OR** | **SE** | **L95** | **U95** | **P[a]** | **OR** | **P** | **Q test** | **$I^2$ (%)** |
| 4 | rs292034 | 28478119 | G | T | 0.013 | 1.64 | 0.46 | 0.66 | 4.06 | 0.29 | 0.011 | 2.1 | 0.34 | 1.09 | 4.05 | 0.027 | 1.93 | 0.016 | 0.66 | 0 |

[a]Results were adjusted for age, sex and spherical equivalent. $I^2$ (%), Heterogeneity.

Corneal astigmatism  was expressed using positive notation and defined as astigmatism of 1.0 D or greater.

**Table S21. Study cohorts description**

| **Hong Kong Children Eye Study (HKCES)[1]** |
|---|
| HKCES is an ongoing population-based study focusing on eye conditions in primary school children in Hong Kong. All subjects recruited in this study were based on a stratified protocol which stratified all 571 primary schools registered with the Education Bureau into the 7 cluster regions used by the Hospital Authority Services in Hong Kong. All children were cycloplegic for comprehensive ocular examinations and to answer standardized questionnaires according to the unified protocol. The discovery set was randomly selected from each age strata and the rest of subjects were used as replication set. |
| **Low concentration atropine for myopia progression (LAMP) study[2]** |
| LAMP is a randomized, placebo-controlled, double-masked trial, which aims to evaluate the efficacy and safety of low-concentration atropine eye drops at different concentrations. This study was conducted from January 2016 to November 2017 at the CUHK Eye Centre of the Chinese University of Hong Kong, Hong Kong, China. Children enrolled in this study were aged from 4 to 12 with myopic refraction of at least 1.0 D in both eyes, astigmatism of less than 2.5D, and documented myopic progression of at least 0.5D in the past 1 year. Written informed consent was obtained from parents or guardians, and verbal consent was obtained from the participants. The ocular examinations and cycloplegic regimen is the same with that in HKCES. |
| **Shantou Myopia Study (SMS)[3,4]** |
| The SMS was a long-term, city-wide, population-based programme which was mainly carried out by the technical institution Joint Shantou International Eye Center (JSIEC) . All schoolchildren from all primary, junior high and senior high schools in Shantou undertake vision screening once a year. The details of data collection procedures of this study can be obtained from previous study.[3,4] . The subjects used in current study were aged from 6-12. |
| **Adult-1[5]** |
| All study subjects were Chinese adults recruited from the Chinese University of Hong Kong (CUHK) Eye Centre, Hong Kong, between 2015 to 2018. All subjects underwent detailed ophthalmic assessments, including visual acuity and noncycloplegic refraction, slit lamp examination, ocular motility by Zeiss IOL Master (Carl Zeiss Meditec, Dublin, CA). We excluded those who had prior ocular surgery, or had other major eye diseases that might affect the accuracy of refraction, such as strabismus, corneal scar, keratoconus and cataract. |
| **Adult-2** |
| This is a myopia cohrot recruited from the CUHK Eye Centre, Hong Kong, between January 2016 to 2017. This cohort include myopic subjuets from mild to extreme (<-10D) myopia. Non-myopic controls had SE between +0.50D and −0.50D. All participants were free of any major eye diseases, such as keratoconus, cataract, glaucoma and age-related macular degeneration at the recruitment. |

1. Yam JC, Tang SM, Kam KW, et al. High prevalence of myopia in children and their parents in Hong Kong Chinese Population: the Hong Kong Children Eye Study. Acta Ophthalmol. 2020 Aug;98(5):e639-e648. doi: 10.1111/aos.14350.
2. Yam JC, Jiang Y, Tang SM, et al. Low-Concentration Atropine for Myopia Progression (LAMP) Study: A Randomized, Double-Blinded, Placebo-Controlled Trial of 0.05%, 0.025%, and 0.01% Atropine Eye Drops in Myopia Control. Ophthalmology. 2019 Jan;126(1):113-124. doi: 10.1016/j.ophtha.2018.05.029.
3. Wang H, Li Y, Qiu K, et al. Prevalence of myopia and uncorrected myopia among 721 032 schoolchildren in a city-wide vision screening in southern China: the Shantou Myopia Study. Br J Ophthalmol. 2023 Nov 22;107(12):1798-1805. doi: 10.1136/bjo-2021-320940.
4. Huang Y, Huang C, Li L, et al. Corneal biomechanics, refractive error, and axial length in Chinese primary school children. Invest Ophthalmol Vis Sci. 2011 Jul 1;52(7):4923-8. doi: 10.1167/iovs.10-6211.
5. Tang SM, Li FF, Lu SY, et al. Association of the ZC3H11B, ZFHX1B and SNTB1 genes with myopia of different severities. Br J Ophthalmol. 2020 Oct;104(10):1472-1476. doi: 10.1136/bjophthalmol-2019-314203.

**Table S22. Demographics of study cohorts**

| | Discovery set | | | Replication set | | |
|---|---|---|---|---|---|---|
| | **HKCES-1** | **LAMP Study** | **HKCES-2** | **SMS** | **Adult-1** | **Adult-2** |
| N | 864 | 373 | 2066 | 2027 | 1212 | 602 |
| Men (%) | 449 (52) | 207 (55) | 1116 (54) | 1065 (52.5) | 452 (37.29) | 301 (50) |
| Mean age (range, years) | 7.54 (6-9) | 8.38 (4-12) | 7.58 (5-10) | 9.63 (6-12) | 40.69 (24-69) | 43 (29-68) |
| Average SE (SD, D) | 0.32 (1.47) | -3.94 (1.83) | 0.43 (1.56) | -2.20 (1.77) | -2.79 (2.77) | -4.09 (3.47) |
| Average AL (SD, mm) | 23.05 (0.92) | 24.84 (0.93) | 23.02 (0.94) | - | - | - |

HKCES, Hong Kong children eye study; LAMP, low concentration atropine for myopia progression; SMS, Shantou Myopia Study; Adult-1, parents of children from HKCES; Adult-2, parents of children from LAMP; SE, spherical equivalent; AL, axial length; SD, standard deviation.

**Table S23. The results of Tracy-Widom test in HKCES-1 and LAMP Study for principal component adjustment**

| | HKCES | | | | LAMP | | | |
|---|---|---|---|---|---|---|---|---|
| No. | Eigenvalue | Difference | Twstat | P | Eigenvalue | Difference | Twstat | P |
| 1 | 2.03 | NA | 0.53 | **0.09** | 2.06 | NA | -2.53 | **0.85** |
| 2 | 2.01 | -0.013 | 2.41 | **0.005** | 2.03 | -0.027 | -2.12 | **0.76** |
| 3 | 1.98 | -0.036 | 4.47 | **7.77E-05** | 2.02 | -0.009 | -1.02 | **0.42** |
| 4 | 1.97 | -0.004 | 10.26 | **2.37E-11** | 2 | -0.027 | -0.19 | **0.21** |
| 5 | 1.7 | -0.269 | 7.06 | **1.17E-07** | 1.98 | -0.015 | 1.46 | **0.02** |
| 6 | 1.59 | -0.115 | -1.68 | 0.63 | 1.94 | -0.039 | 3.18 | **0.001** |
| 7 | 1.58 | -0.011 | -2.06 | 0.74 | 1.94 | -0.005 | 8.48 | **5.87E-09** |
| 8 | 1.56 | -0.016 | -3.58 | 0.98 | 1.62 | -0.314 | 0.27 | 0.12 |
| 9 | 1.56 | -0.004 | -3.3 | 0.96 | 1.6 | -0.027 | -1.74 | 0.65 |
| 10 | 1.55 | -0.002 | -2.55 | 0.86 | 1.59 | -0.003 | -0.58 | 0.3 |
| 11 | 1.54 | -0.01 | -3.08 | 0.94 | 1.58 | -0.014 | -1.04 | 0.43 |
| 12 | 1.54 | -0.007 | NA | NA | 1.57 | -0.01 | NA | NA |
| 13 | 1.53 | -0.005 | NA | NA | 1.56 | -0.01 | NA | NA |
| 14 | 1.53 | -0.003 | NA | NA | 1.55 | -0.009 | NA | NA |
| 15 | 1.52 | -0.01 | NA | NA | 1.54 | -0.008 | NA | NA |
| 16 | 1.52 | -0.003 | NA | NA | 1.54 | -0.005 | NA | NA |
| 17 | 1.51 | -0.006 | NA | NA | 1.53 | -0.01 | NA | NA |
| 18 | 1.5 | -0.013 | NA | NA | 1.51 | -0.013 | NA | NA |
| 19 | 1.47 | -0.029 | NA | NA | 1.51 | -0.006 | NA | NA |
| 20 | 1.46 | -0.009 | NA | NA | 1.5 | -0.005 | NA | NA |

No., the component number; Eigenvalue, the eigenvalue corresponding to each principal component. It represents the amount of variance explained by that principal component; Difference, the difference between consecutive eigenvalues. This difference is used to evaluate the relative importance of each principal component; Twstat (Tracy-Widom Statistic) is based on the Tracy-Widom distribution and is used to evaluate the significance of the eigenvalue.
